# Prevalence of depression, anxiety and suicidal ideas and associated factors, in particular sensory impairments, in a population of Bashkortostan in Russia

**DOI:** 10.1038/s41598-023-44561-1

**Published:** 2023-10-12

**Authors:** Mukharram M. Bikbov, Timur R. Gilmanshin, Gyulli M. Kazakbaeva, Ellina M. Iakupova, Songhomitra Panda-Jonas, Rinat M. Zainullin, Albina A. Fakhretdinova, Azaliia M. Tuliakova, Leisan I. Gilemzianova, Dinar A. Khakimov, Liana A. Miniazeva, Jost B. Jonas

**Affiliations:** 1 Ufa Eye Research Institute, 90 Pushkin Street, Ufa, 450077 Russia; 2 Ufa Eye Institute, Ufa, Russia; 3 Privatpraxis Prof Jonas und Dr Panda-Jonas, Heidelberg, Germany; 4 Department of Ophthalmology, University Hospital Heidelberg, Heidelberg, Germany; 5 Department of Ophthalmology, Medical Faculty Mannheim, Heidelberg University, Theodor-Kutzerufer 1, 68167 Mannheim, Germany; 6 Institute of Molecular and Clinical Ophthalmology Basel, Basel, Switzerland

**Keywords:** Diseases of the nervous system, Anxiety, Depression

## Abstract

To assess prevalence and associated factors of depression, anxiety and suicidal ideas in populations from Russia, we conducted in rural and urban regions in Bashkortostan/Russia two population-based studies (Ural Eye and Medical Study (UEMS), performed from 2015 to 2017; Ural Very Old Study (UVOS), performed from 2017 to 2020) which included participants aged 40 + years and 85 + years, respectively. Depression was assessed using the questionnaire of the Center for Epidemiologic Studies Depression Scale Scoresheet, and anxiety was examined applying the State Trait Inventory Anxiety Test. Suicidal ideas were explored by the question whether suicide had previously been thought of or attempted (and if yes, for what reasons). In the statistical analysis we assessed the mean of the main outcome parameter (depression score and anxiety score) and searched for associations between these parameters and other parameters in univariable and multivariable regression analyses. In the UEMS with 5893 individuals (age: 59.0 ± 10.7 years; range 40–94 years), higher depression score and anxiety score were associated (multivariable analysis) with more marked hearing loss (beta: 0.07; *P* < 0.001, and beta: 0.07; *P* < 0.0012, respectively) and worse visual acuity (beta: 0.04; *P* = 0.02; and beta: 0.03; *P* = 0.03, resp.), in addition to female sex, Russian ethnicity, lower educational level, less alcohol consumption, weaker hand grip strength, less physical activity, and higher prevalence of dry eye disease. Attempted suicide was reported by 88 (1.5%; 95% CI 1.2, 1.8) participants. Having thought of suicide within the last 6 months was reported by 63 (1.1%) individuals. Out of 1491 UVOS participants (age: 88.2 ± 2.8 years; range 85–100 years) with a mean depression score of 20.0 ± 10.3 (median 18; range 0–58), 916 (61.4%; 95% CI 59.0, 63.9) fulfilled the definition of depression (depressions core ≥ 16). Higher depression score and higher anxiety score correlated (multivariable analysis) with higher hearing loss score (beta: 0.07; *P* = 0.02, and beta: 0.08; *P* = 0.009, resp.) and worse visual acuity (beta: 0.13; *P* < 0.001, and beta: 0.09; *P* = 0.007, resp.), in addition to female sex, urban region, less physical activity, less fruit intake, and lower cognitive function. Overall, 15 (1.0%; 95% CI 0.50, 1.50) individuals had attempted or thought of suicide. In conclusion, the findings suggest that besides female sex, lower level of education and lower cognitive function, it was sensory impairment, namely vision and hearing impairment, which belonged to the determinants of depression and anxiety in these populations from Russia.

## Introduction

The Global Burden of Diseases, Injuries, and Risk Factors Study 2019 revealed that depression and anxiety belong to the 25 most common causes of health-related burden worldwide^1–4^. Depression with a lifetime prevalence of 2% to 15% is one of the most important risk factors for substantial disability. Anxiety disorders are common and disabling conditions which predominantly start during childhood, adolescence, and early adulthood and which affect almost twice as many women as men^5^. They often concur with depression, alcohol and other substance-use disorders, and personality disorders. Suicide and self-harm are other major health and societal problems globally, with the highest impact in low-income and middle-income regions^6^. Rather than having a single cause, suicide and self-harm are the result of a complex interplay of several factors that occur throughout the life course, and vary by sex, age, ethnicity, and geography.

Prevalence and associations of depression and anxiety in the regions of Central Asia and Russia have relatively rarely been examined so far^7–12^. Averina and colleagues examined in a population-based study in Arkhangelsk, Russia, in the period 1999–2000 more than 4500 participants aged 18–90 years and found a higher prevalence of self-reported depression, anxiety and/or sleeping disorders in women (68.7%) than in men (32.3%)^7^. A higher prevalence of depression was correlated with smoking and alcohol consumption. Gurina and coworkers examined community-dwelling adults aged 65 + years in a suburb of St. Petersburg and reported that 25.9% of the younger group (age 65–74 years) and 42.5% of the older group (age ≥ 75 years) were at risk for depression^8^. Cook assessed individuals 35–69 years old and recruited in a population-based manner in Arkhangelsk and Novosibirsk in the study period from 2015 to 2018^9^. The prevalence of depression was 10.7% in women and 5.4% in men, and the prevalence of anxiety was 10 6.2% in women and 3.0% in men. These previous investigations had limitations, such as being focused mostly on urban populations, with the recruitment of their participants not fully population-based, and/or addressing only the main disorders of depression and/or anxiety and not including many additional diseases and parameters into their study design. It holds true in particular for associations between depression/anxiety and hearing and vision impairment. We therefore examined in a population-based manner the prevalence of depression and anxiety and their associations with a whole array of other diseases and parameters in populations from Russia.

## Methods

### Ural eye and medical study (UEMS)

The UEMS is a population-based study which was performed in the Russian republic of Bashkortostan at the southwestern end of the Ural Mountains in the study period from 2015 to 2017^13,14^. Study regions were the urban region of the Kirovskii district in Ufa as the capital of Bashkortostan and a rural region in the Karmaskalinsky District in a distance of 65 km from Ufa. The republic of Bashkortostan is situated between the Volga River and the Ural Mountains and is, with a population of 4 million people, the most populous republic in Russia. Inclusion criteria for the study were living in the study regions and an age of 40 + years. There were no exclusion criteria. Out of a total group of 7328 eligible individuals, 5899 (80.5%) individuals (3319 [56.3%] women) with a mean age of 59.0 ± 10.7 years (range 40–94 years) participated in the study. The demographics and general characteristics of the population of the UEMS have described in detail recently^13,14^.

### Ural very old study (UVOS)

The UVOS is also a population-based study with the inclusion criteria of an age of 85 + years and living in the study regions. There were no exclusion criteria. The UVOS was conducted in the same study regions as the UEMS, i.e., the urban Kirovskii district in Ufa and the rural region Karmaskalinsky District. Due to the stricter inclusion criterion of a minimal age of 85 years, the size of both study regions included into the investigation was larger in the UVOS than in the UEMS. The UVOS was carried out in the period from 2017 to 2020^15^. Out of 1882 eligible inhabitants aged 85 + years and living in the study regions, 1526 (81.1%) persons participated in the UVOS. The eligible individuals also included the inhabitants of all three private small retirement homes located in the urban study region. The characteristics and demographic parameters of the UVOS population have described in detail recently^15^. The study populations of the UEMS and UVOS did not differ significantly in the sex and age distribution from the Russian population as explored in the census carried out in 2010^16^. Both studies, the UEMS and the UVOS, were performed by the same examination team applying the same techniques and using the same devices. The designs of both studies were similar or almost identical, except for the age-related inclusion criterion. The reason to choose an age of 40 + years as inclusion criterion for the UVOS was to examine the prevalence of major disorders and their associated factors in a middle-aged and elderly population. After finishing the recruitment for the UEMS, it was realized that in the UEMS, as in almost any other population-based study with such an age criterion for participation, the fraction of the very old group was relatively small. It was then decided to conduct a second study, now focusing on the very old population, and we set an inclusion criterion of an age of 85 + years.

### Ethics

The Ethics Committee of the Academic Council of the Ufa Eye Research Institute approved the UEMS and the UVOS, and informed written consent was obtained from all participants. If individuals could not fully understand or appeared to be not fully informed about the meaning of the survey and the examination procedure applied in the study, family members or study personal repeatedly explained it to them in separate sessions until the participants were aware of the study. If the individuals were cognitively markedly impaired, informed consent was additionally obtained from family members. All the methods from both the studies were performed in accordance with relevant guidelines and regulation.

### Methods

Using a bus, the study participants in both studies were brought from their homes to the Ufa Eye Institute where a team of about 20 trained medical doctors and technicians performed all examinations. The data were collected and stored in datafiles by a team of statisticians. Those UVOS participants being too immobile for the transport, were examined at their homes. The series of examinations started with a detailed interview consisting of more than 250 standardized questions on the socioeconomic background, diet, smoking, alcohol consumption, physical activity, quality of life and vision, history of any type of injuries and inter-personal violence, health assessment questions, medical history including known diagnosis and therapy of major disorders, symptoms of chronic diseases such as chronic obstructive pulmonary disease, asthma, kidney disease, orthopedic disorders, epilepsy and polyneuropathy, cognitive function and hearing loss (Table 1) (Supplementary Material)^13,15^. The level of education was differentiated into 8 grades, including “illiterate”, “passed the 5th class”, “passed the 8th class”, “passed the 10th class”, “passed the 12th class”, “specialized secondary education”, “graduation”, and “post-graduation”. The questions included in the interview were taken from standardized interviews published in the literature, such as the National Eye Institute Visual Functioning Questionnaire-25 (VFQ-25), the Questionnaire for Verifying Stroke-Free Status (QVSFS) from the American Heart Association, and the Michigan Neuropathy Screening Instrument^13,15^. Cognitive function was examined applying Folstein´s Mini-Mental State Examination (MMSE) with a maximal score of 30 points^17^. The examinations further included anthropometry, blood pressure measurement, handgrip dynamometry, spirometry, biochemical analysis of blood samples taken under fasting conditions, measurement of visual acuity based on automatic and subjective refractometry, and examination of prevalence and degree of a dry eye disease (Table 1). As described previously, dry eye disease was assessed by specific questions in the questionnaire and by additional ophthalmological examinations including Schirmer’s test^18,19^. The Guidelines for Accurate and Transparent Health Estimates Reporting (GATHER statement guidelines) for collecting the data were applied^20^

**Table 1:** Associations (univariate analysis) between the depression score and other parameters in the Ural eye and medical study.

	Standardized regression coefficient beta	Non-standardized regression coefficient B	95% confidence interval of B	*P*-value
Age (years)	0.12	0.04	0.03, 0.05	< 0.001
Sex (men/women)	0.22	1.67	1.49, 1.86	< 0.001
Region of habitation (rural/urban)	0.05	0.35	0.16, 0.54	< 0.001
Ethnicity (non-Russian/Russian)	0.05	0.48	0.23, 0.72	< 0.001
Body height (cm)	− 0.20	− 0.09	− 0.10, − 0.07	< 0.001
Body weight (kg)	− 0.09	− 0.02	− 0.03, − 0.02	< 0.001
Body mass index (kg/m^2^)	003	0.02	0.002, 0.04	0.03
Waist circumference (cm)	− 0.03	− 0.01	− 0.02, − 0.001	0.02
Hip circumference (cm)	0.02	0.007	0.000, 0.02	0.07
Waist/hip circumference ratio	− 0.08	− 3.13	− 4.18, − 2.09	< 0.001
Level of education (1–8)	− 0.09	− 0.24	− 0.30, − 0.17	< 0.001
Smoking, currently (no/yes)	− 0.06	− 0.71	− 0.99, − 0.42	< 0.001
Smoking package years	− 0.06	− 0.02	− 0.02, − 0.01	< 0.001
Alcohol consumption, any (no/yes)	− 0.003	− 0.03	− 0.26, 0.21	0.83
Number of daily meals	0.02	0.10	− 0.02, 0.22	0.12
In a week how many days do you eat fruits?	− 0.10	− 0.19	− 0.24, − 0.14	< 0.001
In a week how many days do you eat vegetables?	− 0.09	− 0.24	− 0.30, − 0.17	< 0.001
Type of oil for cooking used: vegetable cooking oil – animal fat (butter)	0.02	0.28	− 0.09, 0.66	0.14
Food containing whole grains (no/yes)	− 0.05	− 0.47	− 0.72, − 0.22	< 0.001
Self-reported salt consumed per day (g)	− 0.05	− 0.07	− 0.12, − 0.03	0.001
Degree of processing meat (weak – medium – strong)	− 0.01	− 0.10	− 0.29, 0.09	0.31
Length of working day (hours)	− 0.09	− 0.001	− 0.002, − 0.001	< 0.001
Do you mostly sit and walk less than 10 min (no/yes)	0.005	0.04	− 018, 0.26	0.72
Work with moderate to vigorous physical activity (no/yes)	− 0.01	− 0.03	− 0.24, 0.17	0.74
How many days per week with vigorous physical work	0.06	0.14	0.05, 0.22	0.001
How much time spent with vigorous physical work per day	− 0.10	− 0.001	− 0.002, − 0.001	< 0.001
Work with moderate physical activity (no/yes)	− 0.04	− 0.35	− 0.56, − 0.13	0.002
How many days per week with moderate physical activity at work	0.05	0.12	0.04, 0.20	0.004
How much time spent with moderate physical activity at work per day	− 0.08	− 0.001	− 0.002, − 0.001	< 0.001
Walking or biking for at least 10 min per day (no/yes)	− 0.01	− 0.16	− 0.47, 0.15	0.30
How many days per week with walking or biking for at least 10 min per day	− 0.05	− 0.13	− 0.21, − 0.06	< 0.001
Recreation and leisure time spent mostly with sitting (no/yes)	− 0.04	− 0.32	− 0.52, − 0.12	0.002
In your leisure time, do you do any physically vigorous activities like running, strenuous sports or weight lifting for at least 10 min at a time? (no/yes)	− 0.09	− 0.70	− 0.91, − 0.49	< 0.001
In your leisure time, do you do any moderate intensity activities like brisk walking, cycling or swimming for at least 10 min at a time? (no/yes)	− 0.08	− 0.58	− 0.77, − 0.39	< 0.001
Over the past 7 days, how much time did you spend sitting or reclining on a typical day? (minutes)	0.08	0.000	0.000, 0.000	< 0.001
History of angina pectoris (no/yes)	0.08	1.02	0.68, 1.35	< 0.001
History of asthma (no/yes)	0.05	1.19	0.60, 1.77	< 0.001
History of arterial hypertension (no/yes)	0.12	0.91	0.72, 1.10	< 0.001
History of arthritis (no/yes)	0.11	0.94	0.72, 1.15	< 0.001
History of previous bone fractures (no/yes)	0.04	0.33	0.11, 0.55	0.003
History of low back pain (no/yes)	0.13	0.99	0.79, 1.19	< 0.001
History of thoracic spine pain (no/yes)	0.17	1.29	1.09, 1.49	< 0.001
History of neck pain (no/yes)	0.14	1.17	0.95, 1.39	< 0.001
History of headache (no/yes)	0.17	1.29	1.09, 1.49	< 0.001
History of cancer (no/yes)	0.04	0.92	0.36, 1.48	0.001
History of cardiovascular disorders including stroke (no/yes)	0.15	1.25	1.02, 1.47	< 0.001
History of heart attack (no/yes)	0.03	0.44	0.01, 0.86	0.04
History of dementia (no/yes)	0.04	1.62	0.41, 2.84	0.009
History of diabetes mellitus (no/yes)	0.06	0.80	0.46, 1.15	< 0.001
History of diarrhea (no/yes)	0.04	2.27	0.85, 3.69	0.002
History of iron-deficiency anemia (no/yes)	0.08	1.37	0.94, 1.80	< 0.001
History of low blood pressure and hospital admittance (no/yes)	0.06	1.17	0.66, 1.68	< 0.001
History of osteoarthritis (no/yes)	0.07	0.71	0.45, 0.97	< 0.001
History of skin disease (no/yes)	007	1.13	0.68, 1.57	< 0.001
History of thyroid disease (no/yes)	0.11	1.40	1.09, 1.71	< 0.001
History of falls (no/yes)	0.11	1,.08	0.84, 1.32	< 0.001
History of unconsciousness (no/yes)	0.13	1.74	1.40, 2.08	< 0.001
History of menopause (no/yes)	0.06	0.60	0.25, 0.95	0.001
Age of the last regular menstrual bleeding (years)	− 0.02	− 0.02	− 0.05, 0.01	0.26
Age of last menstrual bleeding (years)	− 0.02	− 0.01	− 0.04, 0.02	0.45
Alanine aminotransferase (IU/L)	0.01	0.004	− 0.004, 0.01	0.29
Aspartate aminotransferase (IU/L)	− 0.004	− 0.001	− 0.01, 0.01	0.78
Aspartate aminotransferase/alanine aminotransferase ratio	− 0.01	− 0.17	− 0.51, 0.17	0.33
Bilirubine, total (µmol/L)	− 0.03	− 0.01	− 0.02, − 0.003	0.009
High-density lipoproteins (mmol/L)	0.05	0.19	0.08, 0.30	0.001
Low-density lipoproteins (mmol/L)	− 0.02	− 0.05	− 0.13, 0.04	0.27
Cholesterol (mmol/L)	0.01	0.02	− 0.04, 0.08	0.49
Triglycerides (mmol/L)	0.01	0.03	− 0.11, 0.16	0.72
Rheumatoid factor (IU/mL)	0.03	0.10	− 0.003, 0.20	0.06
Erythrocyte sedimentation rate (mm/hour)	0.10	0.03	0.02, 0.04	< 0.001
Glucose (mmol/L)	0.03	0.06	0.003, 0.12	0.04
Creatinine (µmol/L)	− 0.06	− 0.01	− 0.01, − 0.01	< 0.001
Estimated glomerular filtration rate (mL/min per 1.73 m^2^)	− 0.07	− 0.01	− 00.2, − 0.01	< 0.001
Chronic kidney disease stage	0.06	0.03	0.02, 0.04	< 0.001
Urea (mmol/L)	0.05	0.12	0.06, 0.19	< 0.001
Residual nitrogen (g/L)	0.02	1.08	− 0.24, 2.41	0.11
Total protein (g/L)	− 0.004	− 0.002	− 0.02, 0.01	0.79
International normalized ratio (INR)	− 0.02	− 0.63	− 1.29, 0.04	0.06
Blood clotting time (minutes)	0.06	0.43	0.25, 0.61	< 0.001
Prothrombin time (%)	0.02	0.01	− 0.002, 0.02	0.14
Hemoglobin	− 0.11	− 0.03	− 0.04, − 0.02	< 0.001
Erythrocytes (10^6^ cells/µL)	− 0.12	− 1.15	− 1.40, − 0.90	< 0.001
Leukocytes (10^9^ cells/L)	0.001	0.004	− 0.06, 0.07	0.91
Rod-core granulocyte (% of leukocytes)	004	0.10	0.03, 0.17	0.006
Segment nuclear granulocyte (% of leukocytes)	− 0.06	− 0.03	− 0.04, − 0.02	< 0.001
Eosinophil granulocytes (% of leukocytes)	0.05	0.15	0.06, 0.25	0.002
Lymphocytes (% of leukocytes)	0.02	0.01	− 0.003, 0.03	0.12
Monocytes (% of leukocytes)	0.04	0.06	0.02, 0.11	0.003
Prevalence of diabetes mellitus	0.05	0.59	0.29, 0.89	< 0.001
Anemia (serum hemoglobin concentration < 140 g/L in men, < 130 g/L in women)	0.04	0.35	0.12, 0.57	0.002
Blood pressure, systolic (mm Hg)	0.05	0.01	0.01, 0.01	< 0.001
Blood pressure, diastolic (mm Hg)	− 0.001	0.000	− 0.009, 0.009	0.95
Blood pressure, mean (mm Hg)	0.03	0.01	0.001, 0.02	0.03
Arterial hypertension (no/yes)	0.01	0.11	− 0.16, 0.37	0.42
Arterial hypertension, stages	0.02	0.06	− 0.03, 0.16	0.18
Ankle-brachial index, right	0.02	0.42	− 0.28, 1.11	0.24
Ankle-brachial index, left	0.02	0.49	− 0.21, 1.18	0.17
Prevalence of chronic obstructive pulmonary disease (no/yes)	0.07	0.97	0.58, 1.37	< 0.001
Metabolic syndrome (no/yes)	0.05	0.40	0.18, 0.62	< 0.001
Non-alcoholic fatty liver disease (no/yes)	− 0.003	− 0.002	− 0.22, 0.17	0.83
Hearing loss score	0.12	0.04	0.03, 0.05	< 0.001
Visual acuity (best corrected; better eye) (LogMAR (logarithm of the minimal angle of resolution))	0.08	1.31	0.87, 1.76	< 0.001
Dry Eye (no/yes)	0.06	0.93	0.52, 1.35	< 0.001
Ocular axial length (mm)	− 0.07	− 0.23	− 0.31, − 0.14	< 0.001
State-trait anxiety Inventory	0.73	0.77	0.75, 0.79	< 0.001
Manual dynamometry, right hand (dekaNewton)	− 0.25	− 0.08	− 0.09, − 0.07	< 0.001
Manual dynamometry, left hand (dekaNewton)	− 0.25	− 0.08	− 0.09, − 0.08	< 0.001

For all categorical parameters, the first mentioned gradation is the reference category (i.e., in the case of “men/women”, reference category is “men”; in the case of “no/yes”, reference category is “no”).

Depression was assessed using the questionnaire of the Center for Epidemiologic Studies Depression Scale (CES-D) Scoresheet, and we examined anxiety using the State Trait Inventory Anxiety Test^21–24^. The participants of the UVOS could select one of four replies [“rarely or none of the time (less than one day)”/“some or a little of the time (1–2 days)”,/“occasionally or a moderate amount of time (3–4 days)”/“most or all of the time (5–7 days)”]. The participants of the UEMS had the choice between two replies (“some or a little of the time (1–2 days) or more rarely” versus “occasionally or a moderate amount of time (3–4 days) or more frequently”). Suicidal ideas were assessed by the question whether suicide had previously been attempted (and if yes, for what reasons including financial reasons), and whether one had thought of suicide and for what reasons.

### Statistical analysis

Inclusion criteria for the present study were the availability of measurements of the depression score and anxiety score. Using a statistical software package (SPSS for Windows, version 27.0, IBM-SPSS, Chicago, IL, USA), we assessed the mean values and standard deviations main outcome parameters, i.e., the depression score and anxiety score and searched for associations between these parameters and other parameters, first in univariable linear regression analyses and followed by multivariable linear regression analyses. For that purpose, the values of categorical parameters with more than two possible values (e.g., “degree of processing meat (weak–medium–strong)”) were converted into points (i.e., weak—1, medium—2, strong—3). The multivariable regression analysis included the depression score or the anxiety score as dependent variable and as independent parameters all those variables which were associated with the depression score or the anxiety score in the univariable analysis with a *P*-value of ≤ 0.10. We chose a cut-off value of ≤ 0.10 to reduce the risk of missing a parameter the association of which might have become statistically significant in the eventual multivariable analysis. In a step-by-step manner, we first dropped parameters with collinearity (i.e., a variance inflation factor (VIF) of > 3), before we dropped those parameters out of the list of independent parameters that were no longer significantly associated with the depression score or anxiety score. We then added parameters, which previously dropped out, again to the model to test for the significance of their potential association with the depression and anxiety score. The prevalence of parameters such as depression was described as mean and its 95% confidence intervals (CIs). We calculated the standardized regression coefficient beta, the non-standardized regression coefficient B and its 95%CIs. All *P*-values were two-sided and were considered statistically significant when the values were less than 0.05.

## Results

### Ural eye and medical study (UEMS)

Out of the 5899 participants of the UEMS, the present investigation included 5893 (99.0%) individuals (3315 [56.3%] women) with measurements of the depression score and anxiety score. The mean age was 59.0 ± 10.7 years (median: 58 years; range 40–94 years). The group of study participants as compared with the group of individuals without measurement of the depression score and anxiety score did not differ significantly in age (*P* = 0.28) and sex (*P* = 0.61).

The mean depression score (theoretical maximum value: 20) was 5.2 ± 3.7 (median: 5; range 0–19), and the mean anxiety score (theoretical maximum value: 20) was 6.3 ± 3.5 (median: 6; range 0–20). Using a cut-off value of 6 or higher for the definition of depression, 2443/5893 (41.5%; 95% CI 40.2, 42.7) participants had depression.

In univariable analysis, a higher depression score was associated (*P* ≤ 0.1) with a multitude of parameters including age (beta: 0.12; B: 0.04; 95% CI 0.03, 0.05; *P* < 0.001) and sex (beta: 0.22; B: 1.67; 95% CI 1.49, 1.86; *P* < 0.001) (Table 1). In the multivariable regression analysis with the depressions core as dependent variable, we dropped various parameters due to collinearity or due to lack of statistical significance. In the final model, a higher depression score was associated with female sex, Russian ethnicity, lower level of education, higher prevalence of any alcohol consumption, lower number of days with fruit intake, lower self-reported salt consumption, higher prevalence of a history of angina pectoris, headache and thoracic spine pain, cardiovascular disease including stroke, skin diseases, thyroid gland disorders, falls and unconsciousness, lower dynamometric hand grip force, shorter duration of working day, lower prevalence of moderate physical activity in leisure time, lower number of days per week with walking or using a bicycle for at least 10 min to go to and from places, higher hearing loss score, worse best corrected visual acuity binocular or in the better eye, and higher prevalence of dry eye disease (Table 2). When we added the parameters of age (*P* = 0.99), region of habitation (*P* = 0.42), systolic blood pressure (*P* = 0.55), stage of arterial hypertension (*P* = 0.83), body height (*P* = 0.39), body mass index (*P* = 0.41), waist-hip circumference ratio (*P* = 0.24), waist-height ratio (*P* = 0.07), current smoking (*P* = 0.58), smoking package years (*P* = 0.14), self-reported salt intake (*P* = 0.06), and food containing whole grain (*P* = 0.90) as single parameters to the model, they were not significantly associated with the depression score.

**Table 2:** Associations (multivariable analysis) between the depression score [correlation coefficient r = 0.38 (r^2^ = 0.14)] and other parameters in the Ural eye and medical study.

	Standardized regression coefficient beta	Non- standardized regression coefficient B	95% confidence interval of B	*P*-value	Variance inflation factor
Sex (men/women)	0.12	0.84	0.50, 1.18	< 0.001	2.41
Russian ethnicity (no/yes)	0.03	0.31	0.02, 0.57	0.04	1.02
Level of education (1–8)	− 0.06	− 0.17	− 0.25, − 0.08	< 0.001	1.13
Alcohol consumption, any (no/yes)	0.10	0.79	0.54, 1.04	< 0.000	1.06
Number of days per week with fruit intake	− 0.06	− 0.12	− 0.17, − 0.06	< 0.001	1.05
Salt consumption, self-reported (gram)	− 0.03	− 0.05	− 0.10, − 0.002	0.04	1.07
History of angina pectoris (no/yes)	0.04	0.54	0.14, 0.94	0.008	1.04
History of headache (no/yes)	0.06	0.41	0.18, 0.64	0.001	1.12
History of thoracic spine pain (no/yes)	0.10	0.91	0.64, 1.19	< 0.001	1.11
History of cardiovascular disease including stroke (no/yes)	0.03	0.29	0.02, 0.57	0.03	1.12
History of skin diseases (no/yes)	0.04	0.72	0.21, 1.23	0.006	1.02
History of thyroid gland disorders (no/yes)	0.04	0.49	0.08, 0.90	0.02	1.08
History of falls (no/yes)	0.05	0.47	0.16, 0.77	0.003	1.07
History of unconsciousness (no/yes)	0.08	1.08	0.64, 1.53	< 0.001	1.08
Hand grip force (deka newton)	− 0.11	− 0.04	− 0.05, − 0.02	< 0.001	2.46
Length of working day	− 0.04	− 0.001	− 0.001, 0.000	0.02	1.11
Moderately intensive physical activity in leisure time (no/yes)?	− 0.06	− 0.43	− 0.65, − 0.20	< 0.001	1.06
In a typical week, on how many days do you walk or use a bicycle for at least 10 min to go to and from places?	− 0.03	− 0.09	− 0.17, − 0.002	0.04	1.03
Hearing loss score	0.07	0.02	0.01, 0.03	< 0.001	1.08
Visual acuity, binocular or better eye best corrected (LogMAR (logarithm of the minimal angle of resolution))	0.04	0.82	0.16, 1.48	0.02	1.11
Dry eye disease (prevalence) (no/yes)	0.05	0.86	0.03, 1.39	0.001	1.02

For all categorical parameters, the first mentioned gradation is the reference category (i.e., in the case of “men/women”, reference category is “men”; in the case of “no/yes”, reference category is “no”).

A higher anxiety score correlated (multivariable analysis) with female sex, higher waist-hip circumference ratio, lower level of education, higher prevalence of any alcohol consumption, lower number of days with fruit intake, higher prevalence of a history of headache, thoracic spine pain and backache, cardiovascular disease including stroke, skin diseases, thyroid gland disorders, and unconsciousness, lower dynamometric hand grip force, lower prevalence of moderate physical activity in leisure time, higher prevalence of chronic obstructive pulmonary disease, higher hearing loss score, lower best corrected visual acuity, and higher prevalence of dry eye disease (Table 3). When we added the parameters of age (*P* = 0.15), region of habitation (*P* = 0.45), systolic blood pressure (*P* = 0.33), stage of arterial hypertension (*P* = 0.39), body mass index (*P* = 0.60), waist-height ratio (*P* = 0.12), current smoking (*P* = 0.94), smoking package years (*P* = 0.52), self-reported salt intake (*P* = 0.12), and food containing whole grain (*P* = 0.32) as single parameters to the model, they were not significantly associated with the depression score.

**Table 3:** Associations (multivariable analysis) between the anxiety score (correlation coefficient r = 0.40) and other parameters in the Ural eye and medical study.

Sex (men/women)	0.18	1.27	0.99, 1.55	< 0.001	2.45
Waist-hip circumference ratio	0.05	1.81	0.78, 2.83	0.001	1.13
Level of education (1–8)	− 0.06	− 0.15	− 0.22, − 0.09	< 0.001	1.14
Alcohol consumption, any (no/yes)	0.08	0.63	0.41, 0.84	< 0.001	1.08
Number of days per week with fruit intake	− 0.08	− 0.14	− 0.19, − 0.10	< 0.001	1.06
History of headache (no/yes)	0.08	0.53	0.34, 0.72	< 0.001	1.14
History of thoracic spine pain (no/yes)	0.09	0.77	0.55, 0.998	< 0.001	1.18
History of backache (no/yes)	0.03	0.23	0.04, 0.43	0.02	1.18
History of cardiovascular disease including stroke (no/yes)	0.05	0.36	0.15, 0.57	0.001	1.11
History of skin diseases (no/yes)	0.04	0.66	0.26, 1.05	0.001	1.02
History of thyroid gland disorders (no/yes)	0.04	0.46	0.14, 0.77	0.004	1.09
History of unconsciousness (no/yes)	0.05	0.64	0.31, 0.97	< 0.001	1.05
Hand grip force (dekaNewton)	− 0.09	− 0.03	− 0.04, − 0.02	< 0.001	2.33
Moderately intensive physical activity in leisure time (no/yes)?	− 0.07	− 0.52	− 0.70, − 0.34	< 0.001	1.03
Chronic obstructive pulmonary disease	0.03	0.42	0.06, 0.78	0.02	1.02
Hearing loss score	0.07	0.02	0.01, 0.03	< 0.001	1.06
Visual acuity (LogMAR) (logarithm of the minimal angle of resolution)	0.03	0.24	0.02, 0.46	0.03	1.06
Dry eye disease (prevalence) (no/yes)	0.05	0.74	0.33, 1.15	< 0.001	1.02

For all categorical parameters, the first mentioned gradation is the reference category (i.e., in the case of “men/women”, reference category is “men”; in the case of “no/yes”, reference category is “no”).

Out of the 5893 participants of the UEMS, 88 (1.5%; 95% CI 1.2, 1.8) reported to have attempted suicide, with 20 individuals out of the 88 indicating financial reasons for their attempt. Having thought of suicide was reported by 63 (1.1%) individuals, with 37 (0.6%) study participants stating, that they thought so within the last 6 months. Overall, 131 (2.2%; 95% CI 1.9, 2.6) individuals attempted or thought of suicide. A higher prevalence of having thought of or having attempted suicide correlated with younger age, lower level of education, lower number of days with fruit intake, higher prevalence of reported thoracic spine pain, unconsciousness and vigorous activity in leisure time, lower hand grip force, higher hearing loss score, and higher prevalence of dry eye disease (Table 4). When we replaced the parameter of hand grip force with the parameter of sex, female sex was associated with a higher prevalence of suicidal thoughts/attempts (OR 2.41; 95% CI 1.52, 3.80). When we added the parameters of region of habitation (*P* = 0.71), systolic blood pressure (*P* = 0.57), stage of arterial hypertension (*P* = 0.98), body mass index (*P* = 0.91), waist-hip ratio (*P* = 0.17), waist-height ratio (*P* = 0.97), current smoking (*P* = 0.64), smoking package years (*P* = 0.76), self-reported salt intake (*P* = 0.07), and food containing whole grain (*P* = 0.19) as single parameters to the model, they were not significantly associated with the prevalence of having thought of or having attempted suicide.

**Table 4:** Associations (multivariable analysis) between the prevalence of attempted suicide or thoughts of attempting suicide and other parameters in the Ufa eye and medical study.

	Odds ratio	95% confidence interval	*P*-value
Age (years)	0.94	0.92, 0.96	< 0.001
Level of education (1–8)	0.85	0.74, 0.99	0.03
Number of days with fruit intake per week	0.85	0.78, 0.94	0.001
History of thoracic spine pain (no/yes)	2.04	1.35, 3.08	0.001
History of unconsciousness (no/yes)	2.79	1.67, 4.64	< 0.001
Vigorous activity in leisure time	1.77	1.16, 2.70	0.008
Hand grip force (dekaNewton)	0.96	0.94, 0.98	< 0.001
Hearing loss score	1.02	1.00, 1.04	0.03
Dry eye disease (prevalence) (no/yes)	2.42	1.33, 4.41	0.004

For all categorical parameters, the first mentioned gradation is the reference category (i.e., in the case of “men/women”, reference category is “men”; in the case of “no/yes”, reference category is “no”).

### Ural very old study (UVOS)

Out of 1526 participants of the Ural Very Old Study, measurements of the depression score and anxiety score were available for 1491 (97.7%) individuals [382 (25.6%) men] with a mean age of 88.2 ± 2.8 years (range 85–100 years). The group of study participants as compared with the group of individuals without measurement of the depression and anxiety scores was significantly younger (88.2 ± 2.8 years versus 88.6 ± 3.1 years; *P* = 0.03), while both groups did not differ significantly in sex (*P* = 0.22).

The mean depression score (theoretical maximal value: 60) was 20.0 ± 10.3 (median: 18; range 0–58). Using a cut-off value of 16 or higher for the definition of depression, 916/1491 (61.4%; 95% CI 59.0, 63.9) participants had depression. A higher depression score was associated with female sex, urban region of habitation, lower number of days with fruit intake, lower prevalence of vigorous activity in leisure time, higher prevalence of a history of headache, higher hearing loss score, worse visual acuity, lower cognitive function, and lower prevalence of self-reported chronic obstructive pulmonary disease (Table 5). When we added the parameters of age (*P* = 0.78), systolic blood pressure (*P* = 0.40), stage of arterial hypertension (*P* = 0.23), body height (*P* = 0.051), body mass index (*P* = 0.73), waist-hip circumference ratio (*P* = 0.08), waist-height ratio (*P* = 0.62), current smoking (*P* = 0.32), self-reported salt intake (*P* = 0.92), number of cups of coffee taken daily (*P* = 0.86), number of cups of tea taken daily (*P* = 0.24), preference of black or green tea (*P* = 0.47), and food containing whole grain (*P* = 0.39) as single parameters to the model, they were not significantly associated with the depression score.

**Table 5:** Associations (multivariable analysis) between the depression score (correlation coefficient r = 0.50) and other parameters in the Ural very old study.

	Standardized regression coefficient beta	Non-standardized regression coefficient B	95% confidence interval of B	*P*-value	Variance inflation factor
Sex (men/women)	0.12	2.75	1.37, 4.13	< 0.001	1.06
Rural/urban region of habitation	0.14	3.31	1.75, 4.87	< 0.001	1.32
Number of days with fruit intake per week	− 0.14	− 0.71	− 1.01, − 0.40	< 0.001	1.19
Vigorous activity in leisure time	− 0.08	− 3.02	− 5.19, − 0.86	0.006	1.03
History of headache (no/yes)	0.11	2.30	1.07, 3.54	< 0.001	1.08
Hearing loss score	0.07	0.05	0.01, 0.09	0.02	1.11
Visual acuity (best corrected, binocular)	0.13	1.76	0.92, 2.61	< 0.001	1.11
Chronic obstructive pulmonary disease (no/yes)	− 0.07	− 2.74	− 4.96, − 0.52	0.02	1.07
Cognitive function	− 0.34	− 0.56	− 0.66, − 0.45	< 0.001	1.21

For all categorical parameters, the first mentioned gradation is the reference category (i.e., in the case of “men/women”, reference category is “men”; in the case of “no/yes”, reference category is “no”).

A higher anxiety score correlated with female sex, urban region of habitation, lower number of days with fruit intake, more time spent with sitting or reclining, less vigorous physical activity in leisure time, higher prevalence of a history of headache, higher hearing loss score, worse best corrected visual acuity, higher prevalence of chronic obstructive pulmonary disease, a lower cognitive function score (Table 6). When we added the parameters of age (P = 0.18), systolic blood pressure (P = 0.43), stage of arterial hypertension (P = 0.33), body mass index (P = 0.61), waist-hip circumference ratio (P = 0.15), waist-height ratio (P = 0.79), current smoking (P = 0.54), self-reported salt intake (P = 0.75), number of cups of coffee taken daily (P = 0.56), number of cups of tea taken daily (P = 0.95), preference of black or green tea (P = 0.70), and food containing whole grain (P = 0.57) as single parameters to the model, they were not significantly associated with the depression score.

**Table 6:** Associations (multivariable analysis) between the anxiety score [correlation coefficient r = 0.47 (r^2^ = 0.22)] and other parameters in the Ural Very Old Study.

	Standardized regression coefficient beta	Non- standardized regression coefficient B	95% confidence interval of B	*P*-value	Variance inflation factor
Sex (men/women)	0.12	2.73	1.32, 4.15	< 0.001	1.08
Rural/urban region of habitation	0.18	4.31	2.70, 5.91	< 0.001	1.33
Number of days with fruit intake per week	− 0.09	− 0.42	− 0.74, − 0.11	0.009	1.20
Over the past 7 days, how much time did you spend sitting or reclining on a typical day?	0.10	0.001	0.000, 0.001	0.001	1.13
Vigorous activity in leisure time	− 0.09	− 3.17	− 5.41, − 0.94	0.005	1.06
History of headache (no/yes)	0.14	2.80	1.53, 4.07	< 0.001	1.08
Hearing loss score	0.08	0.06	0.02, 0.10	0.009	1.10
Visual acuity (best corrected, binocular) LogMar (logarithm of the minimal angle of resolution)	0.09	1.21	0.34, 2.09	0.007	1.12
Chronic obstructive pulmonary disease (no/yes)	− 0.11	− 4.01	− 6.30, − 1.72	0.001	1.07
Cognitive function score	− 0.28	− 0.45	− 0.55, − 0.34	< 0.001	1.23

For all categorical parameters, the first mentioned gradation is the reference category (i.e., in the case of “men/women”, reference category is “men”; in the case of “no/yes”, reference category is “no”).

Out of the 1491 participants of the UVOS, 14 (0.95%; 95% CI 0.45, 1.43) reported to have attempted suicide, with 3 individuals out of the 14 indicating financial reasons for their attempt. Other reasons were the daughter´s death (n = 1), feeling alone (n = 1). Having thought of suicide within the last 6 months was reported by 7 (0.5%; 95% CI 0.1, 0.8) study participants, and 10 (0.7%; 95% CI 0.3, 1.1) individuals overall declared to have thought of suicide. Overall, 15 (1.0%; 95% CI 0.50, 1.50) individuals attempted or thought of suicide. The overall number of participants having attempted or thought of suicide was too small for a meaningful detailed statistical analysis of associations.

## Discussion

In the UVOS and in the UEMS, a higher depression score was associated with parameters such as female sex, urban region of habitation, lower physical activity, higher hearing loss score, worse visual acuity, and lower cognitive function. In the UVOS and in the UEMS, 15/1491 (1.0%) individuals and 131/5893 (2.2%) had attempted or thought of suicide. These findings agree with observations made in previous studies on other populations and ethnicities. Associations between a higher score of depression or anxiety with female sex, urban region of habitation, and lower physical activity have been found in previous investigations^2,25^. A correlation between depression and pain (in our study headache) have also been reported^26^. In a similar manner, the association between more physical activity and a lower score of depression as detected in our study is in agreement with previous investigations^27,28^. In our study, depression and anxiety score were not correlated with the prevalence of diabetes in the multivariable analysis. It mostly agrees with a recent meta-analysis in which it remained unclear whether and into which direction diabetes was related with depression^29^.

Of interest were the findings that a higher hearing loss score and a worse visual acuity were determinants of higher depression and anxiety, in the old population of the UVOS as well as in the middle-aged study population of the UEMS. It confirms previous studies, such as the investigation conducted by Kim and colleagues, in which the rate of depression was higher in a severe hearing-impaired group of patients as compared to a control group^30–36^. In the National Health and Aging Trends Study, a nationally representative survey in the USA, symptoms of depression and of anxiety were significantly more common in participants with self-reported vision impairment than in those individuals without self-reported vision impairment^36^. In a study from Britain on 13,900 individuals aged 75 + years, visually impaired individuals had a higher prevalence of depression compared with people with good vision. Of visually impaired older people, 13.5% were depressed, while anxiety was not markedly associated with vision impairment^34^.

These findings are paralleled by reports from Kuo and colleagues that the prevalence of hearing and vision impairment was associated with a decreased cognitive function, which was a determinant of a higher depression and anxiety scores in our study population^37^. In a similar manner, a longitudinal study of older US adults from the Health and Retirement Study found higher hazards of incident dementia for individuals with self-reported visual impairment, hearing impairment and dual sensory impairment as combination of both types of impairment compared to individuals without such impairments^38–40^.

Of interest may also be the associations between higher scores of depression or anxiety with a higher prevalence of dry eye disease. It agrees with observations made in previous epidemiologic studies^18,41–43^. In a national veterans population study, Galor and colleagues examined the association between DED and psychiatric disorders and found that depression was associated with a twofold increased risk of having DED^18^. Kim and associates reported in their population-based cross-sectional study a positive correlation between depression and dry eye symptoms^41^. In other smaller scaled case control investigations, the scores of depression and anxiety were associated with DED signs^42^.

From a practical point of view, the relationship between depression or anxiety with vision and hearing impairment and with dry eye disease may perhaps open possibilities for prevention and therapy, since all three conditions can be treated. Future studies may examine whether improvement of vision and hearing impairment may precent the development of depression and anxiety.

The relationship of a lower hand grip force strength with a higher score of depression/anxiety or of having thought of or having attempted suicide agrees with previous reports by Ashdown-Franks and others. It also agrees with general associations of a stronger hand grip force and lower all-cause death, lower cardiovascular death, and lower prevalence of cardiovascular disease^44,45^.

When the results of our studies are discussed, their limitations should be taken into account. First, in both studies, the UEMS and the UVOS, the primary participation rate was around 80%, so that non-participation might have led to a recruitment bias. In particular in the case of the UVOS, one may however take into account, that the high age of the UVOS participants with a minimum of 85 years was associated with a relatively high rate of immobility so that some individuals could not participate in the study. Second, the study area of both studies was typical for Southern Russia with respect to geography, climate and demography. With respect to ethnicity, Russians had a lower percentage in the study samples than in the populations in Central or North-Western Russia. Third, the cross-sectional design of our investigation did not allow to examine longitudinal cause-effect relationships so that we assessed factors associated with, but not risk factors of, the main outcome parameters. Fourth, the univariate analyses included a large list of variables, so that the statistical relevance of the single analysis would have had to be corrected for its dependence of performing a multitude of statistical comparisons. In the multivariable analysis however the inter-dependencies between the independent variables and the correction for the number of statistical tests performed was taken into account. Fifth, It may also be considered that the methods to assess depression and anxiety differ between previous investigations, so that differences in the results between the previous studies and the present investigation have to be discussed with respect to the differences in the techniques applied. Strengths of our project were that the outcome parameters of depression and anxiety have only rarely been examined in Russia, Eastern Europe and Central Asia, the relatively old age of the UVOS population, and the high number of disease and associations addressed in that both the UEMS and the UVOS.

In conclusion, besides female sex, lower level of education and lower cognitive function, it was sensory impairment, namely vision and hearing impairment, which belonged to the determinants of depression and anxiety.

### Supplementary Information

Supplementary Information.

## Data Availability

All identified data are available upon reasonable request from the corresponding author.
