# Supplementary material for: Prevalence of depression, anxiety and suicidal ideas and associated factors, in particular sensory impairments, in a population of Bashkortostan in Russia
Source: Sci Rep. 2023 Oct 12;13:17256. doi: 10.1038/s41598-023-44561-1 (PMC10570299; doi:10.1038/s41598-023-44561-1)
Supplement: Supplementary file 1 — Supplementary Information. [file 41598_2023_44561_MOESM1_ESM.pdf]

Отметить:

☐ **Участник сможет подойти на обследование ____________201_ года (исследуемая группа)**

☐ **Участник не сможет подойти на обследование (группа опроса)**

ID______________Дата______________ Время опроса_____________Время взятия крови___________

ИНФОРМАЦИЯ ДЛЯ ПАЦИЕНТА

Полное название: научно-исследовательский проект "Ural Study on Very Old" (USVO).

Адрес: ГБУ «Уфимский научно-исследовательский институт глазных болезней Академии наук Республики Башкортостан», 450008, Республика Башкортостан, г.Уфа, ул. Пушкина, 90

Номер телефона: 8 (347) 255-30-21

Введение

Вам предложено принять участие в научно-исследовательском проекте «USVO».
Пожалуйста, ознакомьтесь с информацией и задайте любые вопросы, чтобы убедиться, что вы понимаете, с чем для вас будет связано участие в этом исследовании.
Какова цель этого исследования? Цель этого исследования – популяционный анализ, влияние различных факторов на развитие тех или иных заболеваний, изучение анатомо-функциональных особенностей органа зрения среди населения данного региона.

Должен ли я принимать участие в исследовании? Ваше участие в данном исследовании является полностью добровольным. Если вы откажетесь от участия в исследовании или в любое время примете решение о выходе из него это не окажет никакого отрицательного влияния на медицинскую помощь, которую вы получите в будущем. Если в порядок проведения исследования будут внесены изменения, способные повлиять на ваше решение принять в нем участие, вы будете немедленно об этом извещены.

Организатор исследования или регуляторный орган может досрочно прекратить это исследование. Если это произойдет, врач-исследователь немедленно сообщить вам об этом и объяснит причины прекращения исследования. В этом случае регистрация и сбор вашей информации в рамках исследования будут прекращены, но вы будете по-прежнему получать обычную медицинскую помощь в соответствии со стандартом.

Что мне нужно делать? Для участия в этом исследовании, Вас просят дать согласие на сбор и обработку Вашей медицинской информации. Не будет выполнено никаких тестов, не входящих в стандарт помощи пациентам. К числу сведений о вас, собранных в данном исследовании, будут относиться ваш возраст, пол, этническая принадлежность, состояние здоровья, история заболевания, забор биологического материала, глазные тесты и принимаемые вами лекарственные средства. Если вы с этим не согласны, вы не сможете участвовать в данном исследовании. Вас могут попросить заполнять анкету о состоянии вашего зрения и общего состояния здоровья на ежегодной основе.

Вам также будет предложено дать врачу-исследователю и сотрудникам института разрешение обратиться в случае необходимости к любым другим врачам, с которыми вы будете встречаться во время исследования. Такой контакт будет необходим для получения дополнительной медицинской информации, которая будет введена в базу данных исследования.

Какие риски могут быть связаны с участием в этом исследовании? С участием в данном исследовании не связаны иные риски, кроме связанных с получением обычной помощи. Во время участия в данном исследовании вам будет нужно давать необходимую личную информацию, указанную выше. Будут приняты все меры для обеспечения конфиденциальности этой информации.

Каковы мои обязанности во время исследования?

Важно, чтобы вы сообщали вашему врачу-исследователю или другому персоналу исследования обо всех препаратах, которые вы принимаете во время исследования. Это включает рецептурные, безрецептурные препараты и витамины. Также важно, чтобы вы сообщали вашему врачу-исследователю или другому персоналу исследования, если у вас появятся какие-либо необычные симптомы.

Какую пользу можно получить от участия в этом исследовании?

Лично вы можете не получить пользы от участия в исследовании. Однако собранная информация может помочь нам узнать больше о заболеваемости в популяции, влиянии различных факторов на развитие тех или иных заболеваний, анатомо-функциональных особенностей органа зрения среди населения данного региона.

Будет ли конфиденциальным мое участие в данном исследовании?

Будет обеспечена конфиденциальность зарегистрированной у вас информации. Ваши личные контактные данные и медицинские данные, защищенные ключевым кодом ("ключевой код" - это код, который будет вам присвоен во время участия в исследовании; он будет применяться во всех документах данного исследования в качестве идентификатора вместо вашего имени), будут храниться в безопасной базе данных и не будут разглашаться. Врач исследовательского центра будет хранить идентификационный список пациентов исследования на всем протяжении исследования и до 15 лет после его завершения. Любая информация, полученная от других врачей, будет храниться в условиях той же конфиденциальности, что и информация, собранная врачом - исследователем.

Личная информация, полученная от вас в данном исследовании, будет сохранена в базе данных компании ГБУ «Уф НИИ ГБ АН РБ». Данные исследования будут направлены партнерам для контроля и научного анализа. Ваша личная информация будет храниться всегда в соответствии со всеми применимыми законами о защите данных и неприкосновенности личной жизни. Вся информация, имеющая отношение к вашей личности, будет сохранена в условиях конфиденциальности и сообщена только лицам, имеющим на это полномочия.

В случае Вашего согласия участвовать в этом исследовании, органы управления здравоохранением и сотрудники исследовательской группы или этические комитеты, могут получить непосредственный доступ к вашим медицинским документам для контроля процедур исследования или точности данных. Эта информация будет сохранена в условиях конфиденциальности и просмотрена с разрешения вашего лечащего врача.

Документация исследования может быть передана органам управления здравоохранением разных стран мира и направлена за границу для обработки партнерами. Получатели этих документов могут находиться в странах, в том числе Германии, в которых степень защиты информации может отличаться от принятой в стране, где вы получаете лечение. Собранные данные будут объединены с данными, полученными от других участников исследования, и результаты могут быть использованы для научных целей, в том числе исследований или публикаций, либо представлены на конгрессах. Если результаты исследования будут опубликованы в печати или отчете, ваша личность не будет раскрыта.

Получу ли я возмещение в связи с участием в этом исследовании?

Вы не будете получать оплату или возмещение расходов в связи с участием в данном исследовании. Участие в данном исследовании не будет связано для вас с дополнительными расходами.

К кому можно обратиться за дальнейшей информацией?

Данное исследование было подвергнуто контролю и утверждено Независимым междисциплинарным Комитетом по этической экспертизе клинических исследований. Пожалуйста, обращайтесь к врачам - исследователям, если у вас возникнут вопросы об этом исследовании.

Спасибо за интерес к этому исследованию!

Спасибо, что вы нашли время прочесть этот листок информации пациента и прилагаемую форму информированного согласия.

ФОРМА ИНФОРМИРОВАННОГО СОГЛАСИЯ ПАЦИЕНТА

Полное название: научно-исследовательский проект “Ural Study on Very Old” (USVO).

Номер протокола:

Адрес: ГБУ «Уфимский научно-исследовательский институт глазных болезней Академии наук Республики Башкортостан», 450008, Республика Башкортостан, г. Уфа, ул. Пушкина, 90

Номер телефона: 8 (347) 255-30-21

- Я подтверждаю, что мною получены, прочтены и понятны «Информация для пациента и Форма информированного согласия» для данного исследования. У меня было достаточно времени, чтобы ознакомиться с этой информацией, обдумать возможность участия в исследовании и задать вопросы, на которые мною получены удовлетворительные ответы.

- Я знаю, что мое участие в исследовании является добровольным и я могу в любое время выйти из исследования без объяснения причин, что не повлияет на оказываемую мне медицинскую помощь или мои законные права.

- Я знаю и соглашаюсь с тем, что некоторые из моих медицинских документов и данных, собранных во время исследования, могут при необходимости просматриваться лицами – парнерами исследования, их представителями или органами управления здравоохранением.

- Я разрешаю: (1) забор биологического материала; (2) генетические исследования; (3) регистрацию моих закодированных личных данных, в том числе информации о состоянии здоровья; (4) передачу этих данных для научных целей данного исследования (а) партнерам исследования, (б) органам управления здравоохранением и (в) этическим комитетам для просмотра с целью контроля проведения данного исследования в соответствии с применимыми правилами. Я знаю, что степень защиты личных данных в других странах может отличаться от применяемой в моей стране и выражаю согласие с этим.

- Я разрешаю другим врачам, которые могут оказывать мне помощь, передавать врачу-исследователю информацию о состоянии моего здоровья.

Полное имя лечащего врача:

____

- Я добровольно выражаю согласие на участие в данном исследовании.

____________________	__________	__________
ПОЛНОЕ ИМЯ ПАЦИЕНТА<br>*(или законного представителя)*	ДАТА	ПОДПИСЬ
____________________	__________	__________
ПОЛНОЕ ИМЯ ВРАЧА-<br>ИССЛЕДОВАТЕЛЯ,<br>ПОЛУЧИВШЕГО СОГЛАСИЕ<br>*(или назначенного лица)*	ДАТА	ПОДПИСЬ
____________________	__________	__________
ПОЛНОЕ ИМЯ ПАЦИЕНТА<br>*(свидетеля)*	ДАТА	ПОДПИСЬ

“Ural Study on Very Old” (USVO)
Форма I (Сбор исходных данных)
Baseline data collection- Form I

Фамилия Имя сотрудника / Field Worker Name:____
Дата/ Date:____________

Фамилия, имя, отчество пациента:

Name:____

Адрес пациента /
address:____

____

Порядковый номер пациента/ *Identification No*:

A. Пол: Мужской 1 Женский 2

Sex: *Male* *Female*

B. Возраст ____________ лет

Age

C. **Дата рождения:** день/ месяц/ год

Date of Birth: day/month/ year

II. **Отношение к главе семьи** ____

Relationship to the head of the household

III. **Тип семьи:**

Совместная (3 и более поколений) / *Joint*	**1**
Семья в составе родителей и детей до 18лет / *Nuclear*	**2**
Живет один / *lives alone*	**3**
Семья в составе 2 человек / *Family composed of 2 people*	**4**

IV. **Религия** / *Religion:*

Мусульманская / *Muslim*	**1**
Христианская / *Christian*	**2**
Другие, указать / *Other, specify*	**3**

V. **Национальность/**Nationality

**Башкиры/***Bashkir*	**1**
**Русские/***Russian*	**2**
**Татары/***Tatar*	**3**
**Чуваши/***Chuvash*	**4**
**Марийцы/***Mari*	**5**
**Другие/** *Other(**уточнить**)*	**6**

VI. **Форма собственности вашего дома или квартиры** / *House Ownership:*

Собственный дом, квартира/ *Own House*	**1**
Аренда / *Rental*	**2**
Государственное жилье (ипотека) / *On lease*	**3**

VII. **Детали предметов домашнего обихода/** *Household article details***:**

Наименование<br>*name of article*		
Телефон / *Telephone*	1. Да/ Yes	2. Нет/No
Телевизор черно-белый/ Цветной / *T.V- B.W/ Colour*	1. Да/ Yes	2. Нет/No
Велосипед / *Two Wheeler*	1. Да/ Yes	2. Нет/No
Смартфон или планшет / *Smartphone*	1. Да/ Yes	2. Нет/No
Ноутбук или персональный компьютер/ *Laptop or PC*	1. Да/ Yes	2. Нет/No
Легковой автомобиль/ *Car*	1. Да/ Yes	2. Нет/No
Второй дом или квартира/ *Second house*	1. Да/ Yes	2. Нет/No
Другие, указать/ *Other, specify*	1. Да/ Yes	2. Нет/No

VIII. КОДЫ ДЛЯ ДЕМОГРАФИИ: / ***CODES FOR DEMOGRAPHY:***

A. **По отношению к главе** / *Relation to head:*

Глава / Head	**0**
Жена/ *Wife*	**1**
Сын / *Son*	**2**
Дочь / *Daughter*	**3**
Внуки / *Grandchildren*	**4**
Невестка / *Daughter in Law*	**5**
Зять / *Son-in-law*	**6**
Брат / *Brother*	**7**
Сестра / *Sister*	**8**
Жена брата / *Brother's Wife*	**9**
Отец / *Father*	**10**
Мать / *Mother*	**11**
Муж / *Husband*	**12**
Другое / *Other…*	**13**

B. Семейное положение / *Marital Status:*

Женат/замужем / *Married*	**1**
Неженатый/незамужем / *Unmarried*	**2**
Разведен / *Divorced*	**3**
Вдовец/вдова/ Widower/widow	**4**

C. Кровное родство с главой семьи / *Consanguinity*:

да/*yes*	**1**
нет/ *no*	**2**

D. Образование / *Education:*

Неграмотный / *Illiterate*	**1**
5 классов / *Passed Class Vth Pass*	**2**
8 классов / *VIIIth*	**3**
10 классов / *Xth*	**4**
11 классов / *XIth*	**5**
Окончил(а) ВУЗ / *Graduate*	**6**
Аспирант / *Post Graduate*	**7**
Среднее специальное образование (ссуз) / *specialized secondary education*	**8**

E. Занятие/Профессия / *Occupation:*

Собственное сельское хозяйство / *Own Agriculture*	**1**
рабочий сельское хозяйство / *Agriculture coolie*	**2**
Рабочий / *Coolie*	**3**
Государственный служащий / *Government employee*	**4**
Негосударственный работник / *non-government employee*	**5**
Бизнес / *Business*	**6**

F. Ежемесячный доход <u>по мнению пациента</u> / *Monthly Income*

Ниже среднего уровня / *Below Poverty Line*	**1**
Средний уровень /*Average*	**2**
Выше среднего / *Above average*	**3**
Высокий / *High*	**4**

G. Ношение очков / *Wearing Glasses*

да/*yes*	**1**
нет/ *no*	**2**

H. Курение / *Tobacco Consumption*

да/*yes*	**1**
нет/ *no*	**2**

I. Употребление алкоголя / *Alcohol Consumption*

да/*yes*	**1**
нет/ *no*	**2**

J. Наличие глазных заболеваний / *Ocular Disease*

да/*yes*	**1**
нет/ *no*	**2**

“URAL STUDY ON VERY OLD” (USVO)
МЕДИЦИНСКОЕ ОБСЛЕДОВАНИЕ
MEDICAL EXAMINATION

Порядковый номер пациента // *Identification No*

A. **Рост** (в см) / *Height (in cm)* ____

B. **Вес** (в кг) / *Weight (in kg)*_________________(вписывать точное значение, до десятых от кг)

C. **Объем талии** (см) / *Waist circumference (cm)*________________________________

D. **Объем бедер** (см) / *Hips (cm)*____

E. **Артериальное давление** (мм рт.ст.) / *Arterial pressure* (вписывать точное значение)

**Доминирующая рука** / dominant hand	**1.** правая / *right*	**2.** левая / *left*

	Систолическое / *Sist*	Диастолическое / *Diast*
Артериальное давление на **правой руке /** *Arterial pressure on the right hand*		
	Систолическое / *Sist*	Диастолическое / *Diast*
Артериальное давление на **левой руке /** *Arterial pressure on the left hand*		

E1. **<u>Определение АД в области лодыжки</u> /** *ankle pressure measurement*

	Систолическое / *Sist*	Диастолическое / *Diast*
Артериальное давление в области лодыжки на **правой ноге /** *ankle pressure on the right foot*		
	Систолическое / *Sist*	Диастолическое / *Diast*
Артериальное давление в области лодыжки на **левой ноге /** *ankle pressure on the left foot*		

F. Довольны ли вы своим зрением:
How satisfied are you with the quality of your vision:

**1**-Не доволен/ *Dissatisfied*	**2**-Доволен /*Satisfied*	**3**-Затрудняюсь ответить/*Can’t say*

G. Заметили ли Вы изменения в вашем зрении за последние 5 лет?
Have you noticed a change in your vision over the last 5 years?

**1**. Да /*Yes*	**2**. Нет/ *No*	**3**. Затрудняюсь ответить / *Can’t say*

H. Были ли вы на приеме у какого-либо врача?
Have you seen someone about it?

**1.** Да /*Yes*	**2**. Нет /*No*

I. Осматривал ли вас офтальмолог за последние 5 лет?
Have you seen an ophthalmologist in the last 5 years?

**1.** Да /*Yes*	**2**. Нет/*No*

J. Вы когда-нибудь носили солнцезащитные очки?
Have you ever worn sun glasses?

**1.** Да /Yes	**2**. Нет /No

K. Ваши солнечные очки / *Are your sunglasses:*

**1**. Нормальные/ *Normal*
**2**. По назначению/ *Prescription*
**3.** Фотохромные /*Photochromatic*

L. Есть ли в вашей семье наследственные заболевания глаз?
Family history of eye disease in your family:

**1.** Да /*Yes*	**2**. Нет /*No*

Если да, то у кого ___________________, какое заболевание _____________________
If yes then whom *what*

M. Диета /*Diet:*

**1. Вегетарианская**/ *Vegetarian*	**2. Смешанная**/*Mixed*

**M1**	Количество приемов пищи в день.<br>*No. of meals taken in a day.*		
**M2**	Сколько дней в неделю вы едите фрукты?<br>*In a week how many days a week do you eat fruit?*	Дни__________________<br>Days	
**M3**	Сколько фруктов (примерно в граммах) вы едите в один из этих дней?<br>*How many servings of fruit do you eat on one of those days?*	Количество____________<br>*Servings*	
**M4**	Сколько дней в неделю вы едите овощи?<br>*In a week how many days do you eat vegetables?*	Дни__________________<br>Days	
**M5**	Сколько овощей (примерно в граммах) вы едите в один из этих дней?<br>*How many servings of vegetables do you eat on one of those days?*	Количество____________<br>*Servings*	
**M6**	Какой тип масла или жира чаще всего используется для приготовления пищи в Вашей семье?<br>*What type of oil or fat is most often used for meal preparation in your household?*	1.Растительное / *Veg oil*<br>2. Животное / butter<br>3. Ничего / *None in particular*	
**M7**	Употребляете ли Вы продукты, содержащие цельное зерно?<br>*Do you consume foods containing whole grains?*	1. Да / Yes	2. Нет / No
**M8**	Количество соли, употребляемой в сутки? (грамм)<br>*The amount of salt consumed per day? (gram)*		
**M9**	Степень термической обработки мяса, употребляемого в пищу?<br>*The degree of processing of the meat consumed in food?*	1. Слабая / weak<br>2. Средняя / medium<br>3. Сильная / strong	
**M10**	Сколько чашек кофе с кофеином в день вы пьете? *how many cups of coffeinated coffee per day*		
**M11**	Сколько чашек чая в день вы пьете? *how many cups of tea per day*		
**M12**	Какой чай вы предпочитаете? *Which tea do you prefer?*	1 – зеленый / green<br>2 – черный / black<br>3 – одинаково зеленый и черный / equally green and black	

N. ***Курение /*** *Tobacco Consumption:*

Вопросы по курению:

Questions on smoking:

Бросил курить и больше не курю<br>*I quit smoking and do not smoke anymore*	1 - Да *Yes*<br>2 - Нет *No*

-Когда (год)? *When (year)?*___________________________	
Сколько лет вы курили?<br>*How many years have you smoked altogether?*	
Сколько сигарет в день вы курите или курили?<br>*How many cigarettes a day do you smoke or have you smoked?*	
Сколько сигарет в день Вы курили 10-15 лет назад?<br>*How many cigarettes did you smoke 10-15 years ago?*	

Если Вы не курите, перейдите к опроснику О/ If no, go to O

**N1**	Курите ли вы табачные изделия, такие как сигареты, сигары, кальян?/ *Do you currently smoke any tobacco products such as cigarettes, beedi, huka, cigars?*	1. Да/*Yes*	2. Нет/*No*
**N2**	В настоящее время Вы курите каждый день? / *If yes, do you currently smoke tobacco products daily?*	1. Да/*Yes*	2. Нет/*No*
**N3**	Сколько вам было лет, когда вы впервые начали курить? / *How old were you when you first started smoking?*		
**N4**	Вы бросили курить и до сих пор воздерживаетесь? / *Have you stopped smoking & are still completely abstinent?*	1. Да/*Yes*	2. Нет/*No*
Если вы все еще курите, пожалуйста, ответьте на следующие вопросы; *If you still smoke, please answer the following questions about your current smoking behavior;* Если вы успешно бросили курить, пожалуйста, ответьте на следующие вопросы о вашем прошлом отношении к курению. *If you have successfully stopped smoking, please answer the following questions about your past smoking behaviour.*			
**N5**	Вы курите ежедневно? / *Do you smoke daily?*	1. Да/ Yes	2. Нет/No
**N6**	В среднем, сколько сигарет в день? / *On average how many do you smoke each day?*	1.10 и меньше/*10 or less*<br>2. 11-20<br>3. 21-30<br>4. 31 или больше/*31 or more*	
**N7**	Используете ли Вы бездымный табак (нюхательный табак, жевательный табак) / *Do you use smokeless tobacco (such as snuff, chewing tobacco, betel, gutka)?*	1. Да/ Yes	2. Нет/No
**N8**	Если да, то ежедневно ли вы его используете? / *If Yes, do you use smokeless tobacco daily?*	1. Да/ Yes	2. Нет/No
**N9**	В среднем, сколько раз в день? / *On average how many times a day do you use?*		
**N10**	Как скоро после пробуждения вы выкуриваете первую сигарету? / *How soon after you wake up do you smoke your first cigarette?*	1. В течение 5 мин./ *Within 5 min.*<br>2. 6-30мин./*6-30min.*<br>3. 31-60 мин/*31-60 min.*<br>4. Через 60 мин/ *after 60 min.*	
**N11**	Трудно ли вам воздержаться от курения в магазине или автобусе? / *Do you find it difficult to refrain in forbidden places, e.g. places of worship or bus?*	1. Да/ Yes	2. Нет/No
**N12**	Трудно ли вам отказаться от курения?/ *Which cigarette would you hate to give up?* — 1. от первой сигареты утром / *First one in morning*	2. от всех **/** *All others*	3.не трудно / not difficult
**N13**	Вы курите утром после сна больше, чем в течение всего дня? / *Do you smoke more frequently during the first hrs after waking than during the rest of the day?*	1. Да/ Yes	2. Нет /No
**N14**	Курите ли Вы, когда вы больны в дни когда вынуждены оставаться в постели большую часть дня? / *Do you smoke when you are so ill that you must stay in bed most of the day?*	1. Да/ Yes	2. Нет /No

X. Вопросы по кашлю/ *Cough*

**X1**	Сколько раз в день Вы кашляете?/<br>*How many times a day do you cough?*	1. Нет/*No*<br>2.1-3 раза /*1-3 times*<br>3.4-6 раз / *4-6 times*<br>4. больше 6 раз / *more than 6 times*	
**X2**	Как часто у вас наблюдается выделение мокроты с кашлем? /<br>*How often do you have sputum during cough?*	1. Нет/*No*<br>2.1-3 раза /*1-3 times*<br>3.4-6 раз / *4-6 times*<br>4. больше 6 раз / *more than 6 times*	
**X3**	Как часто у вас наблюдается выделение мокроты без кашля? /<br>*How often do you have sputum without cough?*	1. Нет/*No*<br>2.1-3 раза /*1-3 times*<br>3.4-6 раз / *4-6 times*<br>4. больше 6 раз / *more than 6 times*	
**X4**	Как часто простываете зимой?/<br>*How often you do catch cold in winter?*	1. Нет/*No*<br>2.1-3 раза /*1-3 times*<br>3.4-6 раз / *4-6 times*<br>4. больше 6 раз / *more than 6 times*	
**X5**	При подъеме по лестнице затрудняется ли у вас дыхание? /<br>*Is difficult for you to breath when climbing stairs?*	1. Да/ Yes	2. Нет/No
**X6**	При подъеме по лестнице, через сколько шагов затрудняется у вас дыхание?/<br>*After how many steps do you have difficulty in breathing?*	1. 10-20<br>3. 30-40<br>4. больше 50	
**X7**	Есть ли у Вас дома камин или печь с открытым огнем?/<br>*Do you have fireplace or stove with an open fire at home?*	1. Да/ Yes	2. Нет/No
**X8**	Есть ли на Вашем рабочем месте задымленность?/<br>*Is there smokiness at your workplace?*	1. Да/ Yes	2. Нет/No
**X9**	Есть ли на Вашем рабочем месте запыленность?/<br>*Is there dustiness at your workplace?*	1. Да/ Yes	2. Нет/No

O. Ежедневная активность / *Daily Activity:*

**Следующие вопросы о Вашей активности в свободное время. Подумайте о деятельности, которую вы совершаете для восстановления сил, фитнеса или спорта. Не включайте уже упомянутые физические действия, которые Вы делаете на работе или для поездок.**<br>***The next questions ask about activities you do in your leisure time. Think about activities you do for recreation, fitness or sports. Do not include the physical activities you do at work or for travel mentioned already.***			
**O12**	Проходит ли Ваш отдых, спорт или досуг сидя, полулежа или стоя **без** физической нагрузкой более 10 минут в час? /<br>*Does your [recreation, sport or leisure time] involve mostly sitting, reclining or standing, with no physical activity lasting more than 10 minutes at a time?*	1. Да/ Yes	2. Нет /No
**O13**	Ведете ли Вы во время досуга усиленную и активную деятельность (бег или энергичный спорт, тяжелая атлетика) более 10 минут в час? / *In your [leisure time], do you do any vigorous activities like [running or strenuous sports, weight lifting] for at least 10 minutes at a time?*	1. Да/ Yes	2. Нет /No
**O14**	Если да, сколько дней в неделю вы ведете активную деятельность во внерабочее время? / *If Yes, In a typical week, on how many days do you do vigorous activities as part of your [leisure time]?*	Дней в неделю/*Days a week*	
**O15**	Сколько часов, минут у вас это занимает в течение дня?<br>*How much time do you spend doing this on a typical day?*	Часы / *hrs*<br><br>Мин / *min.*	
**O16**	Входит ли в ваше свободное время активность умеренной интенсивности, быстрая ходьба, езда на велосипеде или плавание,	1. Да/ Yes	2. Нет /No

	более 10 минут в час?<br>*In you leisure time, do you do any moderate intensity activities like brisk walking, [cycling or swimming] for at least 10 minutes at a time?*		
**O17**	Сколько дней в неделю Вы ведете активный образ жизни умеренной интенсивности во внерабочее время?<br>*In a typical week, on how many days do you do moderate-intensity activities as part of [leisure time]?*	Количество дней/*Days a week*	
**O18**	Сколько часов, минут в день у вас это занимает?<br>*How much time do you spend doing this on a typical day?*	Часы / *hrs*<br><br>Мин / *min.*	
**Следующий вопрос о положении сидя или полулежа. Вспомните за последнюю неделю, время, проведенное на работе, дома, в отдыхе, в том числе время, проведенное сидя за столом, в гостях у друзей, читая или смотря телевизор, но не включая время, потраченное на сон.**<br>***The following question is about sitting or reclining. Think back over the past 7 days, to time spent at work, at home, in [leisure], including time spent sitting at a desk, visiting friends, reading or watching television, but do not include time spent sleeping.***			
**O19**	За последнюю неделю сколько (часов, минут) вы провели сидя или полулежа? / *Over the past 7 days, how much time did you spend sitting or reclining on a typical day?*	Мин **(x 7 дней)** / *min.*	

P. Алкоголь / *Alchohol*

**P1**	Употребляете ли Вы алкоголь? / *Alchohol Consumed such as Beer, Whisky, Rum, Gin Brandy or Other Local Products*	1. Да/ *Yes*	2.Нет /*No*
**P2**	В каком возрасте Вы впервые употребили алкоголь?<br>*AgeWhen You First Start to Drink Alchohol*		
**P3**	Бросили ли Вы употреблять алкоголь и до сих пор не употребляете? / *Stopped Drinking Alchohol and are Still Completely Abstinent*	1. Да/ *Yes*	2.Нет /*No*
**P4**	В каком возрасте Вы перестали употреблять алкоголь?<br>*Age When Stopped Drinking Alcohol*		
**P5**	Сколько мл алкоголя Вы выпиваете по случаю?<br>*How Many Drinks Containing Alchohol do You Have on a Typical Day When You are Drinkjng*		
**P6**	Как часто вы пьете больше 300 мл крепкого алкогольного напитка по случаю? / *How Often do You Have 6 or More Drinks on One Occasion?*	1-Иногда<br>2-Редко<br>3-Часто<br>4-Незнаю<br>5-Нет	
**P7**	Как часто за последний год вы обнаруживали, что Вы не смогли бросить пить, после того как начали?<br>*How Often During the Last Year have You Found that You Were Not Able to Stop Drinking Once You had Started*	1-Иногда<br>2-Редко<br>3-Часто<br>4-Незнаю<br>5-Нет	
**P8**	Как часто за последний год вы не смогли сделать то, что обычно ожидают от вас из-за выпивки?<br>*How Often During the Last Year have You Failed to do What was Normally Expected from You Because of Drinking*	1-Иногда<br>2-Редко<br>3-Часто<br>4-Незнаю<br>5-Нет	
**P9**	Как часто за последний год вам нужно было выпить утром, чтобы прийти в себя (после тяжелой выпивки)?<br>*How Often During the Last Year have You Needed a First Drink in the Morning to get Yourself Going After a Heavy Drinking*	1-Иногда<br>2-Редко<br>3-Часто<br>4-Незнаю<br>5-Нет	
**P10**	Как часто за последний год вы испытывали чувство вины или угрызения совести после выпивки?<br>*How Often During the Last Year have You had a Feeling of Guilt or Remorse раскаяние after Drinking*	1-Иногда<br>2-Редко<br>3-Часто<br>4-Незнаю<br>5-Нет	
**P11**	Как часто за последний год вы из-за выпивки не могли вспомнить, что случилось в предыдущую ночь?<br>*How Often During the Last Year have You been Unable to*	1-Иногда<br>2-Редко<br>3-Часто	

	Remember What Happened the Night Before You had been	4-Незнаю<br>5-Нет	
**P12**	Были ли Вы или кто-то рядом ранен в результате вашей выпивки? *Have You or Someone Else have been Injured as a Result of Your Drinking*	1. Да/ *Yes*	2.Нет /*No*
**P13**	Были ли обеспокоены тем что вы пьете ваш родственник, друг, врач или другой медицинский работник или предлагали ли вам помощь?<br>*Has a Relative, Friend or a Doctor or Another Health Worker been Concerned About Your Drinking or Suggested You.*	1. Да/ *Yes*	2.Нет /*No*

<u>Вопросы по головной боли в течение года</u> ***(Questions about a headache throughout the year)***

**R1**	Как часто в течение последнего года у вас была головная боль?<br>*How often in your life have you had a headache?*	A.1–4 раз/ times<br>B. 5–9раз/ times<br>C. ≥ 10 раз/ times	
**R2**	По предыдущему вопросу, как часто вы могли бы описать эти моменты головной боли как боль-приступ?<br>*Looking back at the last question, how often would you describe those headache moments as a headache-attack?*	*A.0–4раз/ times*<br>*B. 5–9 раз/ times*<br>*C. ≥ 10 раз/ times*	
**R3**	Сколько дней в месяц у вас головные боли?<br>*How many days per month do you have headaches?*	A.< 1 в месяц/per month<br>B. ≥1 - <15 в месяц/per month<br>C.≥15 в месяц/per month	
**R4**	Как долго длится ваша головная боль, когда вы не принимаете никакие лекарства?<br>*How long does your headache last when you do not take any medication?*	A. 0–30 min<br>B. 30 min–4 h<br>C. 4 h–3 days<br>D. 3–7 days<br>E. >7 days	
**R5**	Каким словом вы бы описали вашу головную боль?<br>*What word would you use to describe your headache?*	A. Пульсирующая<br>Pulsating feeling<br>B. Сжимающая или давящая<br>Tight or pressing feeling<br>C.Жжение или коющая<br>Burning or stabbing feeling<br>D. Другие, такие как<br>Other, such as	
**R6**	Ваша головная боль односторонняя или двухсторонняя?<br>*Is your headache one-sided or two-sided in nature?*	A. односторонняя<br>One-sided<br>B. двухсторонняя<br>Two-sided	
**R7**	Опишите тяжесть головной боли.<br>Describe the severity of your headache.	A.Слабая / Mild<br>B.Средняя / Moderate<br>C.Сильная / Severe<br>D.Очень сильная / Very severe	
Укажите следующие утверждения, если они применимы к вам, когда у вас головная боль.<br>*Indicate by the following statements if these are applicable to you when you have a headache.*			
**R8**	Ежедневные действия (например, подъем по лестнице или пешком) делают мою головную боль сильнее.<br>*Daily activities (such as climbing stairs or walking) make my headache worse.*	1. Да/ *Yes*	2.Нет /*No*
**R9**	Я избегаю повседневной деятельности, когда у меня болит голова.<br>*I avoid daily activities when I have a headache.*	1. Да/ *Yes*	2.Нет /*No*
**R10**	Опишите, что вы испытываете во время головной боли (возможно несколько ответов)<br>*Describe what you experience during your headache (multiple answers possible*)		
	A. Чувствительность к свету<br>Sensitivity to light	1. Да/ *Yes*	2.Нет /*No*

	B. Чувствительность к звуку<br>Sensitivity to sound	1. Да/ *Yes*	2.Нет /*No*
	C. Тошнота и / или рвота<br>Nausea and/or vomiting	1. Да/ *Yes*	2.Нет /*No*
	D. Ни один из вышеперечисленных<br>None of the above	1. Да/ *Yes*	2.Нет /*No*
	E. Другие, такие как<br>Other, such as	1. Да/ *Yes*	2.Нет /*No*

Вопросы по эпилепсии (*Questions about epilepsy)*

**S1**	Были ли у вас когда-либо приступы тряски рук или ног, которые вы не могли контролировать?<br>*Have you ever had attacks of shaking of the arms or legs which you could not control?*	1. Да/ *Yes*	2.Нет /*No*
**S2**	Были ли у вас когда-либо приступы, при которых вы падали и становились бледными?<br>*Have you ever had attacks in which you fall and become pale?*	1. Да/ *Yes*	2.Нет /*No*
**S3**	Вы когда-нибудь теряли сознание?<br>*Have you ever lost consciousness?*	1. Да/ *Yes*	2.Нет /*No*
**S4**	Были ли у вас когда-либо приступы, в которых вы падали с потерей сознания? *Have you ever had attacks in which you fall with loss of consciousness?*	1. Да/ *Yes*	2.Нет /*No*
**S5**	Были ли у вас когда-либо приступы, в которых вы падали и прикусили язык? *Have you ever had attacks in which you fall and bite your tongue?*	1. Да/ *Yes*	2.Нет /*No*
**S6**	Были ли у вас когда-либо приступы, в которых вы падали и теряли контроль над своим мочевым пузырем? *Have you ever had attacks in which you fall and lose control of your bladder?*	1. Да/ *Yes*	2.Нет /*No*
**S7**	Были ли у вас когда-либо короткие приступы дрожания или дрожи в одной руке или ноге или в лице? *Have you ever had brief attacks of shaking or trembling in one arm or leg or in the face?*	1. Да/ *Yes*	2.Нет /*No*
**S8**	Были ли у вас когда-либо приступы, в которых вы теряете контакт с окружающей средой и слышите аномальные запахи? *Have you ever had attacks in which you lose contact with the surroundings and experience abnormal smells?*	1. Да/ *Yes*	2.Нет /*No*
**S9**	Вам когда-либо говорили, что у вас есть эпилепсия или эпилептические припадки? *Have you ever been told that you have or had epilepsy or epileptic fits?*	1. Да/ *Yes*	2.Нет /*No*

Единая шкала оценки болезни Паркинсона (MDS-UPDRS):

1.7 ПРОБЛЕМЫ СО СНОМ<br>На прошлой неделе у вас были проблемы с тем, чтобы уснуть ночью или спать на протяжении всей ночи? Подумайте, насколько вы были отдохнувшим после пробуждения утром.<br>*Over the past week, have you had trouble going to sleep at night or staying asleep through the night? Consider how rested you felt after waking up in the morning.*	0:Нормальный: проблем нет.<br>*Normal: No problems.*<br>1: Незначительный: проблемы со сном присутствуют, но обычно не вызывают проблем, спать всю ночь.<br>*Slight: Sleep problems are present but usually do not cause trouble getting a full night of sleep.*<br>2: Средний: проблемы со сном обычно вызывают некоторые трудности, чтобы спать всю ночь.<br>*Mild: Sleep problems usually cause some difficulties getting a full night of sleep.*<br>3: Умеренный: проблемы со сном вызывают множество трудностей, чтобы спать всю ночь, но я все еще обычно сплю больше половины ночи.<br>*Moderate: Sleep problems cause a lot of difficulties getting a full night of*<br>*sleep, but I still usually sleep for more than half the night.*<br>4: Тяжелый: я обычно не сплю большую часть ночи.<br>*Severe: I usually do not sleep for most of the night.*

1.8 ДНЕВНАЯ СОНЛИВОСТЬ<br>На прошлой неделе у вас были проблемы с бодрствованием в дневное время? *Over the past week, have you had trouble staying awake during the daytime?*	0: Нормальный: нет дневной сонливости.<br>Normal: *No daytime sleepiness.*<br>1: Незначительный: днем возникает сонливость, но я могу справиться, и бодрствовать.<br>*Slight: Daytime sleepiness occurs but I can resist and I stay awake.*<br>2: Средний: Иногда я засыпаю, когда один и расслаблен. Например, во время чтения или просмотра телевизора.<br>*Mild: Sometimes I fall asleep when alone and relaxing. For example, while reading or watching TV.*<br>3: Умеренный: я иногда засыпаю, когда не должен. Например, во время еды или разговаривая с другими людьми.<br>*Moderate: I sometimes fall asleep when I should not. For example, while eating or talking with other people.*<br>4: Тяжелый: я часто засыпаю, когда не должен. Например, во время еды или разговаривая с другими людьми.<br>*Severe: I often fall asleep when I should not. For example, while eating or talking with other people.*
1.9 БОЛЬ И ДРУГИЕ ОЩУЩЕНИЯ<br>На прошлой неделе были ли у вас некомфортные ощущения в вашем теле, такие как боль, покалывающая боль или судороги? *Over the past week, have you had uncomfortable feelings in your body like pain, aches tingling or cramps?*	0: Нормальный: никаких некомфортных ощущений.<br>*Normal: No uncomfortable feelings.*<br>1: Незначительный: у меня есть такие ощущения. Однако я могу заниматься дела и быть с другими люди без затруднений.<br>*Slight: I have these feelings. However, I can do things and be with other people without difficulty.*<br>2: Средний: Эти ощущения вызывают некоторые проблемы, когда я занимаюсь делами и когда нахожусь с другими людьми.<br>*Mild: These feelings cause some problems when I do things or am with other people.*<br>3: Умеренный: Эти ощущения вызывают много проблем, но мне это не мешает заниматься делами или быть с другими людьми.<br>*Moderate: These feelings cause a lot of problems, but they do not stop me from doing things or being with other people.*<br>4: Тяжелый: Эти чувства мешают мне заниматься делами или быть с другими людьми.<br>Severe: *These feelings stop me from doing things or being with other people.*
1.10. ПРОБЛЕМЫ С МОЧЕВОЙ СИСТЕМОЙ.<br>На прошлой неделе у вас были проблемы с контролем мочи? Например, неотложная потребность в мочеиспускании, нужно слишком часто мочиться или случаи недержания?<br>*Over the past week, have you had trouble with urine control? For example, an urgent need to urinate, a need to urinate too often, or urine accidents?*	0: Нормальный: никаких проблем с контролем мочи.<br>*Normal: No urine control problems.*<br>1: Незначительный: мне нужно мочиться часто или срочно. Однако эти проблемы не вызывают затруднений в моей повседневной деятельностьи.<br>*Slight: I need to urinate often or urgently. However, these problems do not cause difficulties with my daily activities.*<br>2: Средний: проблемы с мочой вызывают некоторые трудности в моей повседневной деятельностьи. Однако у меня нет случаев недержания.<br>*Mild: Urine problems cause some difficulties with my daily activities. However, I do not have urine accidents.*<br>3: Умеренный: проблемы с мочой вызывают множество трудностей в моей повседневной

	деятельности, включая случаи недержания.<br>*Moderate: Urine problems cause a lot of difficulties with my daily activities, including urine accidents.*<br>4: Тяжелый: я не могу контролировать мочеиспускание и использую защитную одежду или у меня есть катетер. мочеприемник?<br>*Severe: I cannot control my urine and use a protective garment or have a bladder tube.*
1.11 ПРОБЛЕМЫ КОНСТИПАЦИИ (Запор, задержка стула)<br>За прошедшую неделю у вас были проблемы констипации (запор), которые вызывают трудности опорожнения кишечника?<br>*Over the past week have you had constipation troubles that cause you difficulty moving your bowels?*	0: Нормальный: нет запоров.<br>*Normal: No constipation.*<br>1: Незначительный: есть запор. Я использую дополнительные усилия, чтобы опорожнить кишечник. Однако эта проблема не мешает моей деятельности или моему комфортному существованию.<br>*Slight: I have been constipated. I use extra effort to move my bowels. However, this problem does not disturb my activities or my being comfortable.*<br>2: Средний: запор вызывает некоторые проблемы, мешая моей деятельности или моему комфортному существованию.<br>*Mild: Constipation causes me to have some troubles doing things or being comfortable.*<br>3: Умеренный: запор вызывает у меня много неприятностей, мешая моей деятельности или моему комфортному существованию. Однако это не останавливает меня заниматься чем-либо.<br>Moderate: *Constipation causes me to have a lot of trouble doing things or being comfortable. However, it does not stop me from doing anything.*<br>4: Тяжелый: мне обычно нужна физическая помощь кого-либо, чтобы опорожнить кишечник.<br>*Severe: I usually need physical help from someone else to empty my bowels.*
1.12 ГОЛОВОКРУЖЕНИЕ ПРИ ВСТАВАНИИ<br>За прошедшую неделю вы чувствовали слабость, головокружение или «туманность», когда вы вставали из положения сидя или лежа?<br>*Over the past week, have you felt faint, dizzy or foggy when you stand up after sitting or lying down?*	0: Нормальный: никаких ощущений головокружения или «туманности»/неясности.<br>*Normal: No dizzy or foggy feelings.*<br>1: Незначительный: ощущений головокружения или «туманности» бывают. Однако они не вызывают проблем в моей деятельности. *Slight: Dizzy or foggy feelings occur. However, they do not cause me troubles doing things.*<br>2: Средний: ощущения головокружения или «туманности» заставляют меня держаться за что-либо, но нет необходимости сесть или лечь.<br>*Mild: Dizzy or foggy feelings cause me to hold on to something, but I do not need to sit or lie back down*.<br>3: Умеренный: ощущения головокружения или «туманности» заставляют меня сесть или лечь, чтобы избежать обморока или падения. *Moderate: Dizzy or foggy feelings cause me to sit or lie down to avoid fainting or falling.*<br>4: Тяжелый: ощущения головокружения или «туманности» являются причиной падения или обморока.<br>*Severe: Dizzy or foggy feelings cause me to fall or faint.*
1.13 УСТАЛОСТЬ (ХРОНИЧЕСКАЯ УСТАЛОСТЬ?)<br>На прошлой неделе вы обычно испытывали	0: Нормальный: нет усталости.<br>*Normal: No fatigue.*<br>1: Незначительный: усталость бывает. Однако

усталость? Это ощущение <u>не</u> является ощущением быть сонным или грустным.<br>*Over the past week, have you usually felt fatigued? This feeling is not part of being sleepy or sad.*	это не вызывает у меня проблем в моей деятельности или чтобы находиться с людьми.<br>*Slight: Fatigue occurs. However it does not cause me troubles doing things or being with people.*<br>2: Средний: Усталость вызывает у меня некоторые проблемы заниматься делами или находиться с людьми.<br>*Mild: Fatigue causes me some troubles doing things or being with people.*<br>3: Умеренный: усталость вызывает у меня много проблем заниматься делами или находиться с людьми. Однако это не останавливает меня что-либо делать.<br>Moderate: *Fatigue causes me a lot of troubles doing things or being with people. However, it does not stop me from doing anything.*<br>4: Тяжелый: усталость останавливает меня заниматься делами или находиться с людьми.<br>*Severe: Fatigue stops me from doing things or being with people.*
2.1 РЕЧЬ<br>На прошлой неделе у вас были проблемы с вашей речью?<br>*Over the past week, have you had problems with your speech?*	0: Нормальный: совсем нет (нет проблем).<br>*Normal: Not at all (no problems).*<br>1: Незначительный: Моя речь тихая, невнятная или сбивчивая, но это не вынуждает других людей переспрашивать меня/.<br>*Slight: My speech is soft, slurred or uneven, but it does not cause others to ask me to repeat myself.*<br>2: Средний: Моя речь иногда вынуждает людей просить меня повторить, но не каждый день. *Mild: My speech causes people to ask me to occasionally repeat myself, but not everyday.*<br>3: Умеренный: Моя речь не совсем понятна, и каждый день собеседники просят меня повторить, хотя большая часть моей речи понятна.<br>Moderate: *My speech is unclear enough that others ask me to repeat myself every day even though most of my speech is understood.*<br>4: Тяжелый: большинство или вся моя речь не является понятной.<br>*Severe: Most or all of my speech cannot be understood.*

2.2 СЛЮНА И СЛЮНОТЕЧЕНИЕ (СЛЮНООТДЕЛЕНИЕ)<br>За прошедшую неделю, когда вы просыпались или во время сна у вас было слишком много слюны?<br>*Over the past week, have you usually had too much saliva during when you are awake or when you sleep?*	0: Нормальный: совсем нет (нет проблем).<br>*Normal: Not at all (no problems).*<br>1: Незначительный: у меня много слюны, но не слюнотечение.<br>*Slight: I have too much saliva, but do not drool.*<br>2: Средний: у меня есть слюнотечение во время сна, но нет, когда я просыпаюсь.<br>*Mild: I have some drooling during sleep, but none when I am awake.*<br>3: Умеренный: у меня есть слюнотечение, когда я просыпаюсь, но мне обычно не нужны салфетки или носовой платок.<br>*Moderate: I have some drooling when I am awake, but I usually do not need tissues or a handkerchief.*<br>4: Тяжелый: у меня так много слюнотечения, что я регулярно должен использовать салфетки или носовой платок, чтобы защитить свою одежду.<br>*Severe: I have so much drooling that I regularly need to use tissues or a handkerchief to protect my clothes.*

2.3 ЖЕВАНИЕ И ГЛОТАНИЕ<br>На прошлой неделе у вас обычно возникали проблемы с глотанием таблеток или едой? Вам нужно, чтобы ваши таблетки были измельчены или раздавлены, или ваши блюда необходимо сделать мягкими, нарезать на кусочки или сделать пюре, чтобы не поперхнуться? *Over the past week, have you usually had problems swallowing pills or eating meals? Do you need your pills cut or crushed or your meals to be made soft, chopped or blended to avoid choking?*	0: Нормальный: проблем нет.<br>*Normal: No problems.*<br>1: Незначительный: я знаю о своей медлительности в процессе жевания или о значительных усилиях для проглатывания, но я не поперхнусь или мне не нужно специально подготовливать пищу. *Slight: I am aware of slowness in my chewing or increased effort at swallowing, but I do not choke or need to have my food specially prepared.*<br>2: Средний: мне нужно, чтобы мои таблетки были измельчены или моя пища была специально подготовлена, из-за проблем с жеванием или глотанием, но у меня не было случаев,чтобы я поперхнулся на прошлой неделе.<br>*Mild: I need to have my pills cut or my food specially prepared because of chewing or swallowing problems, but I have not choked over the past week.*<br>3: Умеренный. У меня был случай, когда я поперхнулсяна прошлой неделе. *Moderate. I choked at least once in the past week.*<br>4: Тяжелый: из-за проблем с жеванием и глотанием мне нужен зонд для кормления.<br>*Severe: Because of chewing and swallowing problems, I need a feeding tube.*

Анкета для проверки статуса инсульта
Questionnaire for verifying stroke-free status (QVSFS).

Вам когда-либо говорил ваш доктор о том, что у вас был инсульт?<br>*Were you ever told by a physician that you had a stroke?*	1 -Да Yes<br>2 - Нет No<br>3 – Не известно Unknown
Вам когда-либо говорил ваш доктор о том, что у вас была транзиторная ишемическая атака (ТИА), микроинсульт, преходящее нарушение мозгового кровообращения (ПНМК)?<br>*Were you ever told by a physician that you had a TIA, ministroke, or transient ischemic attack?*	1 -Да Yes<br>2 - Нет No<br>3 – Не известно Unknown
У вас когда-либо была внезапная безболезненная слабость на одной стороне тела?<br>*Have you ever had sudden painless weakness on one side of your body?*	1 -Да Yes<br>2 - Нет No<br>3 – Не известно Unknown
У вас когда-либо было внезапное онемение или ощущение потери чувствительности на одной стороне тела?<br>*Have you ever had sudden numbness or dead feeling on one side of your body?*	1 -Да Yes<br>2 - Нет No<br>3 – Не известно Unknown
У вас когда-либо была внезапная безболезненная потеря зрения в одном или двух глазах?<br>*Have you ever had sudden painless loss of vision in one or both eyes?*	1 -Да Yes<br>2 - Нет No<br>3 – Не известно Unknown
Вы когда-либо внезапно теряли половину вашего зрения?<br>*Have you ever suddenly lost one half of your vision?*	1 -Да Yes<br>2 - Нет No<br>3 – Не известно Unknown

У вас когда-либо была внезапная потеря способности понимать, что люди говорят? *Have you ever suddenly lost the ability to understand what people are saying?*	1 -Да Yes<br><br>2 - Нет No<br><br>3 – Не известно Unknown
У вас когда-либо была внезапная потеря способности выражать себя устно или письменно? *Have you ever suddenly lost the ability to express yourself verbally or in writing?*	1 -Да Yes<br><br>2 - Нет No<br><br>3 – Не известно Unknown

Мичиганский опросник для скрининга диабетической нейропатии
michigan neuropathy screening instrument
(не заполняется если у пациента нет сахарного диабета)
(To be completed by the person with diabetes)

Пожалуйста, уделите несколько минут, чтобы ответить на следующие вопросы о ваших ощущениях в ногах и ступнях. Отметьте да или нет, основываясь на том, как вы обычно себя чувствуете. Спасибо. Please take a few minutes to answer the following questions about the feeling in your legs and feet. Check yes or no based on how you usually feel. Thank you.

1.	Ваши ноги и / или ступни немеют?<br>*Are you legs and/or feet numb?*	1. Да/ *Yes*	2.Нет /*No*
2.	У вас когда-либо была жгучая боль в ногах и / или ступнях?<br>*Do you ever have any burning pain in your legs and/or feet?*	1. Да/ *Yes*	2.Нет /*No*
3.	Ваши ноги слишком чувствительны к прикосновениям?<br>*Are your feet too sensitive to touch?*	1. Да/ *Yes*	2.Нет /*No*
4.	У Вас бывают мышечные судороги в ногах и / или ступнях?<br>*Do you get muscle cramps in your legs and/or feet?*	1. Да/ *Yes*	2.Нет /*No*
5.	Бывают ли у вас какие-либо покалывающие ощущения в ногах или ступнях?<br>*Do you ever have any prickling feelings in your legs or feet?*	1. Да/ *Yes*	2.Нет /*No*
6.	Не больно ли, когда постельные принадлежности (покрывало) касаются вашей кожи?<br>*Does it hurt when the bed covers touch your skin?*	1. Да/ *Yes*	2.Нет /*No*
7.	Когда вы принимаете ванну или душ, можете ли вы сказать горячая вода или холодная вода?<br>*When you get into the tub or shower, are you able to tell the hot water from the cold water?*	1. Да/ *Yes*	2.Нет /*No*
8.	У вас когда-либо была открытая рана на ноге?<br>Have you ever had an open sore on your foot?	1. Да/ *Yes*	2.Нет /*No*
9.	Рассказывал ли ваш врач, что у вас диабетическая невропатия?<br>Has your doctor ever told you that you have diabetic neuropathy?	1. Да/ *Yes*	2.Нет /*No*
10.	Вы чувствуете слабость в течение большей части времени?<br>*Do you feel weak all over most of the time?*	1. Да/ *Yes*	2.Нет /*No*
11.	Ухудшаются ли ваши симптомы ночью?<br>*Are your symptoms worse at night?*	1. Да/ *Yes*	2.Нет /*No*
12.	У вас болят ноги, когда вы ходите пешком?<br>*Do your legs hurt when you walk?*	1. Да/ *Yes*	2.Нет /*No*
13.	Можете ли вы чувствовать свои ступни, когда идете?<br>*Are you able to sense your feet when you walk?*	1. Да/ *Yes*	2.Нет /*No*
14.	Кожа на ногах настолько сухая, что трескается?<br>*Is the skin on your feet so dry that it cracks open?*	1. Да/ *Yes*	2.Нет /*No*
15.	У Вас когда-нибудь была ампутация?<br>*Have you ever had an amputation?*	1. Да/ *Yes*	2.Нет /*No*

Оценка травм (Да /Нет):
Assessment of Injuries (Yes / No):

1. Дорожные травмы *Road Injuries:*

Вы получали травму когда были пешеходом? / *pedestrian*	1. Да/ *Yes*	2.Нет /*No*
Вы получали травму когда были *велосипедистом? / cylist*	1. Да/ *Yes*	2.Нет /*No*
Вы получали травму когда были мотоциклистом? / *motorcyclist*	1. Да/ *Yes*	2.Нет /*No*
Вы получали травму когда были в автомобиле? / *motorvehicle*	1. Да/ *Yes*	2.Нет /*No*

2. Неумышленные травмы *Unintential injuries*

Вы получали травму от падений? / *falls*	1. Да/ *Yes*	2.Нет /*No*
Было ли у Вас утопление? / *drowning*	1. Да/ *Yes*	2.Нет /*No*
Вы получали травму от пожара, жара, горючих веществ? / *fire, heat, hot substances*	1. Да/ *Yes*	2.Нет /*No*
Было ли у Вас отравление? / *poisonings*	1. Да/ *Yes*	2.Нет /*No*
Вы получали травму от взрыва? */ Exposurue to mechanical forces*	1. Да/ *Yes*	2.Нет /*No*
Вы получали травму от поведения животных / *animal conduct*	1. Да/ *Yes*	2.Нет /*No*
Диагностировали (находили) ли у Вас инородное тело легких / *Foreign body lung*	1. Да/ *Yes*	2.Нет /*No*
Диагностировали (находили) ли у Вас инородное тело глаз */ Foreign body eye*	1. Да/ *Yes*	2.Нет /*No*
Диагностировали (находили) ли у Вас инородное тело других частей тела */ Foreign body other body parts*	1. Да/ *Yes*	2.Нет /*No*

3. Самоповреждение/ членовредительство / *Self-harm*

Были ли у Вас случаи самоповреждения или нанесения вреда огнестрельным оружием? */ by fire arm*	1. Да/ *Yes*	2.Нет /*No*
Другим способом? */ by other means*	1. Да/ *Yes*	2.Нет /*No*

4. Межличностное насилие / *Interpersonal violence*

Угрожали ли Вам огнестрельным оружием / *firearm*	1. Да/ *Yes*	2.Нет /*No*
Угрожали ли Вам острым предметом? / *sharp object*	1. Да/ *Yes*	2.Нет /*No*
Подвергались ли Вы сексуальному насилию? / *sexual violence*	1. Да/ *Yes*	2.Нет /*No*
Если да, в каком возрасте ____________________________<br>If yes, at which age?		
Если да, со стороны члена семьи или постороннего/не являющегося членом семьи.<br>If yes, inside of the family or by someone from outside of the family?	1. члена семьи *family*	2. постороннего *outside*
Имелись ли у Вас другие случаи межличностного насилия? / *other*	1. Да/ *Yes*	2.Нет /*No*
Вы получали травму от воздействия сил природы? / *Exposure to forces of nature*	1. Да/ *Yes*	2.Нет /*No*
Вы получали травму от каких либо конфликтов и терроризма? / *Conflict and terrorism*	1. Да/ *Yes*	2.Нет /*No*

Тест опросник по ХОБЛ:

COPD Assessment Test (CAT) (Scores on CAT range from 0–40 with the higher the score, the more severe the disease)

Номер вопроса	Критерии оценки в баллах	Шкала баллов SCORE
1.	Я никогда не кашляю – 0<br>*I never cough*<br>Я постоянно кашляю – 5<br>*I cough all the time*	
2.	У меня в легких совсем нет мокроты (слизи) – 0<br>*I have no phlegm (mucus) in my chest at all*<br>Мои легкие наполнены мокротой (слизью) – 5<br>*My chest is completely full of phlegm (mucus)*	
3.	У меня совсем нет ощущения сдавления в грудной клетке – 0<br>*My chest does not feel tight at all*<br>У меня очень сильное ощущение сдавления в грудной клетке – 5<br>*My chest feels very tight*	
4.	Когда я иду в гору или поднимаюсь вверх на один лестничный пролет, у меня нет одышки – 0<br>When I walk up a hill or one flight of stairs I am not breathless<br>Когда я иду в гору или поднимаюсь вверх на один лестничный пролет, у меня возникает сильная одышка – 5<br>*When I walk up a hill or one flight of stairs I am very breathless*	
5.	Моя повседневная деятельность в пределах дома не ограничена – 0<br>*I am not limited doing any activities at home*<br>Моя повседневная деятельность в пределах дома очень ограничена – 5<br>*I am very limited doing activities at home*	
6.	Несмотря на мое заболевание легких, я чувствую себя уверенно, когда выхожу из дома – 0<br>*I am confident leaving my home despite my lung condition*<br>Из-за моего заболевания легких я совсем не чувствую себя уверенно, когда выхожу из дома – 5<br>*I am not at all confident leaving my home because ofmy lung condition*	
7.	Я сплю очень хорошо – 0<br>I sleep soundly<br>Из-за моего заболевания легких я сплю очень плохо – 5<br>*I don't sleep soundly because of my lung condition*	
8.	У меня много энергии – 0<br>*I have lots of energy*<br>У меня совсем нет энергии – 5<br>*I have no energy at all*	

Шкала дыхания MRC / *MRC shortness of breath scale*:

Ослаблены ли вы при ХОБЛ по шкале<br>Are you *impaired by COPD at:*	1 Только при напряженной деятельности / *Only strenuous activity impaired*<br>2 При энергичной ходьбе / *Vigorous walking impaired*<br>3 При обычной ходьбе / *With normal walking impaired*<br>4 После нескольких минут ходьбы / *After a few minutes of walking impaired*<br>5 При смене одежды/переодевании / *With changing clothing impaired*
Как часто у вас появляюся свистящие хрипы? *How often do you have wheezing?*	0. нет / no<br>1. несколько раз в день / several times per day<br>2. каждый день / every day<br>3. несколько раз в неделю / few times a week<br>4. несколько раз в месяц / several times a month<br>5.несколько раз в год / several times a year

ВОПРОСНИК ДЛЯ ОЦЕНКИ СОСТОЯНИЯ ЗДОРОВЬЯ
HEALTH ASSESSMENT QUESTIONNAIRE (HAQ)

Пожалуйста, выберете один ответ, который лучше всего описывает ваши обычные навыки за прошедшую неделю

Please tick the one response which best describes your usual abilities over the past week **ОДЕВАНИЕ И УХОД ЗА СОБОЙDRESSING AND GROOMING**

Вы можете?<br>Are you able to:	Отметить балл	Отметить самый высокий балл для раздела
Вы можете одеть себя, в том числе завязать шнурки и застегнуть пуговицы?<br>*Dress yourself, including tying shoelaces and doing buttons?*	0 - без затруднения / *without any difficulty*<br>1 - с некоторым затруднением / *with some difficulty*<br>2 - с большим затруднением / *with much difficulty*<br>3 - не могу / *unable to do*	
Мыть ваши волосы?<br>*Shampoo your hair?*	0 - без затруднения / *without any difficulty*<br>1 - с некоторым затруднением / *with some difficulty*<br>2 - с большим затруднением / *with much difficulty*<br>3 - не могу / *unable to do*	

ПОДЪЕМ
RISING

	Отметить балл	Отметить самый высокий балл для раздела
Встать с прямого стула без подлокотников?<br>*Stand up from an armless straight chair?*	0 - без затруднения / *without any difficulty*<br>1 - с некоторым затруднением / *with some difficulty*<br>2 - с большим затруднением / *with much difficulty*<br>3 - не могу / *unable to do*	
Подняться с кровати и лечь в нее?<br>*Get in and out of bed?*	0 - без затруднения / *without any difficulty*<br>1 - с некоторым затруднением / *with some difficulty*<br>2 - с большим затруднением / *with much difficulty*<br>3 - не могу / *unable to do*	

ПРИЕМ ПИЩИ
EATING

Вы можете?<br>Are you able to:	Отметить балл	Отметить самый высокий балл для раздела
Разрезать мясо?<br>*Cut your meat?*	0 - без затруднения / *without any difficulty*<br>1 - с некоторым затруднением / *with some difficulty*<br>2 - с большим затруднением / *with much difficulty*<br>3 - не могу / *unable to do*	
Поднять и поднести полную чашку или стакан ко рту?<br>*Lift a full cup or glass to your mouth?*	0 - без затруднения / *without any difficulty*<br>1 - с некоторым затруднением / *with some difficulty*<br>2 - с большим затруднением / *with much difficulty*<br>3 - не могу / *unable to do*	

Открыть новую коробку с молоком (или стиральным порошком)?<br>*Open a new carton of milk (or soap powder)?*	0 - без затруднения / *without any difficulty*<br>1 - с некоторым затруднением / *with some difficulty*<br>2 - с большим затруднением / *with much difficulty*<br>3 - не могу / *unable to do*	

ПРОГУЛКА
WALKING

Вы можете?<br>Are you able to:	Отметить балл	Отметить самый высокий балл для раздела
Идти по улицепо ровной поверхности?<br>*Walk outdoors on flat ground?*	0 - без затруднения / *without any difficulty*<br>1 - с некоторым затруднением / *with some difficulty*<br>2 - с большим затруднением / *with much difficulty*<br>3 - не могу / *unable to do*	
Подняться на пять ступенек?<br>*Climb up five steps?*	0 - без затруднения / *without any difficulty*<br>1 - с некоторым затруднением / *with some difficulty*<br>2 - с большим затруднением / *with much difficulty*<br>3 - не могу / *unable to do*	

ПОЖАЛУЙСТА, ОТМЕТЬТЕ ЛЮБОЕ СРЕДСТВО ИЛИ УСТРОЙСТВО, КОТОРЫЕ ВЫ ОБЫЧНО ИСПОЛЬЗУЕТЕ ПРИ ЛЮБОМ ИЗ ЭТИХ ДЕЙСТВИЙ:
PLEASE TICK ANY AIDS OR DEVICES THAT YOU USUALLY USE FOR ANY OF THESE ACTIVITIES:

Трость (W) *Cane (W)*	1. Да/ *Yes*	2.Нет /*No*
Костыли (W) *Walking frame(W)*	1. Да/ *Yes*	2.Нет /*No*
Опорные ходунки (W) *Crutches (W)*	1. Да/ *Yes*	2.Нет /*No*
Коляска (W) *Wheelchair (W)*	1. Да/ *Yes*	2.Нет /*No*
Встроенная или специальная посуда? (E) *Built-up or special utensils (E)*	1. Да/ *Yes*	2.Нет /*No*
Специальный или складной стул (A) Special or built-up chair (A)	1. Да/ *Yes*	2.Нет /*No*
Устройства, используемые для одевания (крючки для застегивания пуговиц, натяжения молнии, рожок для обуви) *Devices used for dressing (button hooks, zipper pull, shoe horn)*	1. Да/ *Yes*	2.Нет /*No*

ПОЖАЛУЙСТА, ОТМЕТЬТЕ ЛЮБЫЕ КАТЕГОРИИ, ДЛЯ КОТОРЫХ ВАМ ОБ ЫЧНО ТРЕБУЕТСЯ ПОМОЩЬ ОТ ДРУГОГО ЧЕЛОВЕКА:
PLEASE TICK ANY CATEGORIES FOR WHICH YOU USUALLY NEED HELP FROM ANOTHER PERSON:

Одевание и уход *Dressing and Grooming*	1. Да/ *Yes*	2.Нет /*No*
Подъем *Rising*	1. Да/ *Yes*	2.Нет /*No*
Прием пищи *Eating*	1. Да/ *Yes*	2.Нет /*No*
Прогулка *Walking*	1. Да/ *Yes*	2.Нет /*No*

Пожалуйста, выберете один ответ, который лучше всего описывает ваши обычные навыкиза прошедшую неделю
Please tick the one response which best describes your usual abilities over the past week

Вы можете?<br>Are you able to:	Отметить балл	Отметить самый высокий балл для раздела
Вымыть и вытереть все тело?<br>*Wash and dry your entire body?*	0 - без затруднения / *without any difficulty*<br>1 - с некоторым затруднением / *with some difficulty*<br>2 - с большим затруднением / *with much difficulty*<br>3 - не могу / *unable to do*	
Принимать ванну?<br>*Take a bath?*	0 - без затруднения / *without any difficulty*<br>1 - с некоторым затруднением / *with some difficulty*<br>2 - с большим затруднением / *with much difficulty*<br>3 - не могу / *unable to do*	
Входить и выходить из туалета?<br>*Get on and off the toilet?*	0 - без затруднения / without any difficulty<br>1 - с некоторым затруднением / with some difficulty<br>2 - с большим затруднением / with much difficulty<br>3 - не могу / unable to do	

Досягаемость
REACH

Вы можете?<br>Are you able to:	Отметить балл	Отметить самый высокий балл для раздела
Дотянуться и спустить объект весом 5 фунтов (например, мешок с картофелем) с уровня чуть выше головы<br>*Reach and get down a 5 lb object (e.g. a bag of potatoes) from just above your head?*	0 - без затруднения / *without any difficulty*<br>1 - с некоторым затруднением / *with some difficulty*<br>2 - с большим затруднением / *with much difficulty*<br>3 - не могу / *unable to do*	
Наклониться, чтобы взять одежду с пола?<br>*Bend down to pick up clothing off the floor?*	0 - без затруднения / *without any difficulty*<br>1 - с некоторым затруднением / *with some difficulty*<br>2 - с большим затруднением / *with much difficulty*<br>3 - не могу / *unable to do*	

СХВАТЫВАНИЕ
GRIP

Вы можете?<br>Are you able to:	Отметить балл	Отметить самый высокий балл для раздела
Открывать двери автомобиля?<br>*Open car doors?*	0 - без затруднения / *without any difficulty*<br>1 - с некоторым затруднением / *with some difficulty*<br>2 - с большим затруднением / *with much difficulty*<br>3 - не могу / *unable to do*	
Открывать банки, которые ранее	0 - без затруднения / *without any difficulty*	

были открыты?<br>*Open jars which have been previously opened?*	1 - с некоторым затруднением / *with some difficulty*<br>2 - с большим затруднением / *with much difficulty*<br>3 - не могу / *unable to do*	
Открывать и закрывать (откручивать/закручивать) кран?<br>*Turn taps on and off?*	0 - без затруднения / without any difficulty<br>1 - с некоторым затруднением / with some difficulty<br>2 - с большим затруднением / with much difficulty<br>3 - не могу / unable to do	

АКТИВНЫЕ ДЕЙСТВИЯ
ACTIVITIES

Вы можете?<br>Are you able to:	Отметить балл	Отметить самый высокий балл для раздела
Выполнять поручения и делать покупки?<br>*Run errands and shop?*	0 - без затруднения / *without any difficulty*<br>1 - с некоторым затруднением / *with some difficulty*<br>2 - с большим затруднением / *with much difficulty*<br>3 - не могу / *unable to do*	
Входить и выходить из машины?<br>*Get in and out of a car?*	0 - без затруднения / *without any difficulty*<br>1 - с некоторым затруднением / *with some difficulty*<br>2 - с большим затруднением / *with much difficulty*<br>3 - не могу / *unable to do*	
Выполнять такие повседневные дела, как пылесосить, уборка по дому или легкая работа на огороде<br>*Do chores such as vacuuming, housework or light gardening?*	0 - без затруднения / without any difficulty<br>1 - с некоторым затруднением / with some difficulty<br>2 - с большим затруднением / with much difficulty<br>3 - не могу / unable to do	

ПОЖАЛУЙСТА, ОТМЕТЬТЕ ЛЮБОЕ СРЕДСТВО ИЛИ УСТРОЙСТВО, КОТОРЫЕ ВЫ ОБЫЧНО ИСПОЛЬЗУЕТЕ ПРИ ЛЮБОМ ИЗ ЭТИХ ДЕЙСТВИЙ:
PLEASE TICK ANY AIDS OR DEVICES THAT YOU USUALLY USE FOR ANY OF THESE ACTIVITIES:

Поднятое сиденье для унитаза (H)<br>*Raised toilet seat (H)*	1. Да/ *Yes*	2.Нет /*No*
Сиденье для купания (H)<br>*Bath seat (H)*	1. Да/ *Yes*	2.Нет /*No*
Поручень для ванной (H)<br>Bath rail (H)	1. Да/ *Yes*	2.Нет /*No*
Устройства с длинной рукояткой, чтобы достать что-либо (R)<br>*Long handled appliances for reach (R)*	1. Да/ *Yes*	2.Нет /*No*
Открывашка для банок<br>(для ранее открытых банок) (G)<br>*Jar opener (for jars previously opened) (G)*	1. Да/ *Yes*	2.Нет /*No*
Другое (указать)*Other (specify)*		

ОТМЕТЬТЕ ЛЮБЫЕ КАТЕГОРИИ, ДЛЯ КОТОРЫХ ВАМ ОБЫЧНО ТРЕБУЕТСЯ ПОМОЩЬ ДРУГОГО ЧЕЛОВЕКА:
PLEASE TICK ANY CATEGORIES FOR WHICH YOU USUALLY NEED HELP FROM ANOTHER PERSON:

Гигиена *Hygiene*	1. Да/ *Yes*	2.Нет /*No*

Дотянуться до чего-либо *Reach*	1. Да/ *Yes*	2.Нет /*No*
Схватить и открыть вещь и открывание вещей *Gripping and opening things*	1. Да/ *Yes*	2.Нет /*No*
Поручения и работа по дому<br>*Errands and housework*	1. Да/ *Yes*	2.Нет /*No*

Опросник оценки состояния здоровья из 36-пунктов
36-Item Short Form Survey Instrument (SF-36)

Выберите один вариант для каждого пункта опросника.
Choose one option for each questionnaire item.

1. В целом, вы бы могли сказать, что ваше здоровье:<br>*In general, would you say your health is:*	1 - Отличное *Excellent*<br>2 - Очень хорошее *Very good*<br>3 - Хорошее *Good*<br>4 – Удовлетворительное *Fair*<br>5 – Плохое *Poor*
2. По сравнению с прошлым годом, как бы вы сейчас оценили свое здоровье в целом?<br>*Compared to one year ago, how would you rate your health in general now?*	1 - Гораздо лучше, чем год назад<br>*Much better now than one year ago*<br>2 – Несколько лучше, чем год назад<br>*Somewhat better now than one year ago*<br>3 - Приблизительно так же<br>*About the same*<br>4 - Немного хуже, чем год назад<br>*Somewhat worse now than one year ago*<br>5 - Гораздо хуже, чем год назад<br>*Much worse now than one year ago*

Следующие пункты касаются действий, которые вы можете выполнять в течение своего обычного дня. Ограничивает ли Вас состояние Вашего здоровья сейчас в выполнении этих действий? Если да, то в какой степени?
The following items are about activities you might do during a typical day. Does your health now limit you in these activities? If so, how much?

3. Активная деятельность, такая как бег, поднятие тяжелых предметов, занятие силовыми видами спорта<br>*Vigorous activities, such as running, lifting heavy objects, participating in strenuous sports*	1 - Да, ограничивает значительно<br>*Yes, limited a lot*<br>2 - Да, ограничивает немного<br>*Yes, limited a little*<br>3 - Нет, совсем не ограничивает<br>*No, not limited at all*
4. Умеренная активность, такая как перемещение стола, нажатие пылесоса?<br>*Moderate activities, such as moving a table, pushing a vacuum cleaner, bowling, or playing golf*	1 - Да, ограничивает значительно<br>*Yes, limited a lot*<br>2 - Да, ограничивает немного<br>*Yes, limited a little*<br>3 - Нет, совсем не ограничивает<br>*No, not limited at all*
5. Поднять или нести продукты<br>*Lifting or carrying groceries*	1 - Да, ограничивает значительно<br>*Yes, limited a lot*<br>2 - Да, ограничивает немного<br>*Yes, limited a little*<br>3 - Нет, совсем не ограничивает<br>*No, not limited at all*
6. Подъем на несколько лестничных пролетов<br>*Climbing several flights of stairs*	1 - Да, ограничивает значительно<br>*Yes, limited a lot*<br>2 - Да, ограничивает немного<br>*Yes, limited a little*<br>3 - Нет, совсем не ограничивает<br>*No, not limited at all*
7. Подъем на один лестничный пролет<br>*Climbing one flight of stairs*	1 - Да, ограничивает значительно<br>*Yes, limited a lot*

	2 - Да, ограничивает немного<br>*Yes, limited a little*<br>3 - Нет, совсем не ограничивает<br>*No, not limited at all*
8. Наклониться, становиться на колени или сутулиться<br>*Bending, kneeling, or stooping*	1 - Да, ограничивает значительно<br>*Yes, limited a lot*<br>2 - Да, ограничивает немного<br>*Yes, limited a little*<br>3 - Нет, совсем не ограничивает<br>*No, not limited at all*
9. Прогулка более мили<br>*Walking more than a mile*	1 - Да, ограничивает значительно<br>*Yes, limited a lot*<br>2 - Да, ограничивает немного<br>*Yes, limited a little*<br>3 - Нет, совсем не ограничивает<br>*No, not limited at all*
10. Ходьба пешком несколько кварталов<br>*Walking several blocks*	1 - Да, ограничивает значительно<br>*Yes, limited a lot*<br>2 - Да, ограничивает немного<br>*Yes, limited a little*<br>3 - Нет, совсем не ограничивает<br>*No, not limited at all*
11. Ходьба пешком один квартал<br>*Walking one block*	1 - Да, ограничивает значительно<br>*Yes, limited a lot*<br>2 - Да, ограничивает немного<br>*Yes, limited a little*<br>3 - Нет, совсем не ограничивает<br>*No, not limited at all*
12. Купание или переодевание<br>*Bathing or dressing yourself*	1 - Да, ограничивает значительно<br>*Yes, limited a lot*<br>2 - Да, ограничивает немного<br>*Yes, limited a little*<br>3 - Нет, совсем не ограничивает<br>*No, not limited at all*

В течение последних 4 недель у вас были какие-либо из следующих проблем с вашей работой или другими регулярными ежедневными действиями по причине вашего физического здоровья?
During the past 4 weeks, have you had any of the following problems with your work or other regular daily activities as a result of your physical health?

13. Сократили количество времени, которое вы тратите на работу или другие виды деятельности<br>Cut down the amount of time you spent on work or other activities	1 - Да *Yes*<br>2 - Нет *No*
14. Выполнили меньше, чем хотели бы<br>*Accomplished less than you would like*	1 - Да *Yes*<br>2 - Нет *No*
15. Были ограничены в выполнении какого-либо вида работы или других видов деятельности<br>*Were limited in the kind of work or other activities*	1 - Да *Yes*<br>2 - Нет *No*
16. Имели сложности выполняя работу или другие действия (например, потребовались дополнительные усилия)<br>*Had difficulty performing the work or other activities (for example, it took extra effort)*	1 - Да *Yes*<br>2 - Нет *No*

В течение последних 4 недель у вас были какие-либо из следующих проблем с вашей работой или другими регулярными ежедневными действиями по причине каких-либо эмоциональных проблем (таких как чувство депрессии или беспокойства)?
During the past 4 weeks, have you had any of the following problems with your work or other regular daily activities as a result of any emotional problems (such as feeling depressed or anxious)?

17. Сократили количество времени, затраченного на работу или другие виды деятельности<br>*Cut down the amount of time you spent on work or other activities*	1 - Да *Yes*<br>2 - Нет *No*
18. Выполнили меньше, чем вы хотели бы<br>*Accomplished less than you would like*	1 - Да *Yes*<br>2 - Нет *No*
19. Не выполнили работу или другие действия так же аккуратно, как обычно<br>*Didn't do work or other activities as carefully as usual*	1 - Да *Yes*<br>2 - Нет *No*
20. В течение последних 4 недель, насколько ваши физические или эмоциональные проблемы мешали вашей нормальной социальной деятельности с семьей, друзьями, соседями или группами/в коллективе?<br>*During the past 4 weeks, to what extent has your physical health or emotional problems interfered with your normal social activities with family, friends, neighbors, or groups?*	1 – Совсем не мешали<br>*Not at all*<br>2 – Немного<br>*Slightly*<br>3 – Умеренно<br>*Moderately*<br>4 – Значительно<br>*Quite a bit*<br>5 – Чрезвычайно<br>*Extremely*
21. Насколько сильную физическую боль (интенсивность боли) вы испытывали в последние 4 недели?<br>*How much bodily pain have you had during the past 4 weeks?*	1 – Не было (боли)<br>*None*<br>2 - Очень слабая<br>*Very mild*<br>3 – Слабая<br>*Mild*<br>4 – Умеренная<br>*Moderate*<br>5 - Серьезная<br>*Severe*<br>6 - Очень тяжелая<br>*Very severe*
22. В течение последних 4 недель, насколько боль мешала вашей нормальной работе (включая работу вне дома и домашнюю работу)?<br>*During the past 4 weeks, how much did pain interfere with your normal work (including both work outside the home and housework)?*	1 – Нисколько<br>*Not at all*<br>2 – Немного<br>*Slightly*<br>3 – Умеренно<br>*Moderately*<br>4 – Значительно<br>*Quite a bit*<br>5 – Чрезвычайно<br>*Extremely*

Эти вопросы касаются того, как вы себя чувствовали и каким было Ваше настроение в течение последних 4 недель. По каждому вопросу, пожалуйста, дайте один ответ, который ближе всего к тому, как вы себя чувствовали.

Сколько времени за последние 4 недели ...

These questions are about how you feel and how things have been with you during the past 4 weeks. For each question, please give the one answer that comes closest to the way you have been feeling.

How much of the time during the past 4 weeks...

23. Вы чувствовали себя полными бодрости?<br>*Did you feel full of pep?*	1 - Все время<br>*All of the time*<br>2 - Большую часть времени<br>*Most of the time*<br>3 – Достаточное кол-во времени<br>*A good bit of the time*<br>4 - Некоторое время<br>*Some of the time*<br>5 - Немного времени<br>*A little of the time*<br>6 - Ни разу

	None of the time
24. Вы были очень нервным человеком?<br>*Have you been a very nervous person?*	1 - Все время<br>*All of the time*<br>2 - Большую часть времени<br>*Most of the time*<br>3 – Достаточное кол-во времени<br>*A good bit of the time*<br>4 - Некоторое время<br>*Some of the time*<br>5 - Немного времени<br>*A little of the time*<br>6 - Ни разу<br>*None of the time*
25. Вы чувствовали себя настолько подавленным, что ничто не могло поднять вам настроение?<br>*Have you felt so down in the dumps that nothing could cheer you up?*	1 - Все время<br>*All of the time*<br>2 - Большую часть времени<br>*Most of the time*<br>3 – Достаточное кол-во времени<br>*A good bit of the time*<br>4 - Некоторое время<br>*Some of the time*<br>5 - Немного времени<br>*A little of the time*<br>6 - Ни разу<br>*None of the time*
26. Вы чувствовали себя спокойно и умиротворённо?<br>*Have you felt calm and peaceful?*	1 - Все время<br>*All of the time*<br>2 - Большую часть времени<br>*Most of the time*<br>3 – Достаточное кол-во времени<br>*A good bit of the time*<br>4 - Некоторое время<br>*Some of the time*<br>5 - Немного времени<br>*A little of the time*<br>6 - Ни разу<br>*None of the time*
27. У вас было много энергии?<br>*Did you have a lot of energy?*	1 - Все время<br>*All of the time*<br>2 - Большую часть времени<br>*Most of the time*<br>3 – Достаточное кол-во времени<br>*A good bit of the time*<br>4 - Некоторое время<br>*Some of the time*<br>5 - Немного времени<br>*A little of the time*<br>6 - Ни разу<br>*None of the time*
28. Вы чувствовали себя подавленным и грустным?<br>*Have you felt downhearted and blue?*	1 - Все время<br>*All of the time*<br>2 - Большую часть времени<br>*Most of the time*<br>3 – Достаточное кол-во времени<br>*A good bit of the time*<br>4 - Некоторое время<br>*Some of the time*<br>5 - Немного времени<br>*A little of the time*

	6 - Ни разу<br>*None of the time*
29. Вы чувствовали себя измученным?<br>*Did you feel worn out?*	1 - Все время<br>*All of the time*<br>2 - Большую часть времени<br>*Most of the time*<br>3 – Достаточное кол-во времени<br>*A good bit of the time*<br>4 - Некоторое время<br>*Some of the time*<br>5 - Немного времени<br>*A little of the time*<br>6 - Ни разу<br>*None of the time*
30. Вы были счастливым человеком?<br>*Have you been a happy person?*	1 - Все время<br>*All of the time*<br>2 - Большую часть времени<br>*Most of the time*<br>3 – Достаточное кол-во времени<br>*A good bit of the time*<br>4 - Некоторое время<br>*Some of the time*<br>5 - Немного времени<br>*A little of the time*<br>6 - Ни разу<br>*None of the time*
31. Вы чувствовали себя уставшим?<br>*Did you feel tired?*	1 - Все время<br>*All of the time*<br>2 - Большую часть времени<br>*Most of the time*<br>3 – Достаточное кол-во времени<br>*A good bit of the time*<br>4 - Некоторое время<br>*Some of the time*<br>5 - Немного времени<br>*A little of the time*<br>6 - Ни разу<br>*None of the time*
32. В течение последних 4 недель, как часто ваше физическое здоровье или эмоциональные проблемы мешали вашей социальной деятельности/общаться с людьми (например, навещать друзей, родственников и т. д.)?<br>*During the past 4 weeks, how much of the time has your physical health or emotional problems interfered with your social activities (like visiting with friends, relatives, etc.)?*	1 - Все время<br>*All of the time*<br>2 - Большую часть времени<br>*Most of the time*<br>3 - Некоторое время<br>*Some of the time*<br>4 - Немного времени<br>*A little of the time*<br>5 - Нисколько<br>*None of the time*

Насколько ПРАВДИВЫМ или ЛОЖНЫМ является каждое из следующих утверждений для вас.
How TRUE or FALSE is each of the following statements for you.

33. Кажется, я более склонен к болезням чем другие люди/<br>*I seem to get sick a little easier than other people*	1 - Определенно верно *Definitely true*<br>2 - В основном верно *Mostly true*<br>3 - Не знаю *Don't know*<br>4 - В основном ложные *Mostly false*<br>5 - Определенно ложные *Definitely false*
34. Я так же здоров, как и все, кого я знаю<br>*I am as healthy as anybody I know*	1 - Определенно верно *Definitely true*<br>2 - В основном верно *Mostly true*

	3 - Не знаю *Don't know*<br>4 - В основном ложные *Mostly false*<br>5 - Определенно ложные *Definitely false*
35. Я думаю, что мое здоровье ухудшится<br>*I expect my health to get worse*	1 - Определенно верно *Definitely true*<br>2 - В основном верно *Mostly true*<br>3 - Не знаю *Don't know*<br>4 - В основном ложные *Mostly false*<br>5 - Определенно ложные *Definitely false*
36. У меня отличное здоровье<br>*My health is excellent*	1 - Определенно верно *Definitely true*<br>2 - В основном верно *Mostly true*<br>3 - Не знаю *Don't know*<br>4 - В основном ложные *Mostly false*<br>5 - Определенно ложные *Definitely false*

Опросник состояния зрительных функций
Visual Functioning Questionnaire – 25 (VFQ 25)

ЧАСТЬ 1 - ОБЩЕЕ ЗДОРОВЬЕ И ЗРЕНИЕ
PART 1 - GENERAL HEALTH AND VISION

1. В целом, вы бы сказали, что ваше общее состояние здоровья:<br>*In general, would you say your overall health is:*	1 - Отличное *Excellent*<br>2 - Очень хорошее *Very Good*<br>3 - Хорошее *Good*<br>4 - Удовлетворительное *Fair*<br>5 - Плохое *Poor*
2. В настоящее время вы сказали бы, что ваше зрение обоих глаз (с очками или контактными линзами, если вы их носите) превосходное, хорошее, удовлетворительное, плохое или очень плохое или вы полностью слепы?<br>*At the present time, would you say your eyesight using both eyes (with glasses or contact lenses, if you wear them) is excellent, good, fair, poor, or very poor or are you completely blind?*	1 – Отличное *Excellent*<br>2 – Хорошее *Good*<br>3 – Удовлетворительное *Fair*<br>4 – Плохое *Poor*<br>5 - Очень плохое *Very Poor*<br>6 - Полностью слеп *Completely Blind*
3. Сколько времени выпереживаете о своем зрении?<br>*How much of the time do you worry about your eyesight?*	1 – Никогда *None of the time*<br>2 - Немного времени *A little of the time*<br>3 - Некоторое время *Some of the time*<br>4 - Большую часть времени *Most of the time*<br>5 - Все время? All of the time?
4. Сколько боли или дискомфорта у вас было в глазахи вокруг глаз (например, жжение, зуд или боль)?<br>*How much pain or discomfort have you had in and around your eyes (for example, burning, itching, or aching)? Would you say it is:*	1 - Ни разу *None*<br>2 – Незначительно *Mild*<br>3 – Умеренно *Moderate*<br>4 – Тяжело *Severe*<br>5 - Очень тяжело *Very severe?*

ЧАСТЬ 2 - ТРУДНОСТЬ С ДЕЯТЕЛЬНОСТЬЮ
Следующие вопросы касаются того, насколько Вам сложно, выполнять определенные действия в очках или контактных линзах, если используете их для этой деятельности.
PART 2 - DIFFICULTY WITH ACTIVITIES
The next questions are about how much difficulty, if any, you have doing certain activities wearing your glasses or contact lenses if you use them for that activity.

5. Насколько сложно читать обычный шрифт в газетах? Вы бы сказали, что:<br>*How much difficulty do you have reading ordinary print in newspapers? Would you say you have:*	1 - Нет проблем вообще *No difficulty at all*<br>2 - Немного трудно *A little difficulty*<br>3 - Умеренно сложно *Moderate difficulty*<br>4 - Крайне сложно *Extreme difficulty*<br>5 - Прекратили это из-за вашего зрения *Stopped doing this because of your eyesight*<br>6 - Прекратили это по другим причинам или не заинтересованы в этом *Stopped doing this for*

	other reasons or not interested in doing this
6. Насколько сложно заниматься работой или хобби, которые требуют, чтобы вы хорошо видели вблизи, например, кулинария, шитье, ремонт вещей по дому или использование ручных инструментов? Вы бы сказали: *How much difficulty do you have doing work or hobbies that require you to see well up close, such as cooking, sewing, fixing things around the house, or using hand tools? Would you say:*	1 - Нет проблем вообще *No difficulty at all*<br>2 - Немного трудно *A little difficulty*<br>3 - Умеренно сложно *Moderate difficulty*<br>4 - Крайне сложно *Extreme difficulty*<br>5 - Прекратили это из-за вашего зрения *Stopped doing this because of your eyesight*<br>6 - Прекратили это по другим причинам или не заинтересованы в этом *Stopped doing this for other reasons or not interested in doing this*
7. Из-за вашего зрения, насколько сложно найти что-то на переполненной полке? *Because of your eyesight, how much difficulty do you have finding something on a crowded shelf?*	1 - Нет проблем вообще *No difficulty at all*<br>2 - Немного трудно *A little difficulty*<br>3 - Умеренно сложно *Moderate difficulty*<br>4 - Крайне сложно *Extreme difficulty*<br><br>5 - Прекратили это из-за вашего зрения *Stopped doing this because of your eyesight*<br>6 - Прекратили это по другим причинам или не заинтересованы в этом *Stopped doing this for other reasons or not interested in doing this*
8. Насколько сложно читать надписи на улице или названия магазинов? *How much difficulty do you have reading street signs or the names of stores?*	1 - Нет проблем вообще *No difficulty at all*<br>2 - Немного трудно *A little difficulty*<br>3 - Умеренная сложность *Moderate difficulty*<br>4 - Крайняя сложность *Extreme difficulty*<br>5 - Прекратили это из-за вашего зрения *Stopped doing this because of your eyesight*<br>6 - Прекратили это по другим причинам или не заинтересованы в этом *Stopped doing this for other reasons or not interested in doing this*
9. Из-за вашего зрения, насколько трудно спускаться вниз по ступенькам, по лестнице или с бордюра в тусклом свете или ночью? *Because of your eyesight, how much difficulty do you have going down steps, stairs, or curbs in dim light or at night?*	1 - Нет проблем вообще *No difficulty at all*<br>2 - Немного трудно *A little difficulty*<br>3 - Умеренная сложность *Moderate difficulty*<br>4 - Крайняя сложность *Extreme difficulty*<br>5 - Прекратили это из-за вашего зрения *Stopped doing this because of your eyesight*<br>6 - Прекратили это по другим причинам или не заинтересованы в этом *Stopped doing this for other reasons or not interested in doing this*
10. Из-за вашего зрения, насколько сложно заметить предметы в стороне во время ходьбы? *Because of your eyesight, how much difficulty do you have noticing objects off to the side while you are walking along?*	1 - Нет проблем вообще *No difficulty at all*<br>2 - Немного трудно *A little difficulty*<br>3 - Умеренная сложность *Moderate difficulty*<br>4 - Крайняя сложность *Extreme difficulty*<br>5 - Прекратили это из-за вашего зрения *Stopped doing this because of your eyesight*<br>6 - Прекратили это по другим причинам или не заинтересованы в этом *Stopped doing this for other reasons or not interested in doing this*
11. Из-за вашего зрения, насколько трудно видеть, как люди реагируют на то, что вы говорите? *Because of your eyesight, how much difficulty do you have seeing how people react to things you say?*	1 - Нет проблем вообще *No difficulty at all*<br>2 - Немного трудно *A little difficulty*<br>Умеренная сложность *Moderate difficulty*<br>4 - Крайняя сложность *Extreme difficulty*<br><br>5 - Прекратили это из-за вашего зрения *Stopped doing this because of your eyesight*<br>6 - Прекратили это по другим причинам или не заинтересованы в этом *Stopped doing this for other reasons or not interested in doing this*
12. Из-за вашего зрения, насколько трудно выбрать и подобрать собственную одежду?	1 - Нет проблем вообще *No difficulty at all*<br>2 - Немного трудно *A little difficulty*

Because of your eyesight, how much difficulty do you have picking out and matching your own clothes?	3 Умеренная сложность *Moderate difficulty*<br>4 - Крайняя сложность *Extreme difficulty*<br>5 - Прекратили это из-за вашего зрения *Stopped doing this because of your eyesight*<br>6 - Прекратили это по другим причинам или не заинтересованы в этом *Stopped doing this for other reasons or not interested in doing this*
13. Из-за вашего зрения, насколько сложно встречаться с людьми в их домах, на мероприятиях или в ресторанах?<br>*Because of your eyesight, how much difficulty do you have visiting with people in their homes, at parties, or in restaurants?*	1 - Нет проблем вообще *No difficulty at all*<br>2 - Немного трудно *A little difficulty*<br>Умеренная сложность *Moderate difficulty*<br>4 - Крайняя сложность *Extreme difficulty*<br>5 - Прекратили это из-за вашего зрения *Stopped doing this because of your eyesight*<br>6 - Прекратили это по другим причинам или не заинтересованы в этом *Stopped doing this for other reasons or not interested in doing this*
14. Из-за вашего зрения, насколько сложно ходить в кино, на пьесы или на спортивные мероприятия?<br>*Because of your eyesight, how much difficulty do you have going out to see movies, plays, or sports events?*	1 - Нет проблем вообще *No difficulty at all*<br>2 - Немного трудно *A little difficulty*<br>3 Умеренная сложность *Moderate difficulty*<br>4 - Крайняя сложность *Extreme difficulty*<br>5 - Прекратили это из-за вашего зрения *Stopped doing this because of your eyesight*<br>6 - Прекратили это по другим причинам или не заинтересованы в этом *Stopped doing this for other reasons or not interested in doing this*
15. Вы водите машину в настоящее время, хотя бы время от времени? (Обведите один)<br>*Are you currently driving, at least once in a while? (Circle One)*	1 - Да (Пропустить до Вопроса 15c)<br>*Yes (Skip To Q 15c)*<br>2 - Нет *No*
15a. ЕСЛИ НЕТ: Вы никогда не водили машину или перестали водить машину? (Обведите один)<br>*IF NO: Have you never driven a car or have you given up driving? (Circle One)*	1 - Никогда не водил<br>(Пропустить до Часть 3, Вопрос 17)<br>*Never drove (Skip To Part 3, Q 17)*<br>2 – Бросил перестал *Gave up*
15b. ЕСЛИ ВЫ перестали ВОДИТЬ МАШИНУ: Было ли это главным образом из-за вашего зрения, главным образом по какой-то другой причине или и из-за вашего зрения и других причин? (Обведите один)<br>*IF YOU GAVE UP DRIVING: Was that mainly because of your eyesight, mainly for some other reason, or because of both your eyesight and other reasons? (Circle One)*	1 - В основном зрение<br>(Пропустить до Часть 3, Вопрос 17)<br>*Mainly eyesight (Skip To Part 3, Q 17)*<br>2 - В основном другие причины<br>(Пропустить до Часть 3, Вопрос 17)<br>*Mainly other reasons (Skip To Part 3, Q 17)*<br>3 - И зрение, и другие причины<br>(Пропустить до Часть 3, Вопрос 17)<br>*Both eyesight and other reasons (Skip To Part 3, Q 17)*
15c. ЕСЛИ ВОДИТЕ В НАСТОЯЩЕЕ ВРЕМЯ: Какие трудности вы испытываете, когда ездите днем в знакомых местах? Вы бы сказали, что у вас: (Обведите один)<br>*IF CURRENTLY DRIVING: How much difficulty do you have driving during the daytime in familiar places? Would you say you have: (Circle One)*	1 - Нет проблем вообще *No difficulty at all*<br>2 - Немного трудно *A little difficulty*<br>3 - Умеренная сложность *Moderate difficulty*<br>4 - Крайняя сложность *Extreme difficulty*
16. Насколько сложно вам водить по ночам? Вы бы сказали, что у вас: (Обведите один)<br>*How much difficulty do you have driving at night? Would you say you have: (Circle One)*	1 - Нет проблем вообще *No difficulty at all*<br>2 - Немного трудно *A little difficulty*<br>3 - Умеренная сложность *Moderate difficulty*<br>4 - Крайняя сложность *Extreme difficulty*<br>5 - Прекратили это из-за вашего зрения *Stopped doing this because of your eyesight*<br>6 - Прекратили это по другим причинам или не заинтересованы в этом *Stopped doing this for*

	other reasons or not interested in doing this
16A. С какими трудностями вы сталкиваетесь во время вождения в сложных условиях, например, в плохую погоду, в час пик, на автостраде или в городском движении? Вы бы сказали, что у вас: (Обведите один)<br>*How much difficulty do you have driving in difficult conditions, such as in bad weather, during rush hour, on the freeway, or in city traffic? Would you say you have: (Circle One)*	1 - Нет проблем вообще *No difficulty at all*<br>2 - Немного трудно *A little difficulty*<br>3 - Умеренная сложность *Moderate difficulty*<br>4 - Крайняя сложность *Extreme difficulty*<br>5 - Прекратили это из-за вашего зрения *Stopped doing this because of your eyesight*<br>6 - Прекратили это по другим причинам или не заинтересованы в этом *Stopped doing this for other reasons or not interested in doing this*

ЧАСТЬ 3: ОТВЕТЫ НА ВОПРОСЫ О ПРОБЛЕМАХ СО ЗРЕНИЕМ

Следующие вопросы касаются того, как ваши действия зависят от вашего зрения. Для каждого из них, пожалуйста, обведите число, чтобы указать, какое утверждение является истинным для вас: всё время, большую часть времени, некоторое время, немного времени или никогда.
(Обведите один в каждой линии)

PART 3: RESPONSES TO VISION PROBLEMS

The next questions are about how things you do may be affected by your vision. For each one, please circle the number to indicate whether for you the statement is true for you all, most, some, a little, or none of the time.

ПРОЧИТАЙТЕ КАТЕГОРИИ:<br>*READ CATEGORIES:*	Всё время<br>*All of the time*	Большую часть времени<br>*Most of the time*	Некоторое время<br>*Some of the time*	Немного времени<br>*A little of the time*	Никогда<br>*None of the time*
17. Выполняете ли вы меньше, чем хотели бы из-за вашего зрения?<br>*Do you accomplish less than you would like because of your vision?*	1	2	3	4	5
18. Ограничены ли вы в том, как долго вы можете работать или выполнять другие виды деятельности из-за вашего зрения?<br>*Are you limited in how long you can work or do other activities because of your vision?*	1	2	3	4	5
19. Насколько боль или дискомфорт в глазах или вокруг глаз, например, жжение, зуд или боль, не позволяют вам делать то, что вы хотели бы делать? Вы бы сказали:<br>*How much does pain or discomfort in or around your eyes, for example, burning, itching, or aching, keep you from doing what you'd like to be doing? Would you say:*	1	2	3	4	5

Для каждого из следующих утверждений, пожалуйста, обведите число, чтобы указать, является ли утверждение для вас абсолютной истинной, в основном истинным, или в основном ложно, определенно ложно для вас, или вы не уверены. (Обведите один в каждой линии)

For each of the following statements, please circle the number to indicate whether for you the statement is definitely true, mostly true, mostly false, or definitely false for you or you are not sure. (Circle One On Each Line)

	абсолютная истина *Definitely True*	в основном истина *Mostly True*	не уверен *Not Sure*	в основном ложно *Mostly False*	определенноложно *Definitely False*
20. Я часто остаюсь дома из-за моего зрения *I stay home most of the time*	1	2	3	4	5
21. Я чувствую разочарование большую часть времени из-за моего зрения *I feel frustrated a lot of the time because of my eyesight*	1	2	3	4	5
22. У меня гораздо меньше контроля над тем, что я делаю, из-за моего зрения. *I have much less control over what I do, because of my eyesight.*	1	2	3	4	5
23. Из-за моего зрения, я вынужден слишком полагаться на то, что другие люди говорят мне. *Because of my eyesight, I have to rely too much on what other people tell me.*	1	2	3	4	5
24. Мне необходимо много помощи от других из-за моего зрения *I need a lot of help from others because of my eyesight*	1	2	3	4	5
25. Я переживаю о том, что из-за моего зрения буду делать что то, что смутит меня или других, *I worry about doing things that will embarrass myself or others, because of my eyesight*	1	2	3	4	5

Дополнительные вопросы
ПОДШКАЛА: ОБЩЕЕ ЗДОРОВЬЕ
Appendix of Optional Additional Questions
SUBSCALE: GENERAL HEALTH

A1. Как вы оцениваете свое общее состояние здоровья по шкале, где ноль - настолько плохо, словно смерть, а 10 – наилучшее возможное здоровье? (Обведите один)
How would you rate your overall health, on a scale where zero is as bad as death and 10 is best possible health? (Circle One)

0	1	2	3	4	5	6	7	8	9	10
Наихудший *Worst*										Наилучший *Best*

ПОДШКАЛА: ОБЩЕЕ ЗРЕНИЕ
SUBSCALE: GENERAL VISION

A2. Как бы вы оценили свое зрение сейчас (с очками или контактными линзами, если вы их носите), по шкале от 0 до 10, где ноль означает наихудшее возможное зрение, настолько плохо или хуже, чем быть слепым, а 10 означает наилучшее возможное зрение? (Обведите один)
How would you rate your eyesight now (with glasses or contact lens on, if you wear them), on a scale of from 0 to 10, where zero means the worst possible eyesight, as bad or worse than being blind, and 10 means the best possible eyesight? (Circle One)

0	1	2	3	4	5	6	7	8	9	10
Наихудший *Worst*										Наилучший *Best*

ПОДШКАЛА: ЗРЕНИЕ ВБЛИЗИ
SUBSCALE: NEAR VISION

А3. В очках, насколько трудно вам читать мелкий шрифт в телефонной книге, на упаковке лекарства или в юридических документах? Вы бы сказали: (Обведите один) *Wearing glasses, how much difficulty do you have reading the small print in a telephone book, on a medicine bottle, or on legal forms? Would you say: (Circle One)*	1 - Нет проблем вообще *No difficulty at all*<br>2 - Немного трудно *A little difficulty*<br>3 - Средняя сложность *Moderate difficulty*<br>4 - Крайняя сложность *Extreme difficulty*<br>5 - Прекратили это из-за вашего зрения *Stopped doing this because of your eyesight*<br>6 - Прекратили это по другим причинам или не заинтересованы в этом *Stopped doing this for other reasons or not interested in doing this*
А4. Из-за вашего зрения, насколько сложно понять, точны ли ваши счета, которые вы получаете? (Обведите один)<br>*Because of your eyesight, how much difficulty do you have figuring out whether bills you receive are accurate? (Circle One)*	1 - Нет проблем вообще *No difficulty at all*<br>2 - Немного трудно *A little difficulty*<br>3 - Умеренная сложность *Moderate difficulty*<br>4 - Крайняя сложность *Extreme difficulty*<br>5 - Прекратили это из-за вашего зрения *Stopped doing this because of your eyesight*<br>6 - Прекратили это по другим причинам или не заинтересованы в этом *Stopped doing this for other reasons or not interested in doing this*
А5. Из-за вашего зрения, насколько сложно выполнять такую деятельность, как бритье, укладка волос или макияж? (Обведите один)<br>*Because of your eyesight, how much difficulty do you have doing things like shaving, styling your hair, or putting on makeup? (Circle One)*	1 - Нет проблем вообще *No difficulty at all*<br>2 - Немного трудно *A little difficulty*<br>3 - Умеренная сложность *Moderate difficulty*<br>4 - Крайняя сложность *Extreme difficulty*<br>5 - Прекратили это из-за вашего зрения *Stopped doing this because of your eyesight*<br>6 - Прекратили это по другим причинам или не заинтересованы в этом *Stopped doing this for other reasons or not interested in doing this*

ПОДШКАЛА: ЗРЕНИЕ ВДАЛЬ
SUBSCALE: DISTANCE VISION

А6. Из-за вашего зрения, насколько трудно узнавать людей, которых вы знаете на противоположной стороне комнаты? (Обведите один)<br>*Because of your eyesight, how much difficulty do you have recognizing people you know from across a room? (Circle One)*	1 - Нет проблем вообще *No difficulty at all*<br>2 - Немного трудно *A little difficulty*<br>3 - Средняя сложность *Moderate difficulty*<br>4 - Крайняя сложность *Extreme difficulty*<br>5 - Прекратили это из-за вашего зрения *Stopped doing this because of your eyesight*<br>6 - Прекратили это по другим причинам или не заинтересованы в этом *Stopped doing this for other reasons or not interested in doing this*
А7. Из-за вашего зрения, насколько сложно вам принимать участие в активных видах спорта или других мероприятиях на свежем воздухе, которые вам нравятся (например, бег трусцой или прогулка)? (Обведите один)<br>*Because of your eyesight, how much difficulty do you have taking part in active sports or other outdoor activities that you enjoy (like golf, bowling, jogging, or walking)? (Circle One)*	1 - Нет проблем вообще *No difficulty at all*<br>2 - Немного трудно *A little difficulty*<br>3 - Умеренная сложность *Moderate difficulty*<br>4 - Крайняя сложность *Extreme difficulty*<br>5 - Прекратили это из-за вашего зрения *Stopped doing this because of your eyesight*<br>6 - Прекратили это по другим причинам или не заинтересованы в этом *Stopped doing this for other reasons or not interested in doing this*
Из-за вашего зрения, насколько вам трудно смотреть и наслаждаться программами по	1 - Нет проблем вообще *No difficulty at all*<br>2 - Немного трудно *A little difficulty*

телевизору? (Обведите один)<br>*Because of your eyesight, how much difficulty do you have seeing and enjoying programs on TV? (Circle One)*	3 - Умеренная сложность *Moderate difficulty*<br>4 - Крайняя сложность *Extreme difficulty*<br>5 - Прекратили это из-за вашего зрения *Stopped doing this because of your eyesight*<br>6 - Прекратили это по другим причинам или не заинтересованы в этом *Stopped doing this for other reasons or not interested in doing this*

ПОДШКАЛА: СОЦИАЛЬНАЯ ФУНКЦИЯ
SUBSCALE: SOCIAL FUNCTION

A9. Из-за вашего зрения, насколько вам сложно принимать гостей (друзей и семью в вашем доме)? (Обведите один)<br>*Because of your eyesight, how much difficulty do you have entertaining friends and family in your home? (Circle One)*	1 - Нет проблем вообще *No difficulty at all*<br>2 - Немного трудно *A little difficulty*<br>3 - Средняя сложность *Moderate difficulty*<br>4 - Крайняя сложность *Extreme difficulty*<br>5 - Прекратили это из-за вашего зрения *Stopped doing this because of your eyesight*<br>6 - Прекратили это по другим причинам или не заинтересованы в этом *Stopped doing this for other reasons or not interested in doing this*

ПОДШКАЛА: ОГРАНИЧЕНИЯ ПОВЕДЕНИЯ
SUBSCALE: ROLE LIMITATIONS

A11. Следующие вопросы касаются того, что вы можете делать из-за своего зрения. Для каждого пункта, пожалуйста, обведите число, чтобы указать, что это верно для вас всё время, Большую часть времени , некоторое время, немного времени или никогда. (Обведите один в каждой линии)
The next questions are about things you may do because of your vision. For each item, please circle the number to indicate whether for you this is true for you all, most, some, a little, or none of the time.

	Всё время *All of the time*	Большую часть времени *Most of the time*	Некоторое время *Some of the time*	Немного времени *A little of the time*	Никогда *None of the time*
a. Вы получаете больше помощи от других из-за вашего зрения?<br>*Do you have more help from others because of your vision?*	1	2	3	4	5
b. Вы ограничены в выполнении каких-либо дел, которые вы можете делать из-за вашего зрения?<br>*Are you limited in the kinds of things you can do because of your vision?*	1	2	3	4	5

ПОДШКАЛА: ХОРОШЕЕ САМОЧУВСТВИЕ / СТРЕСС (# A12) и ЗАВИСИМОСТЬ (# A13)
SUBSCALES: WELL-BEING/DISTRESS (#A12) and DEPENDENCY (#A13)

Следующие вопросы касаются того, как вы справляетесь со своим зрением. Для каждого утверждения, пожалуйста, обведите число, чтобы указать, что для вас это определенно верно, в основном верно, в основном ложно или определенно ложно для вас или вы не знаете. (Обведите один в каждой линии)
The next questions are about how you deal with your vision. For each statement, please circle the number to indicate whether for you it is definitely true, mostly true, mostly false, or definitely false for you or you don't know. (Circle One On Each Line)

	определенно верно *Definitely True*	в основном верно *Mostly True*	не уверен *Not Sure*	в основном ложно *Mostly False*	определенно ложно *Definitely False*
A12. Я часто нервничаю из-за моего зрения *I am often irritable because of my eyesight*	1	2	3	4	5
A13. Я не выхожу из дома один, из-за моего зрения *I don't go out of my home alone, because of my eyesight*	1	2	3	4	5

<u>История болезни *medical history:*</u>

**Сколько часов прошло с момента последнего приема пищи?**<br>When the last food and sugar containing drink was taken?	**1** – более 12 часов / fasting more than 12 hours ago<br>**2** – 12-6 часов / hours<br>**3** – 6-3 часа / hours<br>**4** – менее 3 часов / less than 3 hours
Имеется ли у вас группа инвалидности:<br>*Do you have a disability group:*	0-нет / *No*<br>1- 1 группа / *1 group*<br>2- 2 группа / *2 group*<br>3- 3 группа / *3 group*
Как часто вы обращались к врачу за последние 12 месяцев *How often did you go to a doctor in the last 12 months*	
Если обращались, то к какому врачу<br>*If yes, which doctor*	0: не к врачу<br>No doctor<br>1: врач общей медицины / семейный врач<br>*general medicine/family doctor*<br>2: специалист<br>*specialist*
Если к специалисту, то к какому?<br>*Only if a specialist was attended: Which specialist*	Вписать специальность___________________
Вы платили за консультацию?<br>*Have you paid for consultation?*	1. Да / *Yes*<br>2. Нет / *No*
Если да, то сколько вам пришлось заплатить, чтобы обратиться к врачу (общая медицина / семейный врач, специалист) и чтобы получить лечение за последние 12 месяцев?<br>*How much did you have to pay to see a doctor (general medicine/family doctor, specialist) and to get treatment in the last 12 months?*	

01. **Диабет**<br>*Diabetes*	1. Да/ Yes	2. Нет /No	С какого года? / *since*
Тип/*Type*	1. Первый	2. Второй	
Диета/*Diet*	1. Да/ *Yes*	2.Нет /*No*	
Таблетки/ *Tablets*	1. Да/ *Yes*	2.Нет /*No*	
Инсулин/*Insulin*	1. Да/ *Yes*	2.Нет /*No*	

02. **Артериальная гипертония/** *Arterial Hypertension*	1. Да/ *Yes*	2.Нет /*No*	С какого года? / *since*
Применяете ли вы препараты для лечения артериальной гипертонии? / *Do you take tablets orally due to arterial hypertension?*	1. Да/ *Yes*		2. Нет/*No*
Если да, то какие /<br>*if yes, specify?*	1. бета-блокаторы (Анаприлин, Атенолол, Беталок, Конкор) / *beta blockers*<br>2.тиазидные диуретики (Гипотиазид, Навидрекс, Салимид) / *diuretics*<br>3.блокаторы Са каналов (Амлодипин, Верапамил, Дилтиазем, Кордафлекс, Кордипин, Коринфар) / *calcium channel blockers*<br>4.ингибиторы АПФ (Капотен, Каптоприл, Лизиноприл, Энап) / *ACE inhibitors*<br>5.блокаторы рецепторов ангиотензина II (Валсартан, Лозартан, Лозап, Валз) / *ARBs*<br>6. другое (указать) /*other(specify)*_______________________		

**Принимаете ли вы препараты снижения уровня холестерина и липидов в крови?** (аторвастатин, ловастатин, ниацин, фибраты)? / *Do you take tablets orally due to hyperlipidemia?*	1. Да/ *Yes*	2. Нет/*No*

04. **Ангина /** *Angina*	1. Да/ *Yes*	2.Нет /*No*	С какого года? / *since*
05. **Боль в шее /** *Pain in the neck*	1. Да/ *Yes*	2.Нет /*No*	С какого года? / *since*
06. **Боль в грудном отделе позвоночника /** *Pain in the thoracic spine*	1. Да/ *Yes*	2.Нет /*No*	С какого года? / *since*
07. **Боль в пояснице /** *Backache*	1. Да/ *Yes*	2.Нет /*No*	С какого года? / *since*
08. **Головные боли /** *Headache*	1. Да/ *Yes*	2.Нет /*No*	С какого года? / *since*
09.**Заболевания кожи /** *Skin diseases*	1. Да/ *Yes*	2.Нет /*No*	С какого года? / *since*
10. **Железодефицитная анемия /** *Iron-deficiency anemia*	1. Да/ *Yes*	2.Нет /*No*	С какого года? / *since*
11.**Сердечно-сосудистые заболевания /** *Cardiovascular diseases*	1. Да/ *Yes*	2.Нет /*No*	С какого года? / *since*
**Было ли у вас острое нарушение мозгового кровообращения (инсульт)?** *Did you have an acute cerebrovascular accident (stroke)?*	1. Да/ *Yes*	2.Нет /*No*	В каком году? / *since*
Если да, какой / If yes, which	1. ишемический ischemic	2.геморрагический hemorrhagic	
Лечение инсульта: stoke treatment	1.консервативное conservative	2. оперативное surgical	
13. **Старческое слабоумие /** *Dementia*	1. Да/ *Yes*	2.Нет /*No*	С какого года? / *since*
14. **Инфаркт /** *Heart Attack*	1. Да/ *Yes*	2.Нет /*No*	С какого года? / *since*
15. **Потеря сознания /** *Unconscious*	1. Да/ *Yes*	Нет /*No*	С какого года? / *since*
16 **Хроническая диарея**? / *Do you have chronic iarrhea?*	1. Да/ *Yes*	2.Нет /*No*	С какого года? / *since*
17. **Остеоартрит /** *Osteoarthritis*	1. Да/ *Yes*	2.Нет /*No*	С какого года? / *since*
18. **Другие неинфекционные заболевания** *Other non-communicable diseases*	1. Да/ *Yes*	2.Нет /*No*	С какого года? / *since*

19. **Рак** *Cancer*	1. Да/ *Yes*	2.Нет /*No*	С какого года? / *since*
20. **Тип рака** / *Which Cancer*			
Лечение рака / *What treatment:*			
**Операция** *Surgery*	1. Да/ *Yes*	2.Нет /*No*	
**Лучевая терапия** *Radiotherapy*	1. Да/ *Yes*	2.Нет /*No*	
**Химиотерапия** *Chemotherapy*	1. Да/ *Yes*	2.Нет /*No*	

21. **ВОПРОСЫ ДЛЯ ЖЕНЩИН**		
**Менопауза**/ *Menopause*	1. Да/ *Yes*	2.Нет /*No*
**В каком возрасте у Вас было последнее регулярное ежемесячное кровотечение?/**<br>*At what age did you have your last regular menstrual bleeding*?		
**В каком возрасте у Вас было самое последнее ежемесячное кровотечение?/**<br>*At what age did you have your last menstrual bleeding at all?*		
**22. Заболевания щитовидной железы**/ *Thyropathy*	1. Да/ *Yes*	2.Нет /*No*
**Лечение заболеваний щитовидной железы/*Treatment:***		
радиоактивный йод /<br>*Iodine Therapy*	1. Да/ Yes	0. Нет /No
Операция/<br>*Surgery*	2. Да/ Yes	0. Нет /No
принятие тироксина внутрь/<br>*Oral thyroxin*	3. Да/ Yes	0. Нет /No
без лечения/<br>*No treatment*	4. Да/ Yes	0. Нет /No
не могу сказать/<br>*Can't say*	5. Да/ Yes	0. Нет /No

**23.Артрит**/*Arthritis:*	1. Да/ Yes	2. Нет /No	**Какой?/** *Which*

**Принимаете ли вы таблетки внутрь для лечения артрита? /**<br>*Do you take tablets orally due to arthritis?*	1. Да/ *Yes*	2.Нет /*No*
**Принимаете ли вы таблетки внутрь для лечения болей в спине? /**<br>Do you take tablets orally due to back pain?	1. Да/ *Yes*	2.Нет /*No*
**Принимаете ли вы таблетки внутрь для лечения головных болей? /**<br>*Do you take tablets orally due to headache?*	1. Да/ *Yes*	2.Нет /*No*

**24. Падения**/*Falls*		
Вы падали?<br>*Did you fall?*	1. Да/ *Yes*	2.Нет /*No*
Где? /<br>*Where?*		
Когда?/<br>When was it?		
Сколько раз?/<br>How many times?		
Если да, сколько раз после падения у Вас было ухудшение зрения?<br>*If you did, how many times your tumbles were related to bad vision*?		

**25. У вас был перелом?**<br>Did you have a fracture?<br><br>Если да то перелом какой кости у вас был?/<br>*If yes what bone has been fractured?*<br><br>____	1. Да/ *Yes*	2.Нет /*No*

**26. Страдаете ли Вы другими заболеваниями, или переносили операции?**<br>*Did you suffer from other diseases or undertake any operations?*<br><br>____	1. Да/ *Yes*	2.Нет /*No*

**27. Попадали ли вы в больницу из-за снижения артериального давления, потери крови или шока?** /<br>*Were you in hospital due to blood pressure falling down or blood loss or shock?*	1. Да/Yes	2. Нет /No	3.Затрудняюсь ответить / Can't say
**28. Другие неумышленные раны** /<br>*Other unintentional injuries*		1. Да/ *Yes*	2.Нет /*No*

29. **Хроническая обструктивная болезнь легких** /<br>*Chronic obstructive pulmonary disease*	1. Да/ *Yes*	2.Нет /*No*	С какого года? / *since*

Лечение ХОБЛ: ***Treatment of COPD:***

Ингаляционные ГКС (Пульмикорт, Беклазон) / *Inhalation GCS*	1. Да/ Yes	2. Нет /No
Бронходилататоры (Спирива, альбутамол, Беротек) / *Bronchodilator*	1. Да/ Yes	2. Нет /No
Ингибиторы фосфодиэстеразы-4 (рофлумиласт, циломиласт) / *Phosphodiesterase-4 Inhibitors*	1. Да/ Yes	2. Нет /No
Комбинированные препараты(Аноро Эллипта) / *Combined medicaments*	1. Да/ Yes	2. Нет /No
Метилксантины (Эуфиллин) / *Methylxanthines*	1. Да/ Yes	2. Нет /No
Другое, указать / *Other, specify*_________________________		

30. **Астма /**<br>*Asthma*	1. Да/ *Yes*	2.Нет /*No*	С какого года? / *since*

Лечение астмы:

Ингаляционные ГКС (Пульмикорт, Беклазон) / *Inhalation GCS*	1. Да/ Yes	2. Нет /No
Системные ГКС (Дексаметазон, Метилпреднизолон) / *System GCS*	1. Да/ Yes	2. Нет /No
β2-агонисты короткого действия (фенотерол, сальбутамол и тербуталин) / *short-acting β2-agonists*	1. Да/ Yes	2. Нет /No
Комбинированные препараты (Симбикорт, Серетид)/ *Combined medicaments*	1. Да/ Yes	2. Нет /No
Другое, указать / *Other, specify*_________________________		

31. Болезни почек:

Есть ли у Вас хронические заболевания почек / *Chronic kidney disease*	1. Да/ *Yes*	2.Нет /*No*
Если да, из за диабета? / *due to diabetes*	1. Да/ *Yes*	2.Нет /*No*
Если да, из за артериальной гипертензии? / *due to hypertension*	1. Да/ *Yes*	2.Нет /*No*
Если да, из за гломерулонефрита? / *due to glomerulonephritis*	1. Да/ *Yes*	2.Нет /*No*

32. Заболевания суставов
Diseases of the joints

Испытываете ли Вы боль в суставах после физической нагрузки, долгой ходьбы, физической работы?<br>*Do you suffer from joint pain after physical exertion, long walking, physical work?*	1. Очень часто *Very often*<br>2. Иногда / *Sometimes*<br>3. Крайне редко / *Extremely rare*
В каких суставах Вы чаще всего ощущаете боль?<br>*In which joints do you most often feel pain?*	1. В каждом суставе и позвоночнике / *In every joint and spine*<br>2. В фалангах пальцев, запястьев, иногда в коленных суставах / *In phalanges of fingers, wrists, sometimes in knee joints*<br>3. Иногда болят ступни ног / *Sometimes feet are aching*
Испытываете ли Вы боль в суставах при подъеме или спуске по лестнице?<br>*Do you suffer from joint pain while climbing or descending the stairs?*	1. Да / *Yes*<br>2. Нет / *No*
Если да, то в каких суставах? *If so, in which joints?*________________________	
Испытываете ли Вы боль в суставах в ночное время?<br>*Do you experience joint pain at night?*	1. Да / *Yes*<br>2. Нет / *No*
Если да, то в каких суставах? *If so, in which joints?*____________________	
Бывает ли у Вас скованность по утрам?<br>*Do you have stiffness in the morning?*	1. Да / *Yes*<br>2. Нет / *No*
Если да, то в каких суставах? *If so, in which joints?*________________________	
Снимают ли боль в суставах физические упражнения?<br>Does physical exercise relieve pain in joints?	1. Да / *Yes*<br>2. Нет / *No*
Обращались ли Вы когда-нибудь к врачу-ортопеду (ревматологу)? / *Have you ever been to orthopedist (rheumatologist)?*	1. Никогда / Never<br>2. Была, несколько лет назад / I have been a few years ago<br>3. Посещаю один раз в год / I visit once a year<br>4. Посещаю регулярно / I visit

33. **Урологические расстройства** *Urologic disorders*

Вопрос для мужчин! У вас есть трудности с удержанием мочи или случается ли, что вы теряете мочу непреднамеренно? *Do you have difficulties in keeping the urine or does it occur that you loose urine unvoluntarily?*	1 - Да *Yes*<br>2 - Нет *No*
Если да, то<br>*If yes, does it occur*	1 - это случается один раз в день, когда нижнее белье слегка мокрое в течение короткого времени<br>*once per day with the underpant slightly wet for short time*<br>2 - дважды или чаще в день или хотели бы сменить нижнее белье<br>*twice or more often per day or would then like to change the underpants*<br>3 – носите подгузники или другие гигиенические средства<br>*do you were pampers*

34. Грыжа *Hernia:*

У вас есть грыжа? / *Do you have hernia*	1. Да/ *Yes*	2.Нет /*No*
Если да, паховая? / *inguinal*	1. Да/ *Yes*	2.Нет /*No*
Если да, бедренная? / *femoral*	1. Да/ *Yes*	2.Нет /*No*
Если да, брюшной полости? / *abdominal*	1. Да/ *Yes*	2.Нет /*No*

35. Какие заболевания у вас есть, как сказал вам ваш врач? *Which do diseases do you have as told by your doctor?*

____

____

Потеря слуха/ Hearing loss

**Вопрос/** *Question*	**Варианты ответа/** *response options*	Баллы/ *Scores*
**Отмечаете ли Вы снижение слуха?/** *Have you noticed the hearing loss?*	**Да/** *Yes*<br>**Нет**/*No*<br>**Иногда/** *Sometimes*	4<br>0<br>2
**Заставляют ли проблемы со слухом чувствовать себя смущенным при знакомстве с новыми людьми?/** *Does a hearing problem cause you to feel embarrassed when meeting new people?*	**Да/** *Yes*<br>**Нет**/*No*<br>**Иногда/** *Sometimes*	4<br>0<br>2
**Заставляют ли проблемы со слухом чувствовать себя непонятым при разговоре с членами Вашей семьи?/** *Does a hearing problem cause you to feel frustrated when talking to members of your family?*	**Да/** *Yes*<br>**Нет**/*No*<br>**Иногда/** *Sometimes*	4<br>0<br>2
**Испытываете ли Вы затруднения, когда кто-то говорит шепотом?/** *Do you have difficulty hearing when someone speaks in a whisper?*	**Да/** *Yes*<br>**Нет**/*No*<br>**Иногда/** *Sometimes*	4<br>0<br>2
**Вы чувствуете себя помехой из-за проблем со слухом?/** *Do you feel handicapped by a hearing problem?*	**Да/** *Yes*<br>**Нет**/*No*<br>**Иногда/** *Sometimes*	4<br>0<br>2
**Вызывают ли проблемы со слухом трудности при посещении друзей, родственников или соседей?/** *Does a hearing problem cause you difficulty when visiting friends, relatives, or neighbors?*	**Да/** *Yes*<br>**Нет**/*No*<br>**Иногда/** *Sometimes*	4<br>0<br>2
**Вызывают ли проблемы со слухом трудности для посещения религиозных мест менее часто, чем Вы бы этого хотели?/** *Does a hearing problem cause you to attend religious services less often than you would like?*	**Да/** *Yes*<br>**Нет**/*No*<br>**Иногда/** *Sometimes*	4<br>0<br>2
**Вызывают ли проблемы со слухом споры с членами семьи?/** *Does a hearing problem cause you to have arguments with family members?*	**Да/** *Yes*<br>**Нет**/*No*<br>**Иногда/** *Sometimes*	4<br>0<br>2
**Вызывают ли проблемы со слухом трудности при послушивании ТВ или радио?/** *Does a hearing problem cause you difficulty when listening to TV or radio?*	**Да/** *Yes*<br>**Нет**/*No*<br>**Иногда/** *Sometimes*	4<br>0<br>2
**Чувствуете ли вы что трудности с возможными пределами вашего слуха препятствует Вашей личной или общественной жизни?/** *Do you feel that any difficulty with your hearing limits or hampers your personal or social life?*	**Да/** *Yes*<br>**Нет**/*No*<br>**Иногда/** *Sometimes*	4<br>0<br>2
**Вызывают ли проблемы со слухом трудности, когда вы в ресторане с родственниками или друзьями?/** *Does a hearing problem cause you difficulty when in a restaurant with relatives or friends?*	**Да/** *Yes*<br>**Нет**/*No*<br>**Иногда/** *Sometimes*	4<br>0<br>2

ПИТТСБУРГСКИЙ ОПРОСНИК НА ОПРЕДЕЛЕНИЕ ИНДЕКСА КАЧЕСТВА СНА (PSQI)
Pittsburgh Sleep Quality Index (PSQI)

Инструкции: Следующие вопросы касаются Вашего сна в течение прошедшего месяца. Ваши ответы должны отражать наиболее подходящую ситуацию для большинства дней и ночей за прошедший месяц. Пожалуйста, ответьте на все вопросы.
Instructions: The following questions relate to your usual sleep habits during the past month only. Your answers should indicate the most accurate reply for the majority of days and nights in the past month. Please answer all questions.

1. **В какое время Вы обычно ложились спать в течение последнего месяца?** / *During the past month, what time have you usually gone to bed at night?*
ОБЫЧНОЕ ВРЕМЯ ОТХОДА К НОЧНОМУ СНУ (отметить час от 0 до 23) _______

2. **Сколько времени (минут) Вам обычно требовалось, чтобы заснуть (в течение последнего месяца)?** / *During the past month, how long (in minutes) has it usually taken you to fall asleep each night?*

КОЛИЧЕСТВО МИНУТ	0 -<15 минут / minutes<br>1 - 16-30 минут / minutes<br>2 –31-60 минут / minutes<br>3 -> 60 минут / minutes

3. **В какое время Вы обычно просыпались в течение последнего месяца?** / *During the past month, what time have you usually gotten up in the morning?*
ОБЫЧНОЕ ВРЕМЯ ПОДЪЕМА УТРОМ (отметить час от 0 до 23) _______

4. **Сколько часов Вы спали за ночь в течение последнего месяца?** (количество часов может отличаться от количества времени, проведенного в постели) / *During the past month, how many hours of actual sleep did you get at night? (This may be different than the number of hours you spent in bed)*

КОЛИЧЕСТВО ЧАСОВ СНА ЗА НОЧЬ	0 - > 7 часов / hours<br>1 - 6-7 часов / hours<br>2 - 5-6 часов / hours<br>3 - <5 часов / hours

Инструкции: Для каждого из оставшихся вопросов выберите один наиболее подходящий ответ. Пожалуйста, ответьте на все вопросы.

5. **В течение прошедшего месяца как часто у Вас были проблемы со сном, потому что Вы…** / *During the past month, how often have you had trouble sleeping because you…*

(a) не могли уснуть в течение 30 минут / *Cannot get to sleep within 30 minutes*	*0.* Ни разу в течение последнего месяца / *Not during the past month*<br>*1.* Менее, чем один раз в неделю / *Less than once a week*<br>*2.* Один или два раза в неделю / Once or twice a week<br>3. Три или более раз в неделю / *Three or more times a week*
(b) просыпались в середине ночи или под утро / *Wake up in the middle of the night or early morning*	*0.* Ни разу в течение последнего месяца / *Not during the past month*<br>*1.* Менее, чем один раз в неделю / *Less than once a week*<br>*2.* Один или два раза в неделю / Once or twice a week<br>3. Три или более раз в неделю / *Three or more times a week*
(c) были вынуждены вставать, чтобы воспользоваться ванной комнатой / *Have to get up to use the bathroom*	*0.* Ни разу в течение последнего месяца / *Not during the past month*<br>*1.* Менее, чем один раз в неделю / *Less than once a week*<br>*2.* Один или два раза в неделю / Once or twice a week<br>3. Три или более раз в неделю / *Three or more times a week*
(d) не могли свободно дышать / *Cannot breathe comfortably*	*0.* Ни разу в течение последнего месяца / *Not during the past month*<br>*1.* Менее, чем один раз в неделю / *Less than once a week*<br>*2.* Один или два раза в неделю / Once or twice a week<br>3. Три или более раз в неделю / *Three or more times a week*
(e) кашляли или громко храпели / *Cough or snore loudly*	*0.* Ни разу в течение последнего месяца / *Not during the past month*<br>*1.* Менее, чем один раз в неделю / *Less than once a week*

	2. Один или два раза в неделю / Once or twice a week<br>3. Три или более раз в неделю / *Three or more times a week*
(f) чувствовали, что слишком холодно / Feel too cold	*0.* Ни разу в течение последнего месяца / *Not during the past month*<br>*1.* Менее, чем один раз в неделю / *Less than once a week*<br>*2.* Один или два раза в неделю / Once or twice a week<br>3. Три или более раз в неделю / *Three or more times a week*
(g) чувствовали, что слишком жарко / Feel too hot	*0.* Ни разу в течение последнего месяца / *Not during the past month*<br>*1.* Менее, чем один раз в неделю / *Less than once a week*<br>*2.* Один или два раза в неделю / Once or twice a week<br>3. Три или более раз в неделю / *Three or more times a week*
(h) видели плохие сны / *Have bad dreams*	*0.* Ни разу в течение последнего месяца / *Not during the past month*<br>*1.* Менее, чем один раз в неделю / *Less than once a week*<br>*2.* Один или два раза в неделю / Once or twice a week<br>3. Три или более раз в неделю / *Three or more times a week*
(i) испытывали боль / *Have pain*	*0.* Ни разу в течение последнего месяца / *Not during the past month*<br>*1.* Менее, чем один раз в неделю / *Less than once a week*<br>*2.* Один или два раза в неделю / Once or twice a week<br>3. Три или более раз в неделю / *Three or more times a week*
(j) Другая(ие) причина(ы), пожалуйста, напишите / Other reason(s), please describe	
Как часто за прошедший месяц у Вас были проблемы со сном по этой причине? / *How often during the past month have you had trouble sleeping because of this?*	*0.* Ни разу в течение последнего месяца / *Not during the past month*<br>*1.* Менее, чем один раз в неделю / *Less than once a week*<br>*2.* Один или два раза в неделю / Once or twice a week<br>3. Три или более раз в неделю / *Three or more times a week*
6. **Как бы Вы охарактеризовали качество Вашего сна за последний месяц?** / *During the past month, how would you rate your sleep quality overall?*	*0.* Очень хорошее / *Very good*<br>*1.* Достаточно хорошее / *Fairly good*<br>*2.* Скорее плохое / *Fairly bad*<br>3. Очень плохое / *Very bad*
7. **За прошедший месяц как часто Вы принимали лекарства (прописанные или «безрецептурные»), которые помогают уснуть?** / *During the past month, how often have you taken medicine to help you sleep (prescribed or “over the counter”)*	*0.* Ни разу в течение последнего месяца / *Not during the past month*<br>*1.* Менее, чем один раз в неделю / *Less than once a week*<br>*2.* Один или два раза в неделю / Once or twice a week<br>3. Три или более раз в неделю / *Three or more times a week*
8. **Как часто за прошедший месяц Вам было сложно оставаться бодрствующим во время вождения автомобиля, после приема пищи или в процессе социальной деятельности?** / *During the past month, how often have you had trouble staying awake while driving, eating meals, or engaging in social activity?*	*0.* Ни разу в течение последнего месяца / *Not during the past month*<br>*1.* Менее, чем один раз в неделю / *Less than once a week*<br>*2.* Один или два раза в неделю / Once or twice a week<br>3. Три или более раз в неделю / *Three or more times a week*
9. **За прошедший месяц насколько сложно было Вам сохранять достаточный настрой на то, чтобы сделать все дела?** / *During the past month, how much of a problem has it been for you to keep up enough enthusiasm to get things done?*	0. Совсем не сложно / *No problem at all*<br>1. Лишь чуть-чуть сложно / *Only a very slight problem*<br>2. Несколько сложно / *Somewhat of a problem*<br>3. Очень сложно / *A very big problem*

10. **Есть ли у Вас партнер, с которым делите постель, или**	0. Нет партнера или соседа по комнате / *No bed partner or room mate*

**сосед по комнате?**<br>*Do you have a bed partner or room mate?*	1. Партнер или сосед в другой комнате / *Partner/room mate in other room*<br>2. Партнер в этой же комнате, но в другой постели / *Partner in same room but not same bed*<br>3. Делим одну постель (с партнером) / Partner in same bed

Если у Вас есть партнер или сосед по комнате, спросите его/ее, как часто за прошедший месяц у Вас были… / If you have a room mate or bed partner, ask him/her how often in the past month you have had:

(a) Громкий храп / *Loud snoring*	*0.* Ни разу в течение последнего месяца / *Not during the past month*<br>*1.* Менее, чем один раз в неделю / *Less than once a week*<br>*2.* Один или два раза в неделю / Once or twice a week<br>3. Три или более раз в неделю / *Three or more times a week*
(b) Длительные задержки дыхания во время сна / *Long pauses between breaths while asleep*	*0.* Ни разу в течение последнего месяца / *Not during the past month*<br>*1.* Менее, чем один раз в неделю / *Less than once a week*<br>*2.* Один или два раза в неделю / Once or twice a week<br>3. Три или более раз в неделю / *Three or more times a week*
(c) Подергивания ногами во время сна / *Legs twitching or jerking while you sleep*	*0.* Ни разу в течение последнего месяца / *Not during the past month*<br>*1.* Менее, чем один раз в неделю / *Less than once a week*<br>*2.* Один или два раза в неделю / Once or twice a week<br>3. Три или более раз в неделю / *Three or more times a week*
(d) Эпизоды дезориентации или замешательства в период сна / *Episodes of disorientation or confusion during sleep*	*0.* Ни разу в течение последнего месяца / *Not during the past month*<br>*1.* Менее, чем один раз в неделю / *Less than once a week*<br>*2.* Один или два раза в неделю / Once or twice a week<br>3. Три или более раз в неделю / *Three or more times a week*
(e) Другие проявления беспокойного состояния во время Вашего сна: пожалуйста, опишите<br>/ Other restlessness while you sleep, please describe	

ТЕСТ НА УМСТВЕННЫЕ СПОСОБНОСТИ
MINI-MENTAL STATE EXAMINATION (MMSE)

Инструкция: Выставляется один бал за правильный ответ на каждый вопрос или действие
Instructions: Score one point for each correct response within each question or activity.

**Максимальный балл Maximum Score**	**Баллы, набранные пациентом Patient's Score**	**Вопросы Questions**
5		Какой сейчас год? Какое сейчас время года? Какая сегодня дата? Какой сегодня день недели? Какой сейчас месяц? *"What is the year? Season? Date? Day? Month?"*
5		Где мы сейчас находимся? В каком регионе? В какой стране? В каком городе? Как называется это учреждение? На каком этаже мы сейчас? *"Where are we now? State? County? Town/city? Hospital? Floor?"*
3		Экзаменатор четко и медленно называет три несвязанных объекта, затем инструктор просит пациента назвать всех троих. Ответ пациента используется для подсчета очков. Экзаменатор повторяет их, пока пациент не узнает их всех, если это возможно (Пример: стол, улица, бумага). *The examiner names three unrelated objects clearly and slowly, then the instructor asks the patient to name all three of them. The patient's response is used for scoring. The examiner repeats them until patient learns all of them, if possible.*
5		Вычтите 7 из 100 и далее вычитайте из того, что осталось, пока я не попрошу Вас остановиться. Альтернативный вопрос: повторите слово «СТОЛБ» вперёд (без оценки), потом по буквам в обратном порядке (оценивайте «1» каждую букву в правильному месте слова) *"I would like you to count backward from 100 by sevens." (93, 86, 79, 72, 65, …) Alternative: "Spell WORLD backwards." (D-L-R-O-W)*
3		Назовите 3 предмета которые я называл Вам ранее. *"Earlier I told you the names of three things. Can you tell me what those were?"*
2		Покажите пациенту два простых объекта, например, наручные часы и карандаш, и попросите пациента назвать их. *Show the patient two simple objects, such as a wristwatch and a pencil, and ask the patient to name them.*
1		Повторите фразу «Никаких если, никаких но»?? *"Repeat the phrase: 'No ifs, ands, or buts.'"*
3		Возьмете правой рукой лист бумаги, сложите его вдвое и положите на стол. *"Take the paper in your right hand, fold it in half, and put it on the floor." (The examiner gives the patient a piece of blank paper.)*
1		«Пожалуйста, прочитайте это и сделайте то, что написано». (Письменная инструкция «Закройте глаза»). *"Please read this and do what it says." (Written instruction is "Close your eyes.")*
1		«Составьте и напишите предложение о чем угодно» (это предложение должно содержать существительное и глагол.) *"Make up and write a sentence about anything." (This sentence must contain a noun and a verb.)*

1		Пожалуйста скопируйте данную фигуру (Экзаменатор дает пациенту чистый лист бумаги и просит его / ее изобразить фигуру. Все 10 углов должны присутствовать, а два должны пересекаться)<br>*"Please copy this picture." (The examiner gives the patient a blank piece of paper and asks him/her to draw the symbol below. All 10 angles must be present and two must intersect.)*<br>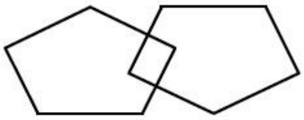
30		Всего TOTAL

СКРИНИНГ ЛИЧНОСТНЫХ НАРУШЕНИЕЙ
PERSONALITY DISORDERS SCREEN

Сейчас я собираюсь прочитать серию заявлений, которые люди используют для описания себя. Ответьте, правда или ложь для каждого утверждения. Лучшим ответом, как правило, является тот, который приходит вам на ум первым, поэтому не задумывайтесь, прежде чем отвечать. Вот первое утверждение.
Now, I am going to read a series of statements that people use to describe themselves. Answer true or false for each statement. The best answer is usually the one that comes to your mind first, so don't take too much time thinking before you answer. Here's the first statement.

**Вы бы сказали, что это верно или ложно для вас?**<br>(Would you say this is true or false for you?)	**правда true (1)**	**ложно false (5)**	**не знаю do not know (8)**	**отказался refused (9)**
Я показываю свои чувства, чтобы все могли видеть. Вы бы сказали, что это верно или ложь для вас?<br>*I show my feelings for everyone to see. Would you say this is true or false for you?*	1	5	8	9
Я часто чувствую себя «пустым» в душе.<br>*I often feel "empty" inside.*	1	5	8	9
Я очень подвержен перемене настроения.<br>*I'm very moody.*	1	5	8	9
Следуя некоторым своим стремлениям, это вызывает у меня проблемы.<br>*Giving in to some of my urges gets me into trouble.*	1	5	8	9
У меня есть истерики или вспышки гнева<br>*I have tantrums or angry outbursts*	1	5	8	9
Когда я испытываю стресс, вещи вокруг меня не кажутся реальным.<br>*When I'm under stress, things around me don't seem real.*	1	5	8	9
Я иду на крайности, чтобы попытаться не дать людям покинуть меня<br>*I go to extremes to try to keep people from leaving me.*	1	5	8	9
Меня никогда не арестовывали.<br>*I've never been arrested.*	1	5	8	9
Иногда я делал то, что могло послужить аресту человека.<br>*At times I've done things that could get a person arrested.*	1	5	8	9

Обычно я плохо себя чувствую, когда я делаю больно или расстраиваю кого-то.<br>*I usually feel bad when I hurt or upset someone.*	1	5	8	9
Иногда я отказывался от работы, даже когда от меня ожидали (надеялись на меня)<br>*At times I've refused to hold a job, even when I was expected to.*	1	5	8	9
Я буду лгать или жульничать, если это служит моей цели.<br>*I will lie or con someone if it serves my purpose.*	1	5	8	9
Я выхожу из себя и вступаю в драки.<br>*I lose my temper and get into physical fights.*	1	5	8	9
Я рискую и делаю безрассудные вещи.<br>*I take chances and do reckless things.*	1	5	8	9
Мне трудно избегать проблем.<br>*It's hard for me to stay out of trouble.*	1	5	8	9
Время от времени я не выполняю свои финансовые обязательства.<br>*At times I fail to meet my financial obligations.*	1	5	8	9
Иногда я намеренно повреждал вещи, которые не были моими.<br>*At times I've intentionally damaged things that weren't mine.*	1	5	8	9
Я предоставлю ложную информацию о себе, если это поможет мне получить работу или произвести впечатление на кого-то.<br>*I will give false information about myself if it will help me get a job or impress someone.*	1	5	8	9
Я спорю или дерусь, когда люди пытаются помешать мне делать то, что я хочу.<br>*I argue or fight when people try to stop me from doing what I want.*	1	5	8	9
Мои чувства подобны погоде, они всегда меняются.<br>*My feelings are like the weather, they're always changing.*	1	5	8	9
Иногда я злюсь до такой степени, что ломаю или разбиваю вещи.<br>*Sometimes I get so angry I break or smash things.*	1	5	8	9
Я позволяю другим принимать важные решения за меня.<br>*I let others make my big decisions for me.*	1	5	8	9
Обычно я чувствую себя некомфортно или беспомощно, когда я один.<br>*I usually feel uncomfortable or helpless when I'm alone.*	1	5	8	9
Я часто прошу совета или уверенности/ одобрения / поддержки в повседневных решениях.<br>*I often seek advice or reassurance about everyday decisions*.	1	5	8	9

Я являюсь самим собой (не меняю своё поведение), даже когда вокруг есть другие люди.<br>*I keep to myself even when there are other people around.*	1	5	8	9
Люди думают, что я слишком строг в отношении правил и предписаний.<br>*People think I'm too strict about rules and regulations.*	1	5	8	9
Люди думают, что я слишком жесткий или формальный.<br>*People think I'm too stiff or formal.*	1	5	8	9
Я чувствую себя неловко или неуместно в социальных ситуациях.<br>*I feel awkward or out of place in social situations.*	1	5	8	9
Люди часто высмеивают меня за моей спиной.<br>*People often make fun of me behind my back.*	1	5	8	9
Я предпочитаю действия, которые я могу делать самостоятельно.<br>*I prefer activities that I can do by myself.*	1	5	8	9
Я много лет испытывал неприязнь/ вражду к людям. *I've held grudges against people for years.*	1	5	8	9
Я убежден, что в мире есть заговор.<br>*I'm convinced there's a conspiracy behind many things in the world.*	1	5	8	9

Q. Психологический опрос / *Psychological survey*
Вопросы, относящиеся ко всему периоду жизни

**Q1**	Меня стало беспокоить что-то, что раньше не беспокоило.<br>*I was Bothered by Things that Usually don't Bother Me.*	*0* - Редко или ни разу (менее 1 дня) */ Rarely or none of the time (less than 1 day)*<br>*1* - Некоторое или небольшое время (1-2 дня) */ Some or a little of the time (1–2 days)*<br>*2* - Время от времени или умеренное количество времени (3-4 дня) */ Occasionally or a moderate amount of the time (3–4 days)*<br>*3* - Большинство или все время (5-7 дней) / *Most or all of the time (5–7 days)*
**Q2**	Я не хотел(а) есть, потому что у меня был плохой аппетит<br>*I did not Feel Like Eating, My Appetite was Poor*	*0* - Редко или ни разу (менее 1 дня) */ Rarely or none of the time (less than 1 day)*<br>*1* - Некоторое или небольшое время (1-2 дня) */ Some or a little of the time (1–2 days)*<br>*2* - Время от времени или умеренное количество времени (3-4 дня) */ Occasionally or a moderate amount of the time (3–4 days)*<br>*3* - Большинство или все время (5-7 дней) / *Most or all of the time (5–7 days)*
**Q3**	Я чувствовал(а), что не мог(ла) выбраться из подавленного состояния даже с помощью семьи и друзей *I Felt that I could not Shake off the Blues, Even with the Help from Family & Friends*	*0* - Редко или ни разу (менее 1 дня) */ Rarely or none of the time (less than 1 day)*<br>*1* - Некоторое или небольшое время (1-2 дня) */ Some or a little of the time (1–2 days)*<br>*2* - Время от времени или умеренное количество времени (3-4 дня) */ Occasionally or a moderate amount of the time (3–4 days)*

		3 - Большинство или все время (5-7 дней) / *Most or all of the time (5–7 days)*
**Q4**	Я чувствовал(а), что я такой же хороший(ая), как и другие<br>*I Felt that I was Just as Good as Other People*	*0* - Редко или ни разу (менее 1 дня) / *Rarely or none of the time (less than 1 day)*<br>*1* - Некоторое или небольшое время (1-2 дня) / *Some or a little of the time (1–2 days)*<br>*2* - Время от времени или умеренное количество времени (3-4 дня) / *Occasionally or a moderate amount of the time (3–4 days)*<br>*3* - Большинство или все время (5-7 дней) / *Most or all of the time (5–7 days)*
**Q5**	У меня возникают трудности сосредоточиться на том, что я делал(а)<br>*I had Trouble Keeping My Mind on What I was Doing*	*0* - Редко или ни разу (менее 1 дня) / *Rarely or none of the time (less than 1 day)*<br>*1* - Некоторое или небольшое время (1-2 дня) / *Some or a little of the time (1–2 days)*<br>*2* - Время от времени или умеренное количество времени (3-4 дня) / *Occasionally or a moderate amount of the time (3–4 days)*<br>*3* - Большинство или все время (5-7 дней) / *Most or all of the time (5–7 days)*
**Q6**	Я чувствовал(а) себя подавленным<br>*I Felt Depressed*	*0* - Редко или ни разу (менее 1 дня) / *Rarely or none of the time (less than 1 day)*<br>*1* - Некоторое или небольшое время (1-2 дня) / *Some or a little of the time (1–2 days)*<br>*2* - Время от времени или умеренное количество времени (3-4 дня) / *Occasionally or a moderate amount of the time (3–4 days)*<br>*3* - Большинство или все время (5-7 дней) / *Most or all of the time (5–7 days)*
**Q7**	Я чувствовал(а), что все, что я сделал, было сделано с усилием<br>*I Felt that Everything I did was an Effort*	*0* - Редко или ни разу (менее 1 дня) / *Rarely or none of the time (less than 1 day)*<br>*1* - Некоторое или небольшое время (1-2 дня) / *Some or a little of the time (1–2 days)*<br>*2* - Время от времени или умеренное количество времени (3-4 дня) / *Occasionally or a moderate amount of the time (3–4 days)*<br>*3* - Большинство или все время (5-7 дней) / *Most or all of the time (5–7 days)*
**Q8**	Я был полным надежд на будущее<br>*I Felt Hopeful about the Future*	*0* - Редко или ни разу (менее 1 дня) / *Rarely or none of the time (less than 1 day)*<br>*1* - Некоторое или небольшое время (1-2 дня) / *Some or a little of the time (1–2 days)*<br>*2* - Время от времени или умеренное количество времени (3-4 дня) / *Occasionally or a moderate amount of the time (3–4 days)*<br>*3* - Большинство или все время (5-7 дней) / *Most or all of the time (5–7 days)*
**Q9**	Я думал, моя жизнь неудачна<br>*I Thought My Life had been a Failure*	*0* - Редко или ни разу (менее 1 дня) / *Rarely or none of the time (less than 1 day)*<br>*1* - Некоторое или небольшое время (1-2 дня) / *Some or a little of the time (1–2 days)*<br>*2* - Время от времени или умеренное количество времени (3-4 дня) / *Occasionally or a moderate amount of the time (3–4 days)*

		3 - Большинство или все время (5-7 дней) / *Most or all of the time (5–7 days)*
**Q10**	Я чувствовал(а) опасность<br>*I Felt Fearful*	*0* - Редко или ни разу (менее 1 дня) / *Rarely or none of the time (less than 1 day)*<br>*1* - Некоторое или небольшое время (1-2 дня) / *Some or a little of the time (1–2 days)*<br>*2* - Время от времени или умеренное количество времени (3-4 дня) / *Occasionally or a moderate amount of the time (3–4 days)*<br>*3* - Большинство или все время (5-7 дней) / *Most or all of the time (5–7 days)*
**Q11**	У меня беспокойный сон<br>*My Sleep was Restless*	*0* - Редко или ни разу (менее 1 дня) / *Rarely or none of the time (less than 1 day)*<br>*1* - Некоторое или небольшое время (1-2 дня) / *Some or a little of the time (1–2 days)*<br>*2* - Время от времени или умеренное количество времени (3-4 дня) / *Occasionally or a moderate amount of the time (3–4 days)*<br>*3* - Большинство или все время (5-7 дней) / *Most or all of the time (5–7 days)*
**Q12**	Я был счастлив(а)<br>*I was Happy*	*0* - Редко или ни разу (менее 1 дня) / *Rarely or none of the time (less than 1 day)*<br>*1* - Некоторое или небольшое время (1-2 дня) / *Some or a little of the time (1–2 days)*<br>*2* - Время от времени или умеренное количество времени (3-4 дня) / *Occasionally or a moderate amount of the time (3–4 days)*<br>*3* - Большинство или все время (5-7 дней) / *Most or all of the time (5–7 days)*
**Q13**	Я говорил(а) меньше, чем обычно *I Talked Less than Usual*	*0* - Редко или ни разу (менее 1 дня) / *Rarely or none of the time (less than 1 day)*<br>*1* - Некоторое или небольшое время (1-2 дня) / *Some or a little of the time (1–2 days)*<br>*2* - Время от времени или умеренное количество времени (3-4 дня) / *Occasionally or a moderate amount of the time (3–4 days)*<br>*3* - Большинство или все время (5-7 дней) / *Most or all of the time (5–7 days)*
**Q14**	Я чувствовал(а) себя одиноким<br>*I Felt Lonely*	*0* - Редко или ни разу (менее 1 дня) / *Rarely or none of the time (less than 1 day)*<br>*1* - Некоторое или небольшое время (1-2 дня) / *Some or a little of the time (1–2 days)*<br>*2* - Время от времени или умеренное количество времени (3-4 дня) / *Occasionally or a moderate amount of the time (3–4 days)*<br>*3* - Большинство или все время (5-7 дней) / *Most or all of the time (5–7 days)*
**Q15**	Люди были недружелюбны ко мне<br>*People were Unfriendly*	*0* - Редко или ни разу (менее 1 дня) / *Rarely or none of the time (less than 1 day)*<br>*1* - Некоторое или небольшое время (1-2 дня) / *Some or a little of the time (1–2 days)*<br>*2* - Время от времени или умеренное количество времени (3-4 дня) / *Occasionally or a moderate amount of the time (3–4 days)*

		3 - Большинство или все время (5-7 дней) / *Most or all of the time (5–7 days)*
**Q16**	Я наслаждался(ась) жизнью<br>*I Enjoyed Life*	*0* - Редко или ни разу (менее 1 дня) */ Rarely or none of the time (less than 1 day)*<br>*1* - Некоторое или небольшое время (1-2 дня) */ Some or a little of the time (1–2 days)*<br>*2* - Время от времени или умеренное количество времени (3-4 дня) */ Occasionally or a moderate amount of the time (3–4 days)*<br>*3* - Большинство или все время (5-7 дней) / *Most or all of the time (5–7 days)*
**Q17**	У меня были приступы плача<br>*I had Crying Spells*	*0* - Редко или ни разу (менее 1 дня) */ Rarely or none of the time (less than 1 day)*<br>*1* - Некоторое или небольшое время (1-2 дня) */ Some or a little of the time (1–2 days)*<br>*2* - Время от времени или умеренное количество времени (3-4 дня) */ Occasionally or a moderate amount of the time (3–4 days)*<br>*3* - Большинство или все время (5-7 дней) / *Most or all of the time (5–7 days)*
**Q18**	Мне было грустно<br>*I Felt Sad*	*0* - Редко или ни разу (менее 1 дня) */ Rarely or none of the time (less than 1 day)*<br>*1* - Некоторое или небольшое время (1-2 дня) */ Some or a little of the time (1–2 days)*<br>*2* - Время от времени или умеренное количество времени (3-4 дня) */ Occasionally or a moderate amount of the time (3–4 days)*<br>*3* - Большинство или все время (5-7 дней) / *Most or all of the time (5–7 days)*
**Q19**	Я чувствовал(а), что я не нравлюсь людям<br>*I Felt that People Dislike Me*	*0* - Редко или ни разу (менее 1 дня) */ Rarely or none of the time (less than 1 day)*<br>*1* - Некоторое или небольшое время (1-2 дня) */ Some or a little of the time (1–2 days)*<br>*2* - Время от времени или умеренное количество времени (3-4 дня) */ Occasionally or a moderate amount of the time (3–4 days)*<br>*3* - Большинство или все время (5-7 дней) / *Most or all of the time (5–7 days)*
**Q20**	Я не могу собраться<br>*I Could Not Get "Going"*	*0* - Редко или ни разу (менее 1 дня) */ Rarely or none of the time (less than 1 day)*<br>*1* - Некоторое или небольшое время (1-2 дня) */ Some or a little of the time (1–2 days)*<br>*2* - Время от времени или умеренное количество времени (3-4 дня) */ Occasionally or a moderate amount of the time (3–4 days)*<br>*3* - Большинство или все время (5-7 дней) / *Most or all of the time (5–7 days)*
**Q21**	Я чувствовал(а) радость<br>*I Feel Pleasant*	*0* - Редко или ни разу (менее 1 дня) */ Rarely or none of the time (less than 1 day)*<br>*1* - Некоторое или небольшое время (1-2 дня) */ Some or a little of the time (1–2 days)*<br>*2* - Время от времени или умеренное количество времени (3-4 дня) */ Occasionally or a moderate amount of the time (3–4 days)*

		3 - Большинство или все время (5-7 дней) / *Most or all of the time (5–7 days)*
**Q22**	Я быстро устаю<br>*I Tire Quickly*	*0* - Редко или ни разу (менее 1 дня) / *Rarely or none of the time (less than 1 day)*<br>*1* - Некоторое или небольшое время (1-2 дня) / *Some or a little of the time (1–2 days)*<br>*2* - Время от времени или умеренное количество времени (3-4 дня) / *Occasionally or a moderate amount of the time (3–4 days)*<br>*3* - Большинство или все время (5-7 дней) / *Most or all of the time (5–7 days)*
**Q23**	Мне хочется плакать<br>*I Feel Like Crying*	*0* - Редко или ни разу (менее 1 дня) / *Rarely or none of the time (less than 1 day)*<br>*1* - Некоторое или небольшое время (1-2 дня) / *Some or a little of the time (1–2 days)*<br>*2* - Время от времени или умеренное количество времени (3-4 дня) / *Occasionally or a moderate amount of the time (3–4 days)*<br>*3* - Большинство или все время (5-7 дней) / *Most or all of the time (5–7 days)*
**Q24**	Я хотел(а) бы быть таким же счастливым, как выглядят другие<br>*I Wish I Could be as Happy as Others Seem to be*	*0* - Редко или ни разу (менее 1 дня) / *Rarely or none of the time (less than 1 day)*<br>*1* - Некоторое или небольшое время (1-2 дня) / *Some or a little of the time (1–2 days)*<br>*2* - Время от времени или умеренное количество времени (3-4 дня) / *Occasionally or a moderate amount of the time (3–4 days)*<br>*3* - Большинство или все время (5-7 дней) / *Most or all of the time (5–7 days)*
**Q25**	Я претерпеваю неудачу, потому что я не могу быстро собраться с мыслями<br>*/I am Losing Out on Things Because I can't Make up My Mind soon Enough*	*0* - Редко или ни разу (менее 1 дня) / *Rarely or none of the time (less than 1 day)*<br>*1* - Некоторое или небольшое время (1-2 дня) / *Some or a little of the time (1–2 days)*<br>*2* - Время от времени или умеренное количество времени (3-4 дня) / *Occasionally or a moderate amount of the time (3–4 days)*<br>*3* - Большинство или все время (5-7 дней) / *Most or all of the time (5–7 days)*
**Q26**	Я чувствую себя отдохнувшим/<br>*I Feel Rested*	*0* - Редко или ни разу (менее 1 дня) / *Rarely or none of the time (less than 1 day)*<br>*1* - Некоторое или небольшое время (1-2 дня) / *Some or a little of the time (1–2 days)*<br>*2* - Время от времени или умеренное количество времени (3-4 дня) / *Occasionally or a moderate amount of the time (3–4 days)*<br>*3* - Большинство или все время (5-7 дней) / *Most or all of the time (5–7 days)*
**Q27**	Я Спокоен(на), невозмутим(а) и собран(а)<br>*I am " Calm, Cool & Collected*	*0* - Редко или ни разу (менее 1 дня) / *Rarely or none of the time (less than 1 day)*<br>*1* - Некоторое или небольшое время (1-2 дня) / *Some or a little of the time (1–2 days)*<br>*2* - Время от времени или умеренное количество времени (3-4 дня) / *Occasionally or a moderate amount of the time (3–4 days)*

		3 - Большинство или все время (5-7 дней) / *Most or all of the time (5–7 days)*
**Q28**	Я чувствую, что трудности накапливаются так, что я не могу преодолеть их *//I Feel that Difficultiles are Piling up so that I can't Overcome them*	*0* - Редко или ни разу (менее 1 дня) */ Rarely or none of the time (less than 1 day)*<br>*1* - Некоторое или небольшое время (1-2 дня) */ Some or a little of the time (1–2 days)*<br>*2* - Время от времени или умеренное количество времени (3-4 дня) */ Occasionally or a moderate amount of the time (3–4 days)*<br>*3* - Большинство или все время (5-7 дней) / *Most or all of the time (5–7 days)*
**Q29**	Я слишком беспокоюсь о том, что действительно не имеет значения<br>*I Worry too Much Over Something that Really doesn't Matter*	*0* - Редко или ни разу (менее 1 дня) */ Rarely or none of the time (less than 1 day)*<br>*1* - Некоторое или небольшое время (1-2 дня) */ Some or a little of the time (1–2 days)*<br>*2* - Время от времени или умеренное количество времени (3-4 дня) */ Occasionally or a moderate amount of the time (3–4 days)*<br>*3* - Большинство или все время (5-7 дней) / *Most or all of the time (5–7 days)*
**Q30**	Я счастлив(а)<br>*I am Happy*	*0* - Редко или ни разу (менее 1 дня) */ Rarely or none of the time (less than 1 day)*<br>*1* - Некоторое или небольшое время (1-2 дня) */ Some or a little of the time (1–2 days)*<br>*2* - Время от времени или умеренное количество времени (3-4 дня) */ Occasionally or a moderate amount of the time (3–4 days)*<br>*3* - Большинство или все время (5-7 дней) / *Most or all of the time (5–7 days)*
**Q31**	Я склонен принимать вещи близко к сердцу<br>*I am Inclined to Take Things Hard*	*0* - Редко или ни разу (менее 1 дня) */ Rarely or none of the time (less than 1 day)*<br>*1* - Некоторое или небольшое время (1-2 дня) */ Some or a little of the time (1–2 days)*<br>*2* - Время от времени или умеренное количество времени (3-4 дня) */ Occasionally or a moderate amount of the time (3–4 days)*<br>*3* - Большинство или все время (5-7 дней) / *Most or all of the time (5–7 days)*
**Q32**	Мне не хватает уверенности<br>*I Lack Self-Confidence*	*0* - Редко или ни разу (менее 1 дня) */ Rarely or none of the time (less than 1 day)*<br>*1* - Некоторое или небольшое время (1-2 дня) */ Some or a little of the time (1–2 days)*<br>*2* - Время от времени или умеренное количество времени (3-4 дня) */ Occasionally or a moderate amount of the time (3–4 days)*<br>*3* - Большинство или все время (5-7 дней) / *Most or all of the time (5–7 days)*
**Q33**	Я чувствую себя в безопасности<br>/ I feel safe	*0* - Редко или ни разу (менее 1 дня) */ Rarely or none of the time (less than 1 day)*<br>*1* - Некоторое или небольшое время (1-2 дня) */ Some or a little of the time (1–2 days)*<br>*2* - Время от времени или умеренное количество времени (3-4 дня) */ Occasionally or a moderate amount of the time (3–4 days)*

		3 - Большинство или все время (5-7 дней) / *Most or all of the time (5–7 days)*
**Q34**	Я стараюсь избегать столкновений с кризисами или трудностями<br>*I Try to Avoid Facing a Crises or Difficulty*	*0* - Редко или ни разу (менее 1 дня) */ Rarely or none of the time (less than 1 day)*<br>*1* - Некоторое или небольшое время (1-2 дня) */ Some or a little of the time (1–2 days)*<br>*2* - Время от времени или умеренное количество времени (3-4 дня) */ Occasionally or a moderate amount of the time (3–4 days)*<br>*3* - Большинство или все время (5-7 дней) / *Most or all of the time (5–7 days)*
**Q35**	У меня плохое настроение<br>*I Feel Blue*	*0* - Редко или ни разу (менее 1 дня) */ Rarely or none of the time (less than 1 day)*<br>*1* - Некоторое или небольшое время (1-2 дня) */ Some or a little of the time (1–2 days)*<br>*2* - Время от времени или умеренное количество времени (3-4 дня) */ Occasionally or a moderate amount of the time (3–4 days)*<br>*3* - Большинство или все время (5-7 дней) / *Most or all of the time (5–7 days)*
**Q36**	Я удовлетворен<br>*I am Content*	*0* - Редко или ни разу (менее 1 дня) */ Rarely or none of the time (less than 1 day)*<br>*1* - Некоторое или небольшое время (1-2 дня) */ Some or a little of the time (1–2 days)*<br>*2* - Время от времени или умеренное количество времени (3-4 дня) */ Occasionally or a moderate amount of the time (3–4 days)*<br>*3* - Большинство или все время (5-7 дней) / *Most or all of the time (5–7 days)*
**Q37**	Некоторые неважные мысли пробегают в моей голове и беспокоят меня / *Some Unimportant Thoughts Run Through My Mind & Bother Me*	*0* - Редко или ни разу (менее 1 дня) */ Rarely or none of the time (less than 1 day)*<br>*1* - Некоторое или небольшое время (1-2 дня) */ Some or a little of the time (1–2 days)*<br>*2* - Время от времени или умеренное количество времени (3-4 дня) */ Occasionally or a moderate amount of the time (3–4 days)*<br>*3* - Большинство или все время (5-7 дней) / *Most or all of the time (5–7 days)*
**Q38**	Я чувствую разочарование так остро, что не могу выкинуть эти мысли из головы<br>*I Take Disappointments so Keenly that I can't Put Them Out of My Mind*	*0* - Редко или ни разу (менее 1 дня) */ Rarely or none of the time (less than 1 day)*<br>*1* - Некоторое или небольшое время (1-2 дня) */ Some or a little of the time (1–2 days)*<br>*2* - Время от времени или умеренное количество времени (3-4 дня) */ Occasionally or a moderate amount of the time (3–4 days)*<br>*3* - Большинство или все время (5-7 дней) / *Most or all of the time (5–7 days)*
**Q39**	Я устойчивый человек<br>*I am a Steady Person*	*0* - Редко или ни разу (менее 1 дня) */ Rarely or none of the time (less than 1 day)*<br>*1* - Некоторое или небольшое время (1-2 дня) */ Some or a little of the time (1–2 days)*<br>*2* - Время от времени или умеренное количество времени (3-4 дня) */ Occasionally or a moderate amount of the time (3–4 days)*

		3 - Большинство или все время (5-7 дней) / *Most or all of the time (5–7 days)*
**Q40**	Я нахожусь в состоянии напряженности или потрясения, так как я обдумываю свои последние проблемы и интересы *I Get in a State of Tension or Turmoil as I Think Over My Recent Concerns and Interest so*	*0* - Редко или ни разу (менее 1 дня) / *Rarely or none of the time (less than 1 day)*<br>*1* - Некоторое или небольшое время (1-2 дня) / *Some or a little of the time (1–2 days)*<br>*2* - Время от времени или умеренное количество времени (3-4 дня) / *Occasionally or a moderate amount of the time (3–4 days)*<br>*3* - Большинство или все время (5-7 дней) / *Most or all of the time (5–7 days)*
**Q41**	Совершали ли Вы попытки самоубийства?<br>*Attempted Suicide*	*0* - Редко или ни разу (менее 1 дня) / *Rarely or none of the time (less than 1 day)*<br>*1* - Некоторое или небольшое время (1-2 дня) / *Some or a little of the time (1–2 days)*<br>*2* - Время от времени или умеренное количество времени (3-4 дня) / *Occasionally or a moderate amount of the time (3–4 days)*<br>*3* - Большинство или все время (5-7 дней) / *Most or all of the time (5–7 days)*
**Q42**	Почему? *Why*	
**Q43**	Была ли попытка самоубийства по финансовым причинам?<br>*Attempted Suicide due to Financial Reasons then*	*0* - Редко или ни разу (менее 1 дня) / *Rarely or none of the time (less than 1 day)*<br>*1* - Некоторое или небольшое время (1-2 дня) / *Some or a little of the time (1–2 days)*<br>*2* - Время от времени или умеренное количество времени (3-4 дня) / *Occasionally or a moderate amount of the time (3–4 days)*<br>*3* - Большинство или все время (5-7 дней) / *Most or all of the time (5–7 days)*
**Q44**	Задумывались ли Вы о самоубийстве в течение последних 6 месяцев или раньше? / *Have you Thought of Committing Suicide in the Last 6 Months or Earlier*	*0* - Редко или ни разу (менее 1 дня) / *Rarely or none of the time (less than 1 day)*<br>*1* - Некоторое или небольшое время (1-2 дня) / *Some or a little of the time (1–2 days)*<br>*2* - Время от времени или умеренное количество времени (3-4 дня) / *Occasionally or a moderate amount of the time (3–4 days)*<br>*3* - Большинство или все время (5-7 дней) / *Most or all of the time (5–7 days)*
**Q45**	Думали ли Вы о самоубийстве? / *Thought of Suicide*	*0* - Редко или ни разу (менее 1 дня) / *Rarely or none of the time (less than 1 day)*<br>*1* - Некоторое или небольшое время (1-2 дня) / *Some or a little of the time (1–2 days)*<br>*2* - Время от времени или умеренное количество времени (3-4 дня) / *Occasionally or a moderate amount of the time (3–4 days)*<br>*3* - Большинство или все время (5-7 дней) / *Most or all of the time (5–7 days)*
**Q46**	Почему? / *Why*	
**Q47**	Думали ли в таком случае о самоубийстве из-за финансовых проблем? / *Thought of Suicide due to*	*0* - Редко или ни разу (менее 1 дня) / *Rarely or none of the time (less than 1 day)*<br>*1* - Некоторое или небольшое время (1-2 дня) / *Some or a*

	Financial Reasons then	*little of the time (1–2 days)*<br><br>*2* - Время от времени или умеренное количество времени (3-4 дня) / *Occasionally or a moderate amount of the time (3–4 days)*<br>*3* - Большинство или все время (5-7 дней) / *Most or all of the time (5–7 days)*

Дополнительные вопросы по эмоциональному статусу:

Что самое плохое было в вашей жизни?<br>*What was the worst thing about your life?*	
Наиболее счастливый момент в вашей жизни?<br>*The happiest moment in your life?*	
Что Вас больше всего беспокоит на сегодняшний день?<br>(выбрать один пункт)<br>*What are you most worried about today?*<br>*(choose one item)*	1. Зрение / *Vision*<br>2. Слух / *Hearing*<br>3. Передвижение / *Move*<br>4. Нехватка общения / *Lack of communication*<br>5. Эмоциональная нестабильность / *Emotional instability*<br>6. Общее состояние организма / *General health*<br>7. Одиночество / *Loneliness*<br>8. Материальное благосостояние / *prosperity*

Какое событие в Вашей жизни по тяжести и эмоциональной напряженности самое негативное:
What has been your most impressive negative experience in your life:

1. Период Великой Отечественной войны или послевоенный период
   bad experience during or after the Great War,
2. Семейные проблемы
   family problems,
3. Проблемы со здоровьем
   health problems,
4. Другое
   other

Опишите это событие в свободной форме / *Describe this event*

Заключение врача по эмоциональному статусу участника исследования (вписать в свободной форме):
The doctor's conclusion about the emotional status of the participant in the study:

**Тест Вебера**/<br>*Webers test*	1. **> правое ухо /** *Right ear*<br>2. **> левое ухо /** *Left ear*<br>3. **Одинаково /** *equally*

**Тест Ринне/**<br>*Rinne test*	
**Правое ухо/**<br>*Right ear*	**1. Положительно/***positive*<br>**2. Отрицательно/***negative*
**Левое ухо/**<br>*Left ear*	**1. Положительно/** *positive*<br>**2. Отрицательно/** *negative*

30. Спирометрия/ Spirometry

**Показатели/**<br>*Indicators*	**Результаты/** *Results*
**ДО – дыхательный объем (мл) /**<br>*TV= Tidal volume (ml)*	
**ЖЕЛ – жизненная емкость легких (мл) /**<br>*VC = Vital Capacity (ml)*	
**ФЖЕЛ – форсированная жизненная емкость легких (%) /**<br>*FVC* **=** *forced vital capacity (%)*	
**ОФВ1 – объем форсированного выдоха за первую секунду** /<br>*FEV1 = forced expiratory volume in 1 sec*	
**Индекс Генслера или (ОФВ1/ФЖЕЛ)×100% /**<br>(*FEV1/FVC)×100% = Index Gaenslar*	
**Индекс Тиффно или (ОФВ1/ЖЕЛ)×100% /**<br>*FEV1 = (forced expiratory volume in 1 sec / VC)×100% = Vital Capacity (%)*	

31. Динамометрия/ *Dynamometry*

**Доминирующая рука /** *Dominant hand*	1. Правая / Right<br>2. Левая / Left
**Правая рука (daN)** / *Right hand (daN)*	
**Левая рука (daN) /** *Left hand (daN)*	

**32. Выполнено ЭКГ**<br>***ECG performed***	1. Да/ *Yes*	2.Нет /*No*

“Ural Study on Very Old” (USVO)

Офтальмологическое обследование

Ophthalmic examination

A. Имя/*Name* ____

Порядковый номер пациента / *Identification No* | | | | | |

**B**. Жалобы:<br>*Ocular Complaints:*		
1.**Снижение зрения:**<br>*Diminished Vision*	1. Да/ *Yes*	2.Нет /*No*
2.**Покраснение:**<br>*Redness*	1. Да/ *Yes*	2.Нет /*No*
3.**Боль:**<br>*Pain*	1. Да/ *Yes*	2.Нет /*No*
4.**Слизистые выделения**<br>*Watery discharge*	1. Да/ *Yes*	2.Нет /*No*
5.**«Плавающие мушки»**<br>*Floaters*	1. Да/ *Yes*	2.Нет /*No*
6. **Сухость в глазах**<br>*Dry Eye*	1. Да/ *Yes*	2.Нет /*No*
7. **Светобоязнь**<br>*Increased glare*	1. Да/ *Yes*	2.Нет /*No*
8. **Другое** /<br>*Others*	1. Да/ *Yes*	2.Нет /*No*

Опросник на наличие синдрома сухого глаза/

Dry Eye

Z_1. Чувствовали ли Вы когда-нибудь сухость глаза? /<br>*Do your eyes ever feel dry?*	1 –нет / *none*<br>2 – редко или иногда / *rarely or sometimes*<br>3 – часто или все время / *frequently or always*
Z_2. Чувствовали ли Вы ощущение песка в глазах когда-либо? /<br>*Do you ever feel a gritty or sandy sensation in your eye?*	1 –нет / *none*<br>2 – редко или иногда / *rarely or sometimes*<br>3 – часто или все время / *frequently or always*
Z_3. Ощущали ли Вы жжение в глазах когда-либо? /<br>*Do your eyes ever have a burning sensation?*	1 –нет / *none*<br>2 – редко или иногда / *rarely or sometimes*<br>3 – часто или все время / *frequently or always*
Z_4. Бывают ли Ваши глаза красными? /<br>*Are your eyes ever red?*	1 –нет / *none*<br>2 – редко или иногда / *rarely or sometimes*<br>3 – часто или все время / *frequently or always*
Z_5. Замечали ли Вы наличие корочек на ресницах? /<br>*Do you notice much crusting on your lashes?*	1 –нет / *none*<br>2 – редко или иногда / *rarely or sometimes*<br>3 – часто или все время / *frequently or always*
Z_6. Испытывали ли Вы трудности при открытии глаз по утрам когда-либо? /<br>*Do your eyes ever get stuck in the morning?*	1 –нет / *none*<br>2 – редко или иногда / *rarely or sometimes*<br>3 – часто или все время / *frequently or always*

История болезни глаз / Ocular History

C. Применения лекарства:
Using Medications:

1.Антиглаукомные:<br>*Anti Glaucoma Medicines:*	1. Да/ *Yes*	2.Нет /*No*	с какого времени/*since*
2.Дексаметазон:<br>*Steroids:*	1. Да/ *Yes*	2.Нет /*No*	с какого времени/*since*
3.Другое<br>*Others:*	1. Да/ *Yes*	2.Нет /*No*	с какого времени/*since*
**D.** Были ли травмы глаза? *H/O.Trauma:*	1. Оба глаза / *Both Eyes*<br>2. Левый глаз / *Left Eye*<br>3. Правый глаз / *Right Eye*<br>*4.* Ни один / *None*		

Осмотр: / *Examination:*

E. Оценка остроты зрения / *Visual Assesment*

	OD	OS
Авторефракция (средняя) (до мидриаза)<br>*Automated Refraction (Average)(before dilatation)*	Sph<br>Cyl<br>Ax	Sph<br>Cyl<br>Ax

Определение остроты зрения вдаль без коррекции / UCVA

Острота зрения	OD	OS	OU
По таблице Сивцева - Головина			
По таблице ETDRS			

Таблица расстояний/ *Distance Chart*	OD	OS	OU
4 метра/*4 metres*	1	1	1
2 метра/ *2 metres*	2	2	2
1 метр/*1 metres*	3	3	3

Субъективная рефракция<br>*Subjective Refraction*	Sph<br>Cyl<br>Ax	Sph<br>Cyl<br>Ax

Определение остроты зрения вдаль в очках пациента / *VA with presenting glasses*

Острота зрения	OD	OS	OU
По таблице Сивцева - Головина			
По таблице ETDRS			

Таблица расстояний/ *Distance Chart*	OD	OS	OU
4 метра/*4 metres*	1	1	1
2 метра/ *2 metres*	2	2	2
1 метр/*1 metres*	3	3	3

Определение остроты зрения вдаль с максимальной коррекцией / BCVA

Острота зрения	OD	OS	OU
По таблице Сивцева - Головина			
По таблице ETDRS			

Таблица расстояний/ *Distance Chart*	OD	OS	OU
4 метра/*4 metres*	1	1	1
2 метра/ *2 metres*	2	2	2
1 метр/*1 metres*	3	3	3

**Если низкая острота зрения**<br>Где 1.8 - эквивалент высшей линии логарифма прочтенного с 1 метра<br>*If visual acuity <1.8 (Where 1.8 is equivalent to the top line the LogMar read at 1metre).*	OD	OS
Счет пальцев на расстоянии пол метра<br>*Counting Fingers at 1/2metre (CF)*	1	1
Движения руки<br>*Hand Movements (HM)*	2	2
Восприятие света<br>*Light Perception (LP)*	3	3
Не воспринимает свет<br>*No Perception of Light (NPL)*	4	4

<u>Острота зрения вблизи</u>

Определение остроты зрения вблизи без коррекции /
near UCVA

Vis OD	Vis OS	Vis OU

Определение остроты зрения вблизи в очках пациента
(если пациент без очков - острота зрения определяется без очков) /
near VA with presenting glasses (with the glasses the patient wears, and if the patient does not have glasses, measurement is without glasses)

Vis OD	Vis OS	Vis OU

Определение остроты зрения вблизи с коррекцией рефракционной ошибки вдаль плюс аддидация в +3 D
/ *near BCVA with best correcting distant refractive error plus an addition of +3 D*

Vis OD	Vis OS	Vis OU

Цветовая гаплоскопия
Цветовая гаплоскопия без коррекции / *UCVA*

Четырехточечный цветотест / *W4LT*	1.монокулярное OD / *monocular OD*<br>2.монокулярное OS / *monocular OS*<br>3.бинокулярное / *binocular*<br>4.одновременное / *simultaneous*

Цветовая гаплоскопия с максимальной коррекцией / *BCVA*

Четырехточечный цветотест / *W4LT*	1.монокулярное OD / *monocular OD*<br>2.монокулярное OS / *monocular OS*<br>3.бинокулярное / *binocular*<br>4.одновременное / *simultaneous*

**F. Движение глаз:** *Ocular Movements*	OD/ Правый глаз	OS/ Левый глаз
	1. движения в полном объеме/*Full*<br>2. ограничено/*Limited*	1. движения в полном объеме/*Full*<br>2. ограничено/*Limited*

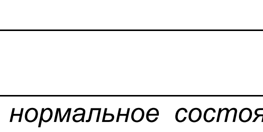
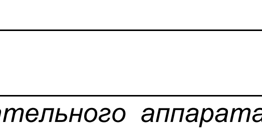

**G. Косоглазие:** ***Squint:***	*OD*	*OS*
Ортофория / *Orthophoria - нормальное состояние двигательного аппарата глаз, зрительные оси параллельны.*	**1**	**1**
Экзофория / *Exophoria - тенденция глаза к отклонению кнаружи при отсутствии внешних зрительных раздражителей*	**2**	**2**

Гиперфория / *Hyperphoria - вид гетерофории, характеризующийся тенденцией к отклонению глаза кверху*	**3**	**3**
Гипофория / *Hypophoria - вид гетерофории, характеризующийся тенденцией к отклонению глаза книзу*	**4**	**4**
Экзотропия / *Exotropia - расходящееся косоглазие*	**5**	**5**
Гипертропия / *Hypertropia – вертикальное косоглазие, при котором косящий глаз отклоняется кверху*	**6**	**6**
Гипотропия / *Hypotropia - вертикальное косоглазие, при котором косящий глаз отклоняется книзу*	**7**	**7**
Циклотропия / Cyclo*tropia - вид косоглазия, в котором, есть вращение одного глаза (или обоих) относительно зрительной оси. Следовательно, поля зрения обоих глаз наклонены по отношению друг к другу.*	**8**	**8**
Циклофория / Cyclo*phoria - разновидность косоглазия, при которой глаз во время обследования немного вращается по часовой или против часовой стрелки.*	**9**	**9**
Эзофория / *Esophoria - тенденция глаза отклоняться вовнутрь, в направлении к носу при отсутствии внешних зрительных раздражителей*	**10**	**10**
Эзотропия / *Esotropia – сходящееся косоглазие*	**11**	**11**

G1. Диагноз косоглазия / *Diagnosis Squint*

Содружественный / *Concomitant*	1
Паралитический / *Paralytic*	2
Ретракционный синдром Дуэйна / *Duane's*	3
Другое / *Others*	4

**H. Нистагм**<br>*Nystagmus*	1. Присутствует/ Present<br>2. Отсутствует/ Absent
**I.Неврологические расстройства и и паралич нерва:**<br>*Neurological Deficit: & Nerve Palsy*	1. Присутствует/ Present<br>2. Отсутствует/ Absent

**Ультразвуковая биометрия**	**OD**	**OS**
**Передне-задняя ось глаза (мм) /** *AL*		
**Толщина хрусталика /** *TL*		

**Исследование функции мейбомиевых желез /** *meibomian gland dysfunction*	OD	OS
0 – наличие телеангиоэктазий, но отсутствие закупорки / *no obstruction*<br>1 – мейбомиевые железы закупорены, при компрессии - полупрозрачный серозный секрет / *plugged with translucent serous secretion when the lid margin was compressed*,<br>2 – мейбомиевые железы закупорены, при компрессии - вязкий или восковый белковый секрет / *plugged with viscous or waxy white secretion when the lid margin was compressed*,<br>3 – закупорены, при компрессии – секрет отсутствует. / *plugged with no secretion when the lid margin was compressed)*<br>*4 – норма / normal*	0<br>1<br>2<br>3<br>4	0<br>1<br>2<br>3<br>4
**Тест Ширмера** / *Shirmer's test (mm)*		

Биомикроскопия / *Slit Lamp Examination:*
(Нужное обвести)

**J. Операции на органе зрения:** ***J. Surgery done***	**OD**		**OS**	
Были ли операции?/ *none*	1. Да/ *Yes*	2.Нет /*No*	1. Да/ *Yes*	2.Нет /*No*
По поводу катаракты / *Cataract*	1. Да/ *Yes*	2.Нет /*No*	1. Да/ *Yes*	2.Нет /*No*
По поводу глаукомы *Glaucoma*	1. Да/ *Yes*	2.Нет /*No*	1. Да/ *Yes*	2.Нет /*No*
Операции по поводу травмы *Trauma*	1. Да/ *Yes*	2.Нет /*No*	1. Да/ *Yes*	2.Нет /*No*
Другие операции*Other,Specify*	1. Да/ *Yes*	2.Нет /*No*	1. Да/ *Yes*	2.Нет /*No*

**K. Веки:** ***K. Lids***	**OD**	**OS**
Норма/ *Normal*	1	1
Заворот века/ *Entropion*	2	2
Выворот/ *Ectropion*	3	3
Трихиаз/ *Trichiasis*	4	4
Другое/ *Others*	5	5
**L. Конъюнктива:** ***L. Conjunctiva:***	**OD**	**OS**
Норма/ *Normal*	1	1
Птеригиум/ *Pterygium*	2	2
Пингвекула/ *Pingecula*	3	3
Отек/ *Congestion*	4	4
Другое/ *Others*	5	5

**M. Роговица:** ***M. Cornea:***	**OD**	**OS**
Норма/ *Normal*	**1**	**1**
Помутнение/ *Opacity*	**2**	**2**
Язва/ *Ulcer*	**3**	**3**
Дегенерация/ *Degeneration*	**4**	**4**
Дистрофия/ *Dystrophy*	**5**	**5**
Отек/ *Edema*	**6**	**6**
Капельная дистрофия роговицы/ *Guttata*	**7**	**7**
Другое/ *Others*	**8**	**8**

Прозрачность роговицы / *corneal transparency*:

Прозрачная/*transparent*	**1**	**1**
Полупрозрачная/*Semi-transparent*	**2**	**2**
Непрозрачная/*Non-transparent*	**3**	**3**

**Вовлечение оптической зоны: /** ***Optical Zone Involvement***	**OD**	**OS**
Нет/ *No*	**1**	**1**
Частичное/ *Partly*	**2**	**2**
Полностью/ *Totally*	**3**	**3**

N. Передняя камера: /

Глубина перилимбальной передней камеры в % от прилежащей роговицы / ***LCD*** *as % thickness of adjacent cornea*	**Код** ***Codes***
0-5%	**1**
6-10%	**2**
11%-20%	**3**
21%-30%	**4**
31-50%	**5**
51-70%	**6**
71-100%	**7**

Anterior chamber:

	**OD**	**OS**
**Глубина передней камеры** / *AC Depth*		
LCD		
**PCD**		

2. Периферическая глубина передней камеры глаза (PCD)

Степень/ *Grade*		
I	Периферическая глубина передней камеры / < ½ толщины роговицы	*Peripheral Chamber Depth <1/2 Corneal Depth*
II	ПГПК <1/4 толщины роговицы	*PCD <1/4 Corneal Thickness Depth*
III	ПГПК >1/2толщины роговицы	*PCD >1/2 Corneal Thickness Depth or greater*
IV	ПГПК = толщине роговицы или больше	*PCD =Corneal Thickness Depth or greater*

	OD		OS	
	**Да /** ***Yes***	**Нет /** ***No***	**Да /** ***Yes***	**Нет /** ***No***
Клеточные элементы в передней камере / *Cells*	**1**	**2**	**1**	**2**
Опалесценция / *Flare*	**3**	**4**	**3**	**4**

О.Радужка / *O. Iris:*

	**OD**		**OS**	
Норма / *Normal*	1. Да/ *Yes*	2.Нет /*No*	1. Да/ *Yes*	2.Нет /*No*
Атрофия / *Atrophy*	1. Да/ *Yes*	2.Нет /*No*	1. Да/ *Yes*	2.Нет /*No*
Иридотомия / *Iridectomy*	1. Да/ *Yes*	2.Нет /*No*	1. Да/ *Yes*	2.Нет /*No*
Колобома / *Coloboma*	1. Да/ *Yes*	2.Нет /*No*	1. Да/ *Yes*	2.Нет /*No*
Задние синехии / *Post. Synechiae*	1. Да/ *Yes*	2.Нет /*No*	1. Да/ *Yes*	2.Нет /*No*
Выраженность рисунка / *Whorling*	1. Да/ *Yes*	2.Нет /*No*	1. Да/ *Yes*	2.Нет /*No*
Псевдоэксфолиация/ *Pseudoexfoliation*	1. Да/ *Yes*	2.Нет /*No*	1. Да/ *Yes*	2.Нет /*No*
Другое / *Others*	1. Да/ *Yes*	2.Нет /*No*	1. Да/ *Yes*	2.Нет /*No*

Р. Зрачок:. *Pupils:*

	OD	OS
Норма/ *Normal*	**1**	**1**
Относительный афферентный зрачковый дефект/ *Rapd*	**2**	**2**
Неправильный/ *Irregular*	**3**	**3**
Другое/ *Others*	**4**	**4**

Q. Хрусталик: *Q. Lens*:

	OD	OS
Факичный/ *Phakic*	**1**	**1**
Афакичный/ *Aphakic*	**2**	**2**
Переднекамерная ИОЛ/ *ACIOL*	**3**	**3**
Заднекамерная ИОЛ/ *PC IOL*	**4**	**4**

**Тип хирургии катаракты / *Type of cataract surgery***	OD	OS
Фемто + ФЭК/ Femtosecond laser-assisted phacoemulsification cataract	**1**	**1**
ФЭК/ Phacoemulsification cataract	**2**	**2**
ЭЭК/ Extracapsular cataract extraction (ECCE)	**3**	**3**
ИЭК/ Intracapsular cataract extraction (ICCE)	**4**	**4**

Дата операции / *Date of surgery*::___________________________________

Прозрачность задней капсулы, если была проведена хирургия катаракты: *Transparency of Posterior Capsule if cataract surgery was performed:*	OD	OS
Прозрачная/ *Clear*	**1**	**1**
Помутнение/ *Opacification*	**2**	**2**

Задняя капсулотомия/ *Capsulotomy*	**3**	**3**
Отсутствует / *Inapplicable*	**4**	**4**

	**OD**		**OS**	
**Фоторегистрация хрусталика** / *Photographic lens*	1. Да/ *Yes*	2.Нет /*No*	1. Да/ *Yes*	2.Нет /*No*

Степень прозрачности хрусталика

OD	OS
1 прозрачный / *no nuclear opacity in the lens*	1 прозрачный / *no nuclear opacity in the lens*
2 мягкое ядро / *soft nucleus*	2 мягкое ядро /*soft nucleus*
3 ядро малой плотности / *core of low density*	3 ядро малой плотности / *core of low density*
4 ядро средней плотности / *core of medium density*	4 ядро средней плотности / *core of medium density*
5 плотное ядро / *dense core*	5 плотное ядро / *dense core*
6 очень плотное ядро / *very dense core*	6 очень плотное ядро / *very dense core*

	OD		OS	
**Псевдоэксфолиативный синдром / Pseudoexfoliation of lens: PEX.**	1. Да/ *Yes*	2.Нет /*No*	1. Да/ *Yes*	2.Нет /*No*

**Описание**<br>*Description*	Степень<br>*Grade*<br>*OD*	*Степень*<br>*Grade*<br>*OS*
Слабый псевдоэксфолиативный синдром<br>(маленькие отдельные островки амилоидного материала на хрусталике в проекции зрачкового края радужки)<br>*Faint PEX (small islands in annular region corresponding to moving iris border*)	**2**	**2**
Сливающиеся островки амилоидного материала на хрусталике в проекции зрачкового края радужки, отвечающие на реакцию зрачка<br>*Confluenting islands in the annular region corresponding to the moving iris border.*	**3**	**3**
Граница псевдоэксфолиаций ясно обнаруживается, по крайней мере, в одном месте на поверхности линзы.<br>*Edge of pseudoexfoliative material clearly detectable in at least one location on the lens surface.*	**4**	**4**
Круговой край псевдоэксфолиаций на поверхности линз<br>*Circular edge of pseudoexfoliative material on the lens surface*	**5**	**5**
Псевдоэксфолиации по краю зрачка<br>*Pseudoexfoliative dandruff on the pupil margin*	**6**	**6**
Массивная псевдоэксфолиация, в том числе псевдоэксфолиативный материал на эндотелии роговицы, в углу передней камеры, или подвывих хрусталика<br>*Massive pseudoexfoliation including pseudoexfoliative material on corneal endothelium, in the AC angle, or lens subluxation*	**7**	**7**

	**OD**	**OS**
**R. 1. Внутриглазное давление (до мидриаза) (в мм рт.ст.)**<br>***IOP (before dilatation) (mm Hg)***		

S. ГОНИОСКОПИЯ / GONIOSCOPY:
S1. УГОЛ / ANGLE:

1 - Открыт на 360°<br>*360°Open*	**OD**	**OS**
2 - Закрыт < 90°		
3 - Закрыт 90°-180°		
4 - Закрыт 180°-270°		
5 - Закрыт > 270^0		
6 - Закрыт 360°		

S2. ПРИЧИНА ЗАКРЫТИЯ

OD			OS		
Прикрытие УПК радужкой (включая трабекулярный аппарат)<br>*Occludable*	Полное, плотное закрытие УПК радужкой<br>*Appositional*	Синехии в УПК<br>*Synechial*	Прикрытие УПК радужкой (включая трабекулярный аппарат)<br>*Occludable*	Полное, плотное закрытие УПК радужкой<br>*Appositional*	Синехии в УПК<br>*Synechial*
**1**	**2**	**3**	**1**	**2**	**3**

Pentacam

	**OD**	**OS**
Угол/ Angle		
Объем роговицы/ Cornea volume mm3		
Объем камеры/ Chamber volume mm3		
Диаметр зрачка/ Pupil dia mm		
Средний радиус кривизны передней поверхности роговицы/ Km- mid radius of curvature in the center		
Кератометрия слабого меридиана/ K1- keratometry flat radius D		
Кератометрия сильного меридиана/ K2- keratometry steep radius D		
Астигматизм/ Astigmatism in the center D		
Пахиметрия/ в центре зрачка Pachy: pupil center µm		
На вершине/ Pachy apex µm		
В самом тонком месте /Thinnest location µm		
К Макс (пер. пов.) (дптр)/ К Max D		
Элевация (пер. пов.) (мкт)/ Elevation (front surface)		
Элевация (зад. пов.) (мкт)/ Elevation (post. surface)		
Индекс поверхностной вариации/ index of surface variance (ISV)		
Индекс вертикальной асимметрии/ index of vertical assymetry (IVA)		

Индекс кератоконуса/keratoconus index (KI)		
Индекс центрального кератоконуса/ Center keratoconus index (CKI)		
Индекс высотной асимметрии/Index of height asymmetry(IHA)		
Индекс высотной децентрации/Index of height decentration (IHD)		
Наименьший радиус. Минимальная аксиальная сагитальная кривизна/Rmin Smallest radius(Rmin)		
Топографическая классификация кератоконуса. Стадия кератоконуса на основе данных передней поверхности роговицы/Topographical keratoconus classification (TKC)		

Расширение: с помощью циклопентолат 1%, тропикамида и фенилэфрина (5%),
Dilation: with cyclopentolate 1%, tropicamide & phenylephrine (5%), (**<u>если сделано, нужное подчекнуть</u>**)

**Фотографии глазного дна** / *Fundus Photographs:*	**1.Да/Yes**	**2.Нет/No**

**Невозможно обследовать по причине**/ *Reason*	OD	OS
Узкий зрачок/ *Small Pupil*	**1**	**1**
Непрозрачный хрусталик/ *Opaque непрозрачный*	**2**	**2**
Неконтактный пациент/ *Un co-operative*	**3**	**3**
Другое/ *Other*	**4**	**4**

	**OD**	**OS**
**R. 2. Внутриглазное давление (на фоне мидриаза) (в мм рт.ст.)** ***IOP (after dilatation) (mm Hg)***		
**Авторефракция (средняя) (на фоне мидриаза) / *Automated Refraction (Average)(after dilatation)***	Sph<br>Cyl<br>Ax	Sph<br>Cyl<br>Ax

U. Диск зрительного нерва (по снимкам): /
OPTIC DISC:

	**Параметры**	OD		OS	
		Да *Yes*	Нет *No*	Да *Yes*	Нет *No*
1	**Косой врез или децентрация экскавации / *Cup:Disc ratio***				
	Вертикальный/ /*Vertical*	**1**	**2**	**1**	**2**
	Горизонтальный / *Horizontal*	**1**	**2**	**1**	**2**
2	**Соответствие правилу ISNT, где нейроретинальный поясок (ободок рамка - область между краем ДЗН и краем экскавации) наиболее широкий снизу (inferior) и уменьшается в верхнем и назальном секторах, наименее широкая в носовой части (temporal) / *Obeying ISNT rule***	**1**	**2**	**1**	**2**
3	Явный дефект слоя нервных волокон в носовой части (temporal) / *Evident NFL Defect*	**1**	**2**	**1**	**2**
4	**Неравномерное расширение экскавации ДЗН в виде выемки (признак глаукоматозного поражения) Notching**				
	Расширение экскавации в верхнем секторе ДЗН / Superior	**1**	**2**	**1**	**2**

	Расширение экскавации в нижнем секторе ДЗН / Inferior	**1**	**2**	**1**	**2**
	Расширение экскавации в височном секторе / Temporalis	**1**	**2**	**1**	**2**
	Расширение экскавации в носовом секторе / Nasalis	**1**	**2**	**1**	**2**
5	**Смещение сосудистого пучка ДЗН в носовую сторону** ***Nasal Shifting***	**1**	**2**	**1**	**2**
6	**Резкий острый изгиб сосудов при пересечении области ямки ДЗН / Bayonetting**	**1**	**2**	**1**	**2**
7	**Кровоизлияния / Haemorrhage**	**1**	**2**	**1**	**2**
8	**Коллатеральные сосуды / Collaterals**	**1**	**2**	**1**	**2**
9	**Другие признаки/Other**	**1**	**2**	**1**	**2**

ОКТ ДЗН/
OPTIC DISC OCT

Перипапиллярная толщина слоя нервных волокон/ Peripapillary thickness of nerve fibres	OD	OS
Средние знач-ия/Whole ave		
Височная/Temporalis		
Верхняя/Superior		
Носовая/Nasalis		
Нижняя/Inferior		
**Nidek, Disc Map**		
C_D_Horizon		
C_D_Vertical		
R_D_Min		
R_D_Angle		
Disc_Area_mm2		
Cup_Area_mm2		

**По Тритону 3D disc, толщина слоя нервных волокон (RNFL) по 12 часовым секторам**	OD	OS
Triton_RNFL_1_Superior_hour12_00		
Triton_RNFL_2_hour13_00		
Triton_RNFL_3_hour14_00		
Triton_RNFL_4_hour15_00		
Triton_RNFL_5_hour16_00		

Triton_RNFL_6_hour17_00		
Triton_RNFL_7_Inferior_hour_18_00		
Triton_RNFL_8_hour19_00		
Triton_RNFL_9_hour20_00		
Triton_RNFL_10_hour21_00		
Triton_RNFL_11_hour22_00		
Triton_RNFL_12_hour23_00		

**По Тритону 3D disc, толщина слоя хориокапилляров (CSI) по 12 часовым секторам**	OD	OS
Triton_CSI_1_Superior_hour12_00		
Triton_CSI_2_hour13_00		
Triton_CSI_3_hour14_00		
Triton_CSI_4_hour15_00		
Triton_CSI_5_hour16_00		
Triton_CSI_6_hour17_00		
Triton_CSI_7_Inferior_hour_18_00		
Triton_CSI_8_hour19_00		
Triton_CSI_9_hour20_00		
Triton_CSI_10_hour21_00		
Triton_CSI_11_hour22_00		
Triton_CSI_12_hour23_00		

V. Макула (по снимкам): / *V. MACULA:*

Оптическая плотность макулярного пигмента/ Macular pigment optic density

	OD	*OS*
Объем/Volume		
Площадь/Area		
Максимальная оптическая плотность/Max optical density		
Средняя оптическая плотность/Mean optical density		

Измерение толщины сетчатки по слоям / Measurement of the thickness of the retinal layers	OD			OS		
	300 µm temporal	Fovea	300 µm nasal	300 µm temporal	Fovea	300 µm nasal
Внутренняя пограничная мембрана.-ПЭС / Базальная мембрана / **ILM-RPE/BM**						
ПЭС/Базальная мембрана – Наружные и внутренние сегменты фоторецепторов / **RPE/BM-IS/OS**						
Наружные и внутренние сегменты фоторецепторов – Наружный плексиформный слой / Наружный ядерный слой / **IS/OS-OPL/ONL**						
Наружный плексиформный слой / Наружный ядерный слой-Внутренний плексиформный слой/Внутренний ядерный слой / **OPL/ONL-IPL/INL**						
Внутренний плексиформный слой / Внутренний ядерный слой – Слой нервных волокон / Слой ганглионарных клеток / **IPL/INL-NFL/GCL**						
Слой нервных волокон / Слой ганглионарных клеток – Внутренняя пограничная мембрана / **NFL/GCL-ILM**						
Толщина хороидеи в фовеа на «Тритоне» / **Choroidal thickness on «Triton»**						

Центральная зона сетчатки / Retina

		**OD**		**OS**	
**V1**	Норма/ / *Normal*	1. Да/ *Yes*	2.Нет /*No*	1. Да/ *Yes*	2.Нет /*No*
**V2**	Друзы/ / *Druzen*	1. Да/ *Yes*	2.Нет /*No*	1. Да/ *Yes*	2.Нет /*No*
**V3**	Витреомакулярная тракция/ *Vitreomacular traction*	1. Да/ *Yes*	2.Нет /*No*	1. Да/ *Yes*	2.Нет /*No*

**V4**	Витреомакулярная адгезия/<br>*Vitreomacular adhesion*	1. Да/ *Yes*	2.Нет /*No*	1. Да/ *Yes*	2.Нет /*No*
**V5**	Хороидальная неоваскуляризация/<br>*Choroid neovascularization*	1. Да/ *Yes*	2.Нет /*No*	1. Да/ *Yes*	2.Нет /*No*
**V6**	Эпиретинальная мембрана/<br>*Epyretinal membrane*	1. Да/ *Yes*	2.Нет /*No*	1. Да/ *Yes*	2.Нет /*No*
**V7**	Ламеллярный макулярный разрыв/<br>*Lamellar macular rupture*	1. Да/ *Yes*	2.Нет /*No*	1. Да/ *Yes*	2.Нет /*No*
**V8**	Сквозной макулярный разрыв/<br>*Macular hole*	1. Да/ *Yes*	2.Нет /*No*	1. Да/ *Yes*	2.Нет /*No*
**V9**	Отек сетчатки фокальный/<br>*Focal oedema of retina*	1. Да/ *Yes*	2.Нет /*No*	1. Да/ *Yes*	2.Нет /*No*
**V10**	Отек сетчатки диффузный/<br>*Diffuse oedema of retina*	1. Да/ *Yes*	2.Нет /*No*	1. Да/ *Yes*	2.Нет /*No*
**V11**	Отек сетчатки кистозный/<br>*Cystic oedema of retina*	1. Да/ *Yes*	2.Нет /*No*	1. Да/ *Yes*	2.Нет /*No*
**V12**	Отек сетчатки тракционный/<br>*Traction oedema of retina*	1. Да/ *Yes*	2.Нет /*No*	1. Да/ *Yes*	2.Нет /*No*
**V13**	Отек сетчатки смешанный/<br>*Mixed oedema of retina*	1. Да/ *Yes*	2.Нет /*No*	1. Да/ *Yes*	2.Нет /*No*
**V14**	Твердые экссудаты/<br>*Soled exudates*	1. Да/ *Yes*	2.Нет /*No*	1. Да/ *Yes*	2.Нет /*No*
**V15**	Серозная отслойка нейроэпителия/<br>*Serous detachment of neuroepithelium*	1. Да/ *Yes*	2.Нет /*No*	1. Да/ *Yes*	2.Нет /*No*
**V16**	Куполообразная макула/<br>*Dome-shaped macula*	1. Да/ *Yes*	2.Нет /*No*	1. Да/ *Yes*	2.Нет /*No*
**V17**	Центральная дистрофия колбочек/<br>*Central rod cells dystrophy*	1. Да/ *Yes*	2.Нет /*No*	1. Да/ *Yes*	2.Нет /*No*
**V18**	Периферическая дистрофия палочек и колбочек/<br>*Periphery dystrophy of rod and cone cells*	1. Да/ *Yes*	2.Нет /*No*	1. Да/ *Yes*	2.Нет /*No*
**V19**	Дистрофия Штаргардта/<br>*Stargart dystrophy*	1. Да/ *Yes*	2.Нет /*No*	1. Да/ *Yes*	2.Нет /*No*
**V20**	Атрофия пигментного эпителия сетчатки/<br>*Pigment epithelium of retina atrophy*	1. Да/ *Yes*	2.Нет /*No*	1. Да/ *Yes*	2.Нет /*No*
**V21**	Полипоидная васкулопатия/<br>*Polipoid vasculopathy*	1. Да/ *Yes*	2.Нет /*No*	1. Да/ *Yes*	2.Нет /*No*
**V22**	Дистрофия пигментного эпителия сетчатки/<br>*Pigment epythilium of retina dystrophy*	1. Да/ *Yes*	2.Нет /*No*	1. Да/ *Yes*	2.Нет /*No*

W. Общая информация по глазному дну (по снимкам) / *General Fundus:*

	OD	OS
Норма / *Normal*	**1**	**1**
Отклонение от нормы / *Abnormal*	**2**	**2**

X. Основная причина потери зрения: / *Major Cause of visual loss:*

	OD	OS
Первая / *First*		
Вторая / *Second*		
Третья / *Third*		

**Роговица / конъюнктива,**<br>*Corneal / Conjunctival*	**1**
**Катаракта/** *Cataract*	**2**
**Возрастная макулярная дегенерация/** *AMD*	**3**
**Глаукома/** *Glaucoma*	**4**
**Диабетическая ретинопатия** / *Diabetic Retinopathy*	**5**
**Другие заболевания сетчатки и зрительного нерва/** *Other diseases of retina or optic nerve*	**6**
**Амблиопия** / *Amblyopia*	**7**
**Нистагм** / *Nystagmus*	**8**
**Недостоверные данные остроты зрения** / *Unreliable VA testing*	**9**
**Другое/** *Other*	**10**

**НЕОБХОДИМОЕ ЛЕЧЕНИЕ**<br>*Treatment necessary:*	OD		OS	
**Нет** / *None*	1. Да/ *Yes*	2.Нет /*No*	1. Да/ *Yes*	2.Нет /*No*
**Очки/** *Glasses*	1. Да/ *Yes*	2.Нет /*No*	1. Да/ *Yes*	2.Нет /*No*
**Хирургия век/** *Eyelid Surgery*	1. Да/ *Yes*	2.Нет /*No*	1. Да/ *Yes*	2.Нет /*No*
**Катаракта/** *Cataract*	1. Да/ *Yes*	2.Нет /*No*	1. Да/ *Yes*	2.Нет /*No*
**Глаукома**/ *Glaucoma*	1. Да/ *Yes*	2.Нет /*No*	1. Да/ *Yes*	2.Нет /*No*
**Диабетическая ретинопатия**/ *Diabetic Retinopathy*	1. Да/ *Yes*	2.Нет /*No*	1. Да/ *Yes*	2.Нет /*No*
**Другое**/ *Others*	1. Да/ *Yes*	2.Нет /*No*	1. Да/ *Yes*	2.Нет /*No*

Лист постановки диагноза

ФИО врача-исследователя	Метод обследования	Результат (отметить при патологии)	
		OD	OS
	Острота зрения по таблице Сивцева	С максимальной коррекцией ________	С максимальной коррекцией ________
	Тонометрия бесконтактная		
	Кератотопограф Pentacam		
	Оптическая когерентная томография		
	Фоторегистрация глазного дна		
	Компьютерная периметрия		
	Фоторегистрация переднего отрезка глаза		
	Ульразвуковая биометрия	ПЗО________________	ПЗО________________

Диагноз старшего врача (предварительный):

____

____

____

Диагноз консилиума:

____

____

____

Проверено доктором (ФИО) / *Checked by dr:*____

Подпись / *signature*:____
